# The history of chromosomal instability in genome doubled tumors

**DOI:** 10.1158/2159-8290.CD-23-1249

**Published:** 2024-10-04

**Authors:** Toby M. Baker, Siqi Lai, Andrew R. Lynch, Tom Lesluyes, Haixi Yan, Huw A. Ogilvie, Annelien Verfaillie, Stefan Dentro, Amy L. Bowes, Nischalan Pillay, Adrienne M. Flanagan, Charles Swanton, Paul T. Spellman, Maxime Tarabichi, Peter Van Loo

**Affiliations:** 1 The Francis Crick Institute, London, UK; 2 Department of Genetics, The University of Texas MD Anderson Cancer Center, Houston, Texas, USA; 3 Department of Medicine, University of California Los Angeles, Los Angeles, California, USA; 4 Division of AI in Oncology, German Cancer Research Centre DKFZ, Heidelberg, Germany; 5 Research Department of Pathology, Cancer Institute, University College London, London, UK; 6 Department of Cellular and Molecular Pathology, Royal National Orthopaedic Hospital NHS Trust, Stanmore, UK; 7 Cancer Research UK Lung Cancer Centre of Excellence, University College London Cancer Institute, London, UK; 8 Department of Oncology, University College London Hospitals, London, UK; 9 Institute for Interdisciplinary Research (IRIBHM), Université Libre de Bruxelles, Brussels, Belgium; 10 Department of Genomic Medicine, The University of Texas MD Anderson Cancer Center, Houston, Texas, USA

**Keywords:** Tumor evolution, chromosomal instability, gain timing, molecular archeology of cancer, whole genome duplication, copy number gains

## Abstract

Tumors frequently display high chromosomal instability and contain multiple copies of genomic regions. Here, we describe GRITIC, a generic method for timing genomic gains leading to complex copy number states, using single-sample bulk whole-genome sequencing data. By applying GRITIC to 6,091 tumors, we found that non-parsimonious evolution is frequent in the formation of complex copy number states in genome-doubled tumors. We measured chromosomal instability before and after genome duplication in human tumors and found that late genome doubling was followed by an increase in the rate of copy number gain. Copy number gains often accumulate as punctuated bursts, commonly after genome doubling. We infer that genome duplications typically affect the landscape of copy number losses, while only minimally impacting copy number gains. In summary, GRITIC is a novel copy number gain timing framework that permits the analysis of copy number evolution in chromosomally unstable tumors.

## Introduction

Genomic copy number gains and losses, caused by chromosomal instability (CIN), are common somatic alterations in cancer (1,2). While somatic single nucleotide variants (SNVs) and indels linked to cancer drivers are found in ostensibly healthy tissues, copy number events rarely occur in normal cells (3–6). Identifying when copy number events occur is important for screening purposes and for gaining an understanding of the key molecular mechanisms underlying cancer development.

Of particular interest is the evolution of copy number events in tumors with the most aberrant genomes, as CIN is linked to poorer outcomes (7). Tumors that have undergone whole genome duplication (WGD) often show elevated numbers of copy number gains and losses (8–10) which may arise through multiple mechanisms, including chromosomal missegregation from centrosomal amplification (11) and a shortage of replication machinery proteins immediately following WGD (12). While copy number gains and losses support further CIN, the temporal relationship between WGDs and CIN is difficult to assess from single-timepoint biopsies in human tumors. The extent of genomic aberration, often used as an indirect proxy for CIN, does not convey the temporal dynamics that define CIN.

To observe the evolution of genomic gains in genome-doubled tumors, the timing of copy number gains and WGDs relative to the accumulation of SNVs can be inferred from whole-genome sequencing data (13–15). Clonal copy number gains, which are present in every tumor cell, can be placed on a timeline from 0 to 1, where 0 represents conception and 1 represents the end of the tumor’s clonal evolutionary period. Previous approaches that have used this principle to time copy number gains (16–18) were unable to fully time gains leading to complex copy number states (those with three or more copies of one parental allele). This is due to higher ambiguity in the route history of these complex states relative to simpler states. Either the most parsimonious route history was assumed (17,18) or these states were not timed at all (16). Recently, two new methods have been developed to time much more complex states than previous approaches, but they either only provide bounds on the timing of the first and last gains for a segment (19), or still require an assumption of parsimony (20).

Here, we present GRITIC (Gain Route Identification and Timing In Cancer), a method that can time sequential gains leading to complex clonal copy number states, thereby elucidating the genome-wide evolution of gains in tumors with high CIN. As GRITIC is designed to time clonal copy number gains, it is well suited to unravel the evolution of the earliest genomic events in tumors, those that arise before the emergence of the tumor’s most recent common ancestor. After filtering for minimum sample quality and WGD status, we applied GRITIC to a cohort of 1,751 primary tumors from the Pan-Cancer Analysis of Whole Genomes (PCAWG) dataset (21) and 4,340 metastases from the Hartwig Medical Foundation dataset (22). Surprisingly, we observed that the commonly held principle of maximum parsimony (i.e., that copy number states are formed through the simplest possible route) is frequently violated for complex copy number gains in WGD tumors. We found that punctuated bursts of gains, independent of WGD, were common across cancer types. We infer the rate of gains pre- and post-WGD across our cohort and observe that late WGD causes an immediate increase in the rate of gains, a proxy for the rate of chromosomal instability. By considering the landscape of copy number events before and after a WGD, we found that WGD appears to have a low impact on the landscape of copy number gains, but a greater impact on losses.

## Results

### GRITIC leverages SNVs to time complex copy number gains

**Fig. 1:**
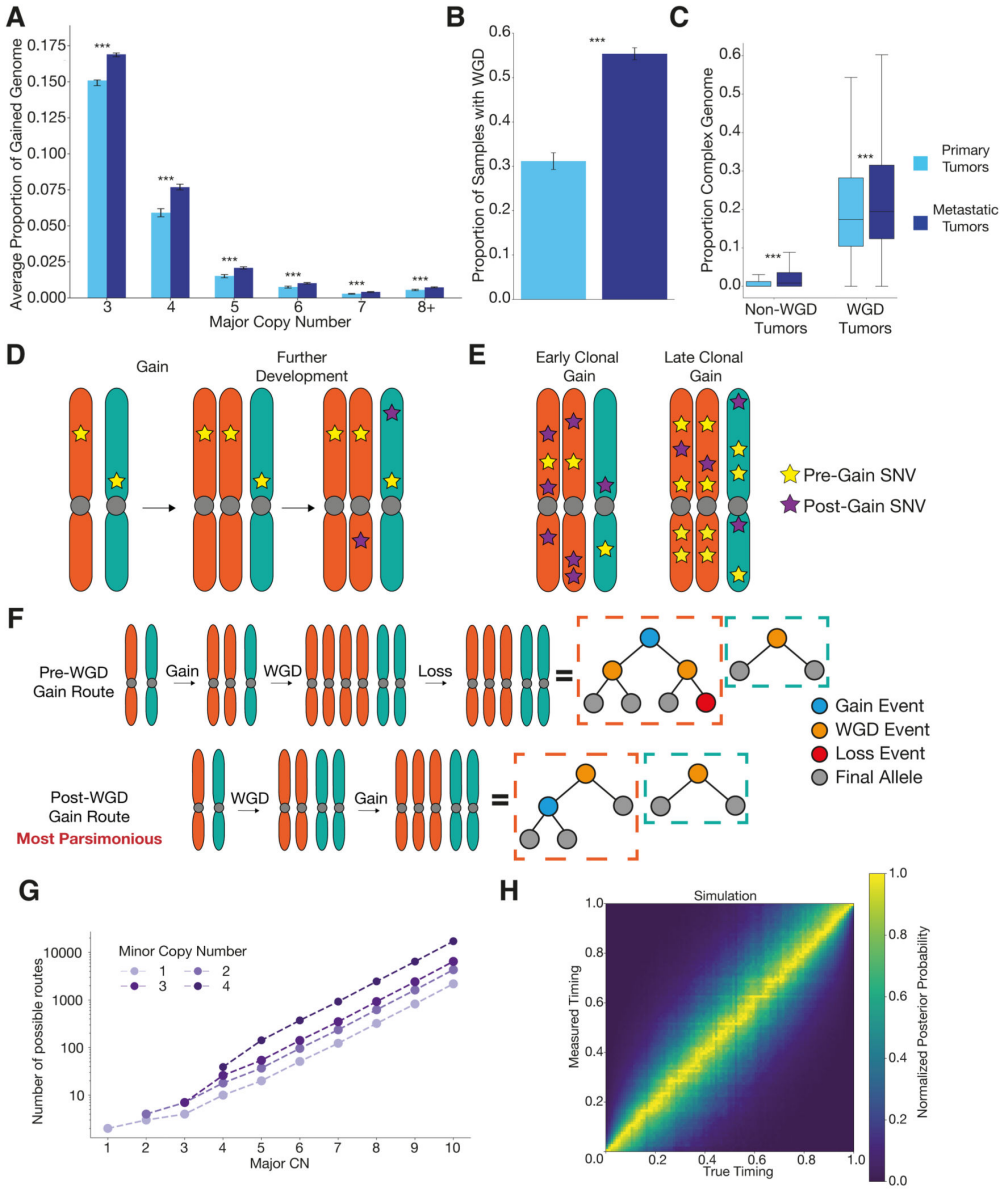
Principles of timing complex copy number gains. **A**, Average proportion of the genome with different major copy number states split by primary and metastatic cohorts. Statistical significance was calculated by permutation test and 95% confidence intervals by bootstrapping over samples. **B**, Proportion of tumor samples identified as WGD in primary and metastatic cohorts. Statistical significance is calculated by proportion test and 95% confidence intervals by normal approximation to a binomial proportion. **C**, Proportion of the genome with a major copy number of at least three in the primary and metastatic cohorts, split by WGD status. Statistical significance was calculated by permutation test and 95% confidence intervals by bootstrapping over samples. **D**, Schematic showing SNVs on a gained allele are duplicated by the gain, the principle underlying copy number gain timing. **E**, Schematic showing the difference in SNVs on multiple copies between a gain that occurs early in the clonal evolutionary period and a gain that occurs later. **F**, Binary tree representation of two possible routes that result in a 3+2 copy number state in a WGD tumor. The post-WGD route is the most parsimonious as it involves the fewest events. **G**, The number of theoretically distinguishable unique routes that can result in different allele-specific copy number states given a single WGD. **H**, Distribution of measured posterior probability on gain timing against true gain timing across a representative simulated cohort of complex gains. *** p < 0.001.

Tumors frequently gain additional copies of their genomic regions. In the Hartwig and PCAWG datasets, copy number gains affected an average of 48.6% of the tumor genomes (55.5% and 33.0%, respectively). Complex gains were common, with 27.8% of the gained genome having three or more copies of one parental allele on average (28.8% and 24.1% for Hartwig metastases and PCAWG primary tumors, respectively, Fig. 1A). The frequency of a given complex copy number state was inversely correlated with the largest number of copies of the parental allele for the state, known as the major copy number (Fig. 1A). Metastases also had a higher rate of WGD in our cohorts: 55.3% for Hartwig metastases compared to 31.2% for PCAWG primary tumors (Fig. 1B, S1A), although this appears to be a phenomenon specific to certain cancer types (Fig. S1B) (8,22). Although the difference in complex copy number fraction is largely explained by the higher proportion of WGD tumors, it was still higher in metastases when controlling for WGD frequency (Fig. 1C), likely reflecting increased CIN in metastatic cancers.

Clonal copy number gains can be quantitatively timed by considering SNVs in the gained region. When a gain occurs, all SNVs present on the gained allele are duplicated on the new copy (Fig. 1D). With the reasonable assumption that each base pair in the genome is mutated at most once (23), any SNV on multiple copies in a gained region must have occurred before the copy number gain. This principle can be used to infer the timing of the gain (14) (Fig. 1E, Supplementary Methods). However, for more complex gains, further consideration of the possible routes that lead to these complex states is required. To accomplish this, we developed GRITIC, a new method that can identify, distinguish, and time the gains in these routes.

GRITIC uses a binary tree representation, conceptually similar to an earlier approach (24), to represent the gain history of a given segment. These representations can be used to calculate all possible routes (assuming, at most, a single WGD), resulting in a particular copy number state (Fig. 1F, Supplementary Methods). We found that the number of possible routes increases exponentially with the complexity of the copy-number state (Fig. 1G). Therefore, we limit GRITIC to the timing of copy number gains of segments with no more than 500 possible routes for WGD tumors (Supplementary Table 1). GRITIC uses a Bayesian Markov Chain Monte Carlo (MCMC) approach to infer the posterior probability of all possible route histories and the corresponding set of gain timings from SNV read counts for each gained segment. GRITIC is particularly suited to timing tumors with a WGD, as it uses the simultaneous occurrence of a WGD across all genomic regions as a constraint during inference to improve timing accuracy (Fig. S2A-B, Methods).

We applied GRITIC to a realistic simulated cohort of WGD tumors (Methods) and found that with simulated tumor purity and sequencing coverage representative of the tumors in PCAWG and Hartwig, GRITIC can accurately measure the timing of all gains leading to complex states under different parsimony assumptions (Fig. 1H, S3-S7, Methods). Although more sensitive to simulation and inference conditions compared to measuring the gain timing itself, GRITIC can also accurately estimate the probabilities of different gain routes (Fig. S8-S11). As further validation, we tested GRITIC on patients with multiple samples in the Hartwig cohort and confirmed that shared gains (expected to have occurred earlier) showed earlier timing than gains unique to one sample (expected to have occurred later) in 78.9% (142/180) of cases (Fig. S12A-B).

### Non-parsimony is common in WGD tumor gain evolution

**Fig. 2:**
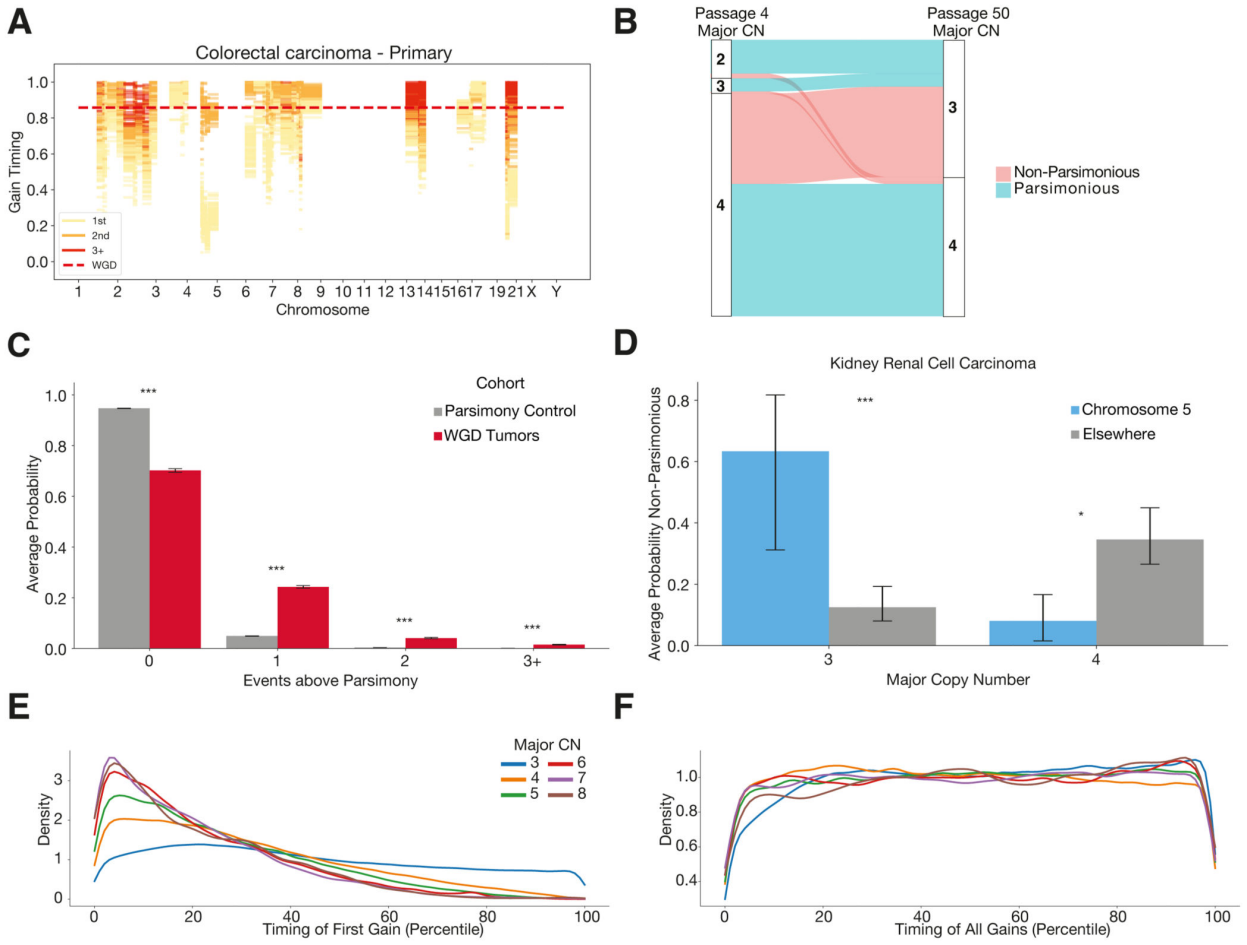
Non-parsimonious copy number evolution in cancer. **A**, Example posterior distribution of copy number gain timing in a whole-genome duplicated sample from Hartwig with GRITIC. 100 independent draws from the posterior sample for each gained segment are shown. **B**, The proportion of different copy number states at passage 4 that result in copy number states with a major copy number of 3 or 4 at passage 50 in four tetraploid clones in a colorectal cancer cell line. The flows are colored by whether the transition would be considered a parsimonious route by the copy number at passage 50. **C**, The average posterior probability on the number of additional events required to reach the final state over the most parsimonious route for complex gained states in the PCAWG and Hartwig cohort, compared to a simulated control where only parsimonious routes were included. A penalty on non-parsimony was applied during inference. Statistical significance is calculated with a permutation test and 95% confidence intervals by bootstrapping over samples. **D**, The average probability on non-parsimonious routes for gained segments in clear cell renal cell carcinoma, split by major copy number and gain location. A penalty on non-parsimony was applied during inference. Statistical significance was calculated with a permutation test and 95% confidence intervals were calculated by bootstrapping over samples. **E**, The timing distribution of the first gains in complex segments relative to other gains in the same sample, as defined by their quantile ranking within each sample, split by major copy number. **F**, The timing distribution of all first gains in complex segments relative to other gains in the same sample, as defined by their quantile ranking within each sample, split by major copy number. * p < 0.05; *** p < 0.001.

We then applied GRITIC to time the gains that led to 164,062 clonally gained regions across 6,091 tumors in the PCAWG and Hartwig datasets that pass our quality filters (Supplementary Methods). GRITIC reconstructs the timing of both the independent gains and the WGD (if present) in each sample and can time multiple sequential independent gains in the same genomic region (Fig. 2A). GRITIC produces a joint posterior distribution over gain timing and route histories considering all possible routes for each segment. Although different routes have distinct relationships between SNV multiplicity and gain timing, the timing of gains is generally concordant between different routes (Fig. S13-S14).

Parsimonious route histories have often been assumed for the development of copy number states in WGD tumors (17,18). This is because the total number of allelic copies gained through WGD *versus* individual independent gains will vary between different routes, as will the number of losses required to make the route self-consistent (Supplementary Methods). Therefore, we sought to evaluate the assumption of parsimonious evolution in WGD tumors.

We first tested this assumption by reanalyzing copy number data from isogenic tetraploid colorectal cancer HCT-116 cell lines obtained from two different passages derived from a diploid progenitor (25,26). By considering the change in copy number states between the two passages, we found that most events with a major copy number of three in the later passage arose through complex routes that would violate the assumption of parsimony if applied to the later passage in isolation (Fig. 2B). This result suggests that the assumption of parsimonious copy number evolution is often invalid.

We next used GRITIC to test this parsimonious evolution assumption. Owing to the inherent uncertainties in estimating copy number gain routes from SNVs (Supplementary Methods), GRITIC assigns an average posterior probability of 56.2% to non-parsimonious routes from a simulated set of tumors with completely parsimonious evolutionary histories (Fig. S15A, S16). Although the model evidence used in GRITIC provides a natural penalty against additional independent gain timing parameters, it does not penalize the number of losses implied by a given route. Thus, to ensure a conservative estimate of non-parsimony, we applied a penalty term to the number of events required for each route in WGD tumors (Methods). This penalty was fitted such that the average posterior probability of non-parsimonious routes was ~5% on a representative cohort of simulated WGD tumors with only parsimonious routes (Fig. S17-S18).

With this penalty term, we evaluated non-parsimonious evolution across genome-doubled tumors. Surprisingly, we found that non-parsimony was common: 29.8% of the total posterior probability on gained segments in WGD tumors was on non-parsimonious route histories, with 5.6% on routes with two or more additional events compared to the simplest route (Fig. 2C, S15B, S16, p<0.001, permutation test). Owing to our conservative penalty term, these are likely underestimates of non-parsimony in copy number evolution.

Non-parsimony occurs in agreement with known phenomena. Gains on chromosome 5q are known to be an initiating event in clear cell renal cell carcinoma, combined with the loss of 3p (27). Indeed, we found that 52/54 of the genome-doubled clear cell renal cell carcinomas had LOH loss of 3p, indicating that it likely occurred pre-WGD. In line with this, GRITIC inferred that gains on chromosome 5 with a major copy number of 3 are significantly more non-parsimonious (*i.e*. earlier, Fig. S15C, Supplementary Methods) than the background for clear cell renal cell carcinoma (Fig. 2D, S19, p<0.001, permutation test). Conversely, gains on chromosome 5 with a major copy number of four were more likely parsimonious (*i.e*. earlier, Fig. 2D, S15C, p<0.05, permutation test). This effect was also observed when a non-parsimony penalty term was not applied (Fig. S15D-E). More generally, we found that the frequency of pre-WGD gains for major copy number 3 and 4 states was correlated, both for segments within the same chromosome and across different samples (Fig. S20-S21).

Applying a penalty to the number of events provides a conservative estimate on the non-parsimonious evolution of complex gains. However, we found that, as expected, this causes the inferred probability of non-parsimonious evolution to be inaccurate for cohorts simulated to contain non-parsimonious routes (Fig. S9). Therefore, for all subsequent analyses, we show the results of applying GRITIC without this non-parsimony penalty term and display the results with a penalty term in the Supplementary Information. In general, despite different route probabilities, we find that the results are highly consistent.

We find that the initial gains that occur independently of the WGD tend to occur earlier as the major copy number increases (Methods, Fig. 2E, S22A-C). In contrast, the timing of all copy number gains that occur independently of WGD are much more uniformly distributed across mutation time for all copy number states (Fig. 2F, S22). This suggests that moderate-level amplifications in tumor development generally begin early but accumulate further gains throughout the clonal evolutionary period.

### The effect of genome doubling on the rate of chromosomal instability

**Fig. 3:**
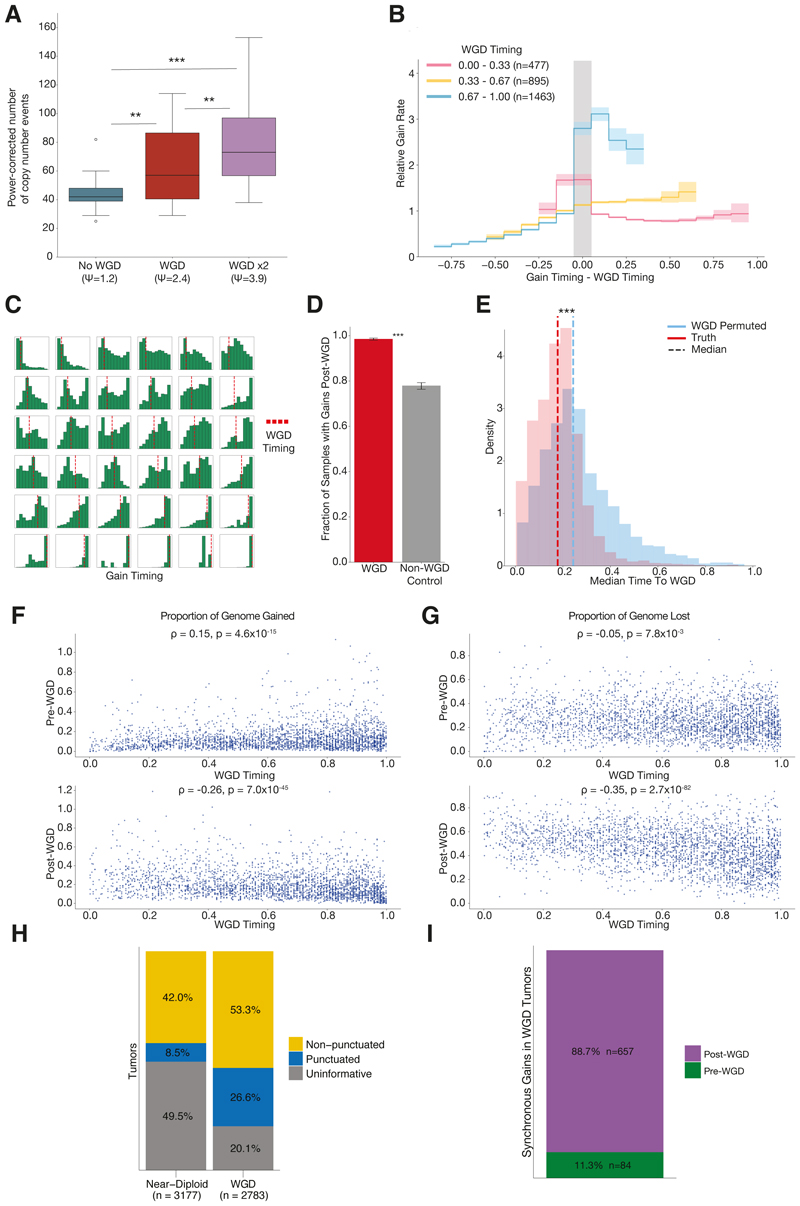
The history of chromosomal instability over tumor evolution. **A**, The number of copy number events required to reach the final copy number state from the most recent common ancestor from a collection of tumor cells with different ploidy states from a single undifferentiated sarcoma. The number of events is normalized for ploidy and statistical significance is calculated with a Mann-Whitney U test. Ψ denotes population ploidy as determined by FACS. **B**, The normalized rate of gains relative to the WGD timing across cancer types. Statistical significance is calculated with a permutation test and 95% confidence intervals are calculated by bootstrapping over samples. **C**, Histograms of gain timing in a random selection of tumors with a WGD. Samples are ordered by independent gain timing from left to right and WGD timing from top to bottom. **D**, Proportion of samples with gains post-WGD for WGD tumors and a cohort of control non-WGD tumors with a pseudo-WGD timing randomly sampled from WGD tumors with the same cancer type. Statistical significance is calculated with a permutation test and 95% confidence intervals are calculated by bootstrapping over samples. **E**, Distribution of the mean mutation time between the timing of all independent gains and the median WGD timing for each genome duplicated sample. Two cohorts are displayed, one with the correct WGD timing and another where the WGD timing is permuted between samples of the same cancer type. Statistical significance calculated by Mann-Whitney U test. **F**, Proportion of genome gained before and after WGD against WGD timing for genome doubled tumors. **G**, Proportion of genome lost before and after WGD against WGD timing for genome doubled tumors. **H**, Proportion of tumors with clonal gains identified as occurring in a punctuated burst, or uninformative where the number of gains was too low to classify, split by WGD status. **I**, Proportion of punctuated gains occurring in WGD samples, classified by whether they occurred pre- or post-WGD. ** p < 0.01; *** p < 0.001.

Next, we evaluated the effect of genome doubling on the rate of CIN. We analyzed the copy number profiles of 260 individual tumor cells in an undifferentiated soft tissue sarcoma that had undergone consecutive subclonal WGDs, as shown experimentally (Supplementary Methods). We found progressively higher rates of inter-copy number diversity in cells with each round of WGD (Fig. 3A, S23A-C, p<0.001, Mann-Whitney U Test, Methods), suggesting that WGD increases CIN even within the same tumor.

We used GRITIC to calculate the rate of independent gains, a proxy for the rate of CIN, relative to the occurrence of WGD across our cohort (Methods). We found that the gain rate increased after WGD in tumors with late genome doubling (Fig. 3B, S24). Interestingly, the tumors with the earliest genome doubling show a different trend, exhibiting a higher gain rate before early WGD and a subsequent lower post-WGD gain frequency (Fig. 3B), an effect greater than expected from samples simulated with a uniform rate of gains (Fig. S25, Supplementary Methods). This behavior may be driven primarily by breast tumors, which were enriched for early genome doubling (Fig. S24), consistent with reports of highly aneuploid karyotypes of early precursor lesions of breast cancers (28). However, both trends were generally conserved when considering individual cancer types with a sufficient number of samples (Fig. S24). While difficult to observe in individual tumors (Fig. 3C, S26), the effect of WGD on CIN in aggregate was clear.

The rate of copy number gain accumulation increases over the clonal evolutionary period for both WGD and non-WGD tumors (Fig. S27). Nevertheless, post-WGD gains occurred more frequently than could be explained by the increase in CIN over tumor development that is also present in non-WGD tumors. The proportion of WGD tumors that had a gain post-WGD (98.5%) was significantly higher than in a control cohort of non-WGD tumors each given a realistic pseudo-WGD timing (78.0%, p<0.001, permutation test, Fig. 3D, S28A-B, Methods). Moreover, the mean mutational time between independent gains and WGD was significantly lower than expected from permuting WGD timing across samples from the same cancer type (p<0.001, Mann-Whitney U Test, Fig. 3E, S28C-D, S29, Methods).

The later a WGD occurs, the less time there is for gains to accumulate post-WGD. Correspondingly, the amount of genome gained post-WGD was negatively correlated with WGD timing (Fig. 3F, S30A-B). Similarly, the genomic material gained pre-WGD was weakly positively correlated with WGD timing (Fig. 3F). There was also a stronger negative correlation between WGD timing and the proportion of genome lost post-WGD (Fig. 3G, S30C). Generally, these trends are conserved when cancer types are considered separately and after applying the non-parsimony penalty (Fig. S31-S33). The high frequency of post-WGD losses is consistent with the widespread hypothesis that, in many cancers, WGD serves as an evolutionary intermediate to fitter sub-tetraploid karyotypes (29).

In contrast, there was very little correlation between WGD timing and the proportion of the genome that was lost pre-WGD. This suggests that such losses pre-WGD either do not accumulate steadily with respect to SNV accumulation, or are linked to WGD itself (Fig. 3G). This could support a model whereby a major advantage of WGDs is the mitigation of the deleterious effect of mutations in regions with copy number losses, as reported by Lopez *et al*. (26). Compared to non-WGD tumors, WGD tumors have a higher proportion of genomic LOH (26). This suggests that rather than SNV accumulation, a high level of genomic loss could lead to WGD, which may explain our results.

### Punctuated gain evolution in WGD tumors

Next, we sought to better understand the distribution of gain timing in WGD samples. We previously found that copy number gains in non-WGD tumors often occur as punctuated events (17). Indeed, using GRITIC, we found that gains in 16.9% of informative non-WGD samples (those with gains affecting at least three separate chromosomes) occurred over significantly shorter timespans than expected under a permutation model (Fig. 3H, S34A-D, Methods).

Using GRITIC, we can now study the timing of gains that arise independently of the WGD. We found that gains in 33.3% of informative WGD tumors occurred significantly closer in time than expected from permutations (Fig. 3H, S34A-E). Most of these (88.7%) occurred post-WGD (Fig. 3I, S34F), suggesting that WGD may increase the likelihood of or tolerance to punctuated bursts of gains (25). Together, these results suggest that copy number gains occur frequently in punctuated bursts, even in the most chromosomally unstable samples. These punctuated bursts likely explain why some tumors with late WGD still accumulate most clonal gains post-WGD (Fig. S30A-B). We observed no significant association between tumors with chromothripsis and those with punctuated gains (Fig. S35A-B), suggesting that the two processes are unrelated. We also observed similar segment size distributions for punctuated and non-punctuated gains (Fig. S36A-D, Methods), suggesting similar underlying mechanisms.

### Measuring the impact of genome doubling on the copy number landscape

**Fig. 4:**
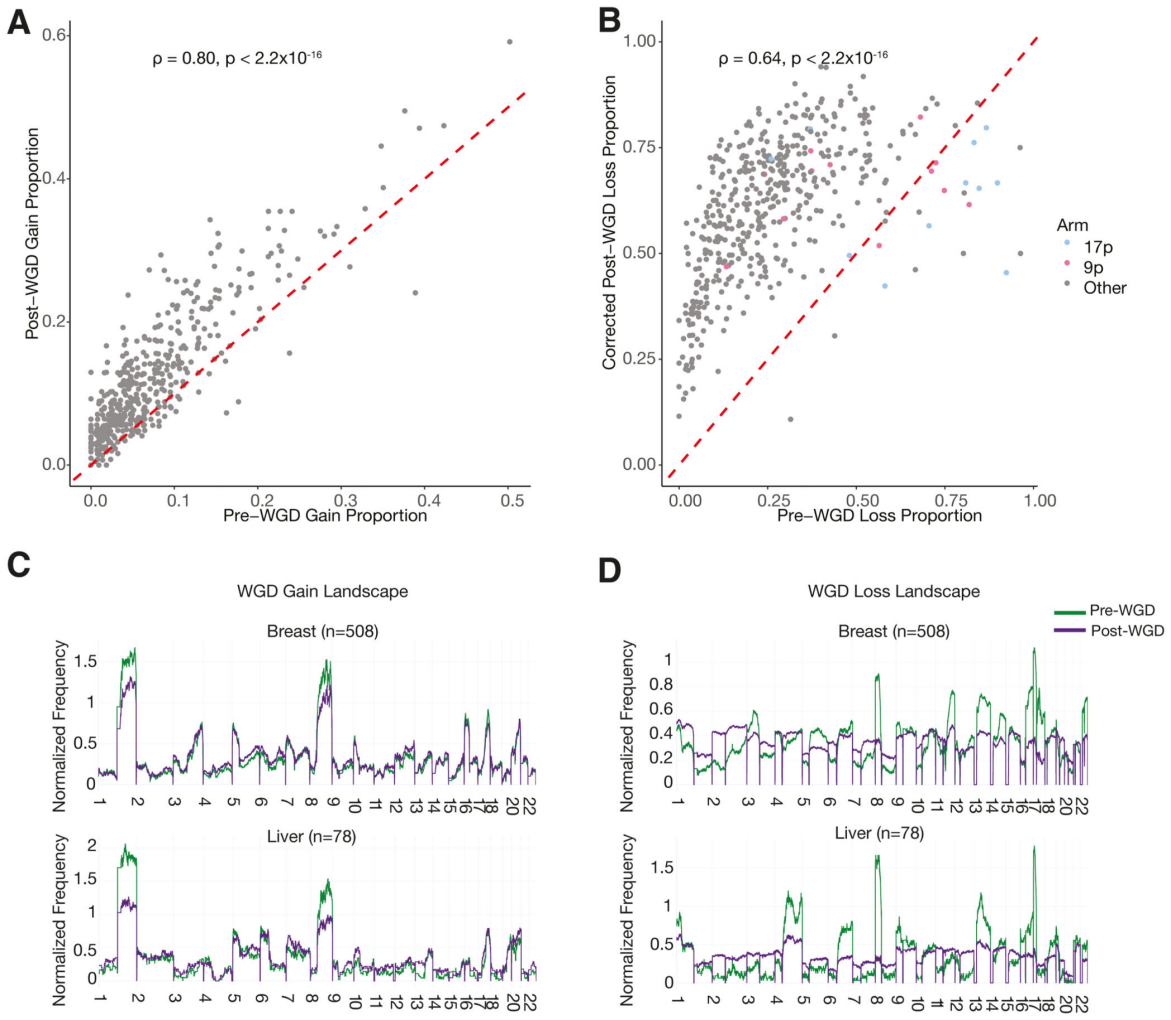
The landscape of pre- and post-WGD copy number events. **A**, Frequency of arm-level gain events occurring before and after a WGD in different cancer types. Each point corresponds to the frequency of arm gain relative to WGD in an individual cancer type. **B**, Frequency of arm-level loss events occurring before and after a WGD in different cancer types. Each point corresponds to the frequency of arm loss relative to WGD in an individual cancer type. The post-WGD arm loss frequency is corrected for mutual exclusivity in measuring pre-WGD and post-WGD arm losses in the same region. **C**, Frequency of pre- and post-WGD gains for breast and liver tumors. **D**, Frequency of pre- and post-WGD losses for breast and liver tumors. The post-WGD loss frequency is corrected for mutual exclusivity. Both frequencies are normalized so that the pre- and post-WGD frequencies integrate to the same arbitrary constant.

The landscape of gains and losses along the genome is similar for WGD and non-WGD tumors across cancer types (9). Therefore, we sought to determine whether the landscape of copy number events differs before *versus* after WGD. First, we compared the relative frequency of arm-level copy number gains across different cancer types pre- and post-WGD and found that they were highly positively correlated (Fig. 4A, S37-S38).

We then investigated how arm loss frequencies were affected by WGD. As a pre-WGD loss and post-WGD loss cannot both be inferred for a given genomic region in the same sample, we applied a correction to the post-WGD loss frequency (Methods). With this correction, we also observed clear positive correlations (p<0.001, Fig. 4B, S39). This suggests that losses without LOH observed post-WGD are mostly derived from a continuation of the same processes that lead to pre-WGD LOH losses. However, there are outliers where the pre-WGD loss frequency is much higher than expected given the corresponding post-WGD loss frequency.

Of the 23 arms across different cancer types that had a higher loss proportion pre-WGD than post-WGD, 14 were 9p (n=5) and 17p (n=9, Fig. 4B). Chromosome arms 9p and 17p contain the frequently hit tumor suppressor genes *CDKN2A* and *TP53*, respectively. Thus, while gains and losses are broadly unaffected by a WGD, we find specific arm-level losses that occur disproportionately pre-WGD. We note that most arms had higher event rates post-WGD than pre-WGD, reflective of increased CIN post-WGD. In agreement with previous findings (10,30), we found that the frequencies of chromosome arm events maintained similar levels of (anti)correlation with the overall tumor suppressor and oncogene density (30) across pre-WGD, post-WGD, and non-WGD copy number events (Fig. S40-S41). Despite certain arms having significantly higher pre-than post-WGD loss, the frequency of both event types showed a similar correlation with driver gene density across chromosome arms. This suggests that the disproportionate pre-WGD loss frequency of certain chromosome arms may be due to specific genes, perhaps in the context of a second inactivating hit, rather than the overall tumor suppressor density.

We then normalized the relative rates of pre- and post-WGD events for both gain and loss across the genome. We found that the rates of pre- and post-WGD gains were similar when aggregated across cancer types (Fig. S42). However, there are notable differences in individual tumor types. For example, gains on 1q and chromosome 8 were disproportionately likely to occur pre-WGD relative to other events (Fig. 4C, S42-S46). Gains on chromosome 3 are often pre-WGD for small-cell lung cancers and upper respiratory tract carcinomas (Fig. S45-S46). Chromosome 7 is commonly gained pre-WGD in glioblastomas, in agreement with previous observations that these events occur very early (17) (Fig. S43).

The differences were much larger pre- *vs*. post-WGD for losses, both at the aggregate level and in individual cancer types (Fig. 4D, S47-S49). For example, we observed high levels of pre-WGD loss of chromosome 18 in colorectal and pancreatic adenocarcinomas and 8p in liver, prostate and colorectal tumors (Fig. 4D, S47-S48). Together, our results suggest a model in which the landscape of copy number gains remains broadly similar post-WGD, although several chromosome arms are predominantly lost pre-WGD. The frequencies of copy number changes in tumors are dictated by a combination of physical factors that affect how often they occur, and the selective impact that they confer (31). As the mechanisms that result in copy number gains and losses are similar, this suggests that the change in loss frequencies post-WGD is driven by changes in selective impact.

## Discussion

GRITIC is a Bayesian framework for genome-wide timing of both simple and complex gains in cancer evolution. It leverages the relationship between alternate read counts, copy number, and purity to determine the sequence and timing of gains. GRITIC theoretically allows the timing of gains from any copy number state, although in practice for computational efficiency and timing accuracy, we focus on segments with no more than 500 possible routes and at least 20 SNVs, respectively.

By applying GRITIC to the PCAWG and Hartwig datasets, we measured the genome-wide timing of gains relative to WGD, describing the effect of WGD on chromosomal instability. We found that late WGDs tended to induce a spike in gain activity, which remained elevated compared with the pre-WGD gain rate. Conversely, early WGDs were preceded by an elevated gain rate, which then decreased post-WGD. This increase in genomic instability likely enables WGD tumors to have greater adaptivity in response to therapy, contributing to their poor prognosis (29). It is worth noting that losses may affect these results, as they can make previously occurring gains unobservable. Similarly, because losses cannot be quantitatively timed, the gain rate over mutation time was not corrected for total genomic content. However, the contrasting patterns observed for tumors with different WGD timing suggest that an increase in CIN post-WGD does not solely result from more chromosomes missegregating at the same rate as pre-WGD.

We found that the landscapes of gains occurring before and after genome duplication were similar, which parallels the similarity in gain landscape between tumors with *vs*. without WGD (9). Therefore, we hypothesize that WGD only has a very moderate impact on the fitness landscape of gains. Although the pre- and post-WGD landscapes are also broadly similar for losses, there are selective pressures to lose certain chromosome arms encoding well-known tumor suppressor genes before WGD, leading to LOH.

In WGD tumors, many copy number segments arise through routes that violate the principle of maximum parsimony, even after this was substantially penalized. It is worth noting that we inferred the most parsimonious routes from single biopsies only. Event histories that appear non-parsimonious, as measured in one biopsy, may be parsimonious when the full heterogeneity of copy number in the tumor is considered. These findings call into question the validity of the maximum parsimony assumption in cancer evolution, particularly in the context of inference from a single biopsy.

GRITIC considers the gains in different segments separately. Future work incorporating structural variant information into timing analyses would enable more integrated evolutionary analyses of copy number changes across the genome (24). Similarly, as our inference is limited by ambiguities in resolving routes from SNV read counts, phasing SNVs either to haplotypes or ideally to specific copies will substantially reduce this ambiguity and allow greater resolution of evolution. Currently, we are unable to time events in tumors with multiple WGDs, which we estimate to be 5.8% of the patients in the PCAWG and Hartwig cohorts. While this is theoretically possible in our framework, more work is required to build an inference pipeline that can link the complex gained states across segments.

We have restricted our analysis to the timing of gains using a mutation-based timescale. Although it preserves the true order of events, it does not have a linear mapping to real-time (Supplementary Methods). By only timing gains using clock-like mutations (32), it is possible to time events in absolute time (17). However, as this greatly reduces the number of mutations per segment, only the largest events such as WGDs can be timed in this manner and thus we are unable to consider the timing of individual copy number gains in real time.

We have considered the timing of loss events when they can be compared to the gain timing results obtained from GRITIC. This is principally by contrasting the landscapes of copy number events pre- and post-WGD. Making additional comparisons is limited as we are unable to quantitatively time clonal copy number losses. Future methods could use structural variants to quantitatively time losses that are linked to copy number gains.

In summary, GRITIC is a novel computational framework for inferring copy number gain evolution from a single bulk whole-genome sequencing experiment. GRITIC can be applied across cohorts to reconstruct more complete evolutionary timelines (17) and to better understand CIN, particularly in relation to WGD.

## Methods

### Data collection

Whole genome sequencing, alignment, mutation calling, and copy number data were obtained from the Pan-Cancer Analysis of Whole Genomes (21) and Hartwig Medical Foundation datasets (33) uniformly processed using the Hartwig Medical foundation pipeline. Only samples with a number of reads per clonal copy of at least 5 were considered, a measure of sequencing coverage corrected for tumor purity (Supplementary Methods). This threshold was found to be sufficient from simulations (Fig. S50).

To obtain clonal copy number profiles, PURPLE copy number outputs were rounded to the nearest integer for each parental allele. SNV clustering information was obtained by using the default settings of DPClust (15). DPClust was run for 2,000 iterations with 1,000 burn-in steps.

The cancer type classifications for each sample were obtained from a unified annotation of the PCAWG and Hartwig cohorts (33). The cancer type information for 143 PCAWG and 775 Hartwig tumors was not available from this set of unified annotations. It was obtained by mapping between the cancer type information for each cohort and the unified annotations (Supplementary Methods). This allowed us to obtain unified cancer type information for all PCAWG tumors and an additional 694 Hartwig tumors. The remaining 81 Hartwig samples were removed from our analysis.

SNVs that were identified as part of kataegis events were identified using the PCAWG kataegis detection pipeline (21). These were filtered from the data because kateagis is a localized hypermutation process that leads to sets of SNVs that violate the assumption of constant relative mutation rates across the genome.

### GRITIC

GRITIC enumerates the binary tree structures that represent all routes to a given gained copy number state for a particular segment, accounting for the presence of up to one WGD. These representations are used to sample the SNV multiplicity proportions that correspond to the range of possible gain and WGD timing for each route. The relative probability of each route and gain timing was calculated using a uniform prior and the likelihood of the SNV read counts for the segment given each sampled multiplicity proportion. GRITIC outputs a posterior distribution over gain timing and routes. A full description of the GRITIC method and its principles is provided in the Supplementary Methods file.

### GRITIC WGD calling

To identify WGD in our cohort, we calculated the cumulative number of base pairs spanned by each clonal major copy number state and identified the major copy number state spanning the highest number of total base pairs, *i.e*. the mode of the major allele. If the mode was one, the sample was identified as non-WGD. If it was two, we calculated the individual timing of all segments with major copy number two. A core principle of GRITIC is that WGD causes simultaneous gain across the genome. Therefore, if at least 60% of the base pairs spanned by the major copy number two segments had posterior gain timing distributions with overlapping 90% credible intervals, then the sample was identified as WGD.

This provided WGD calls consistent with those provided by the datasets using copy number profiles alone (Fig. S51A-B). Notably, the samples with a major copy number mode of two but with less than 60% timing overlap were enriched in lung and skin tumors, which are tumor types known to have late copy number gains (Fig. S51C). Indeed, the timing of the maximum overlap was later in mutation time compared to tumors with greater than 60% overlap in the timing of major copy number two gains (p<0.001, Mann-Whitney U Test, Fig. S51D).

### Penalty on non-parsimony

As discussed in the main text, the model evidence used in the computation of the probability for each route provides a natural penalty against additional independent gains because the evidence is integrated over the extra parameters required to model the timing of these additional gains. However, this provides no penalty for the additional loss events required to make each route consistent. Therefore, we applied a penalty term P to the route posterior probability based on the number of events *n* implied by each route.

$$P=e^{-nl}$$

We tuned the penalty parameter *l* on a representative simulated cohort of simulated tumors to have only parsimonious routes. We set *l* such that the total probability of non-parsimony across the cohort was approximately 5%. As this will depend on the exact setup of the simulation, we did not tune *l* precisely; instead, we found that *l*=2.7 was a reasonable penalty, giving ~5% (5.3%) total non-parsimonious probabilities.

### Measuring performance of gain timing inference using simulated data

We used a probabilistic approach to compare the gain timing inferred from GRITIC with the simulated ground truth. For each simulated segment and route, we sorted all inferred gain timings and all true timings and compared these timings pairwise. Although the number of independent gains differs between copy number states, the total number of gains, including those inferred to arise through WGD, is the same across all routes for a given copy number state.

Only comparisons corresponding to independent gains in the sorted true cohort were collected. The pairwise comparisons for each route were stored in a histogram and each comparison from every route was weighted according to the inferred route probability from GRITIC.

### Measuring non-parsimony probabilities

We assessed non-parsimony across the simulated and PCAWG and Hartwig cohorts by examining the total probability assigned to routes for each gained segment that had more events than the route with the minimum number of events for the segment.

### Comparing the timing of major copy number gains

We sought to compare the relative timing of gains leading to different major copy number states across tumors. We calculated the relative percentile rank of each posterior sample of gain timing across the combined posterior distribution over all segments for a given tumor. The percentile ranks were directly compared between the samples. We computed and compared two percentile rank distributions: one with only the initial gain that leads to each complex state and a second with all gains.

### Calculating the rate of gains relative to WGD

The rate of gain relative to WGD was measured by summing the posterior density multiplied by the segment base pair length across evenly sized bins of *gain timing – WGD timing* across the cohort. The bins each had a size of 0.1 in mutation time and were distributed across the 1-99^th^ percentiles of the *gain timing – WGD timing* distribution. A small offset was applied to the bin start points such that one bin ranged from -0.05 to 0.05.

The binned gain-timing distribution needs to be normalized to account for the distribution of WGD timing in each cohort, as the maximum possible time for a gain to occur before and after the WGD is dependent on the WGD timing. Therefore, we normalized the binned posterior density by dividing it by a second binned distribution that used the same samples and the corresponding WGD timing distribution. However, in this normalizing distribution, the gain timing was uniformly distributed across mutation time, and the gains in each sample were weighted by segment base pair length such that each sample contributed equally. This normalizing distribution therefore represented the change in *gain timing – WGD timing* that would occur purely from differences in WGD timing alone.

### Measuring the proportion of samples with gains post-WGD

We sought to identify the proportion of tumors with post-WGD gains. A tumor was identified as having post-WGD gains if at least 50% of the samples from the posterior gain timing distribution for any segment had at least one post-WGD gain.

As the rate of copy number gains generally increases over tumor development independent of WGD, we sought to compare the proportion of tumors with post-WGD gains to that expected from a control cohort of non-WGD tumors.

Each non-WGD tumor was given a pseudo-WGD timing distribution randomly sampled from WGD tumors of the same cohort. The fraction of non-WGD tumors that had at least one gained region that occurred after their randomly assigned WGD timing distribution was then calculated as a control in the same manner as the WGD cohort. Only cancer types with at least ten WGD and ten non-WGD samples were considered.

### Measuring the average timing proximity between independent gains and WGD

We measured the median difference in timing between the posterior gain timing distribution over all the gained segments and the WGD timing sampled from the WGD timing distribution for the tumor. We repeated this process for 25 samples of the WGD distribution for each tumor and calculated the average median difference in timing. This resulted in a distribution of the average difference between WGD and gain timing for all WGD tumors in our cohort.

This distribution was compared to a control distribution calculated identically, except that all WGD distributions were randomly permuted between WGD tumors of the same cancer type.

### Inferring punctuated gains

We applied a permutation-based approach (34) to identify samples that had pan-genome gains occurring in a punctuated burst using a method similar to that of Gerstung *et al*. (17). For each sample, we compared the gain timing variation across segments to permuted samples with gains obtained from across tumors with the same cancer time. A tumor was defined as punctuated if its gain timing variation was lower than 95% of permuted samples. A full description of the punctuated gains inference method is provided in the Supplementary Methods file.

### Determining the relative order of copy number events and WGD

The relative order of independent copy number gains and WGD was determined from the joint posterior distribution over gain timing and routes. Each posterior sample for a gained segment in a WGD tumor contains the sampled timing of all the independent gains and the sampled WGD timing. The average number of gains that occur pre- and post-WGD for each segment was then computed from the posterior samples.

The assignment of gains pre- and post-WGD is sensitive to parsimony considerations, particularly for gains that occur at a time close to the WGD. This is because each gain pre-WGD is equivalent to two post-WGD gains in terms of how much it increments the copy number and therefore affects the total number of events required for a route. This is why we present all relevant results with and without a penalty on non-parsimony. The overall proportion of gains pre-WGD and post-WGD are highly correlated with and without the penalty (Fig. S52A-B).

A region was identified as having a pre-WGD loss if its minor copy number was zero and a post-WGD loss if its minor copy number was one. This involves a weak assumption of parsimony as a minor copy number of zero could arise from two post-WGD losses, though without any other gains, a minor copy number of one cannot arise from a pre-WGD loss.

To correct for mutual exclusivity when measuring pre- and post-WGD losses, a corrected post-WGD loss proportion was calculated by dividing the post-WGD loss proportion by 1 – the pre-WGD loss proportion, thereby changing the denominator to only just the samples that did not have a pre-WGD loss.

The implied losses from complex gained segments are not included in the analysis of the WGD loss landscapes as they comprise a minority of overall losses and cannot straightforwardly be combined with the proportions corrected for mutually exclusivity in measuring pre- and post-WGD losses of the minor allele.

### Calculating arm pre- and post-WGD event rates

We assessed a chromosome arm as being pre- or post-WGD gained in a WGD tumor if at least 50% of the total base pairs in the arm belonged to segments with at least 50% of posterior gain timing samples and at least one gain pre- or post-WGD, respectively. Similar to the classifications made at a segment level, an arm was classified as having a pre-WGD loss if at least 50% of the arm had a minor copy number of zero and as a post-WGD loss if at least 50% of the arm had a minor copy number of one.

### Pan-genome copy number event landscapes

We also produced pre- and post-WGD gain and loss proportions at 1kb resolution across the genome. For certain cancer types with a low number of samples, the pre-WGD loss proportion was 1.0, leading to division by zero when calculating the corrected post-WGD loss proportion. Therefore, we clipped the pre-WGD loss proportion to a maximum of 0.95 when calculating the corrected post-WGD loss proportion. To compare relative rates, pan-genome event proportions were normalized such that the integral of the proportions over the genome was equal to 10^9^, chosen to provide a normalized event frequency with a magnitude of approximately one.

## Supplementary Material

Supplementary Methods

Supplementary Figures

Supplementary Table 1

## Data Availability

An access request for sequencing data and metadata from the Hartwig Medical Foundation can be found at https://www.hartwigmedicalfoundation.nl/en/data/data-access-request/. Researchers with ICGC access can obtain Hartwig pipeline output for the ICGC subset of the PCAWG cohort by following instructions at https://docs.icgc-argo.org/docs/data-access/icgc-25k-data. Similarly, researchers with TCGA access can obtain Hartwig pipeline output for the TCGA subset of the PCAWG cohort at https://icgc.bionimbus.org/files/5310a3ac-0344-458a-88ce-d55445540120. GRITIC output files for the PCAWG, Hartwig and simulated samples are deposited on Zenodo at https://zenodo.org/records/12010145 (doi: 10.5281/zenodo.12010144).

## Statement of significance

Complex genomic gains are associated with whole-genome duplications, which are frequent across tumors, span a large fraction of their genomes, and are linked to poorer outcomes. GRITIC infers when these gains occur during tumor development, which will help to identify the genetic events that drive tumor evolution.

## Code availability

GRITIC is available at https://github.com/VanLoo-lab/gritic. The scripts used to run GRITIC on PCAWG, Hartwig and simulated samples, as well as to produce the figure for this manuscript can be found at https://github.com/VanLoo-lab/gritic_analysis.
