# Supplementary material for: The history of chromosomal instability in genome doubled tumors
Source: Cancer Discov. Author manuscript; Available in PMC 2024 Sep 19. (PMC7616501; doi:10.1158/2159-8290.CD-23-1249)
Supplement: Supplementary Methods [file EMS197414-supplement-Supplementary_Methods.pdf]

The history of chromosomal instability in genome doubled tumors

Supplementary Methods

Contents

1 Summary **4**
1.1 Principles of mutation time 4

2 Mutation time in practice **6**

3 Limitations and assumptions of mutation time **10**
3.1 Timing in the clonal period 10
3.2 Mutation rates 11
3.3 Indels 11
3.4 The number of SNVs required to time a gain 11
3.5 Infinite sites assumption 12
3.6 Subclonal copy number changes 12

4 Problems with timing more complex gains **13**

5 Representing routes as binary trees **15**

6 Using the representations to time complex gains **19**

7 Multiplicity spaces **22**
7.1 Multiplicities spanned by a 3+0 copy number state 22
7.2 Multiplicities spanned by a 4+0 copy number state 24
7.3 Multiplicity spanned by more complex states 28

8 GRITIC Methods **31**
8.1 Generating trees 31
8.1.1 Generating tree structures 31
8.1.2 Generating WGD nodes 31
8.2 Generating constraint matrices 32
8.3 GRITIC model 33
8.3.1 Modeling variant allele frequencies 33
8.3.2 Likelihood for a single single nucleotide variant (SNV) . . 35
8.3.3 Model Likelihood 35
8.3.4 Parameter priors 36
8.3.5 Power to detect correction 37
8.3.6 Model posterior and marginal likelihoods 38
8.4 Sampling states 38
8.5 whole genome duplication (WGD) timing estimation 39
8.6 Hit and run algorithm 41
8.7 Ensuring sufficient density of samples 43
8.8 Subclonal multiplicity simplification 44
8.9 Counting the number of events 45

9 Other methods **46**
9.1 Cancer type annotation 46
9.2 Sample filtering 47
9.3 Sample simulation 48
9.3.1 Representative cohorts 48
9.3.2 Cohort with uniform gain timing 49
9.3.3 Simulation for setting minimum quality thresholds 50

9.4 Assessing route probability calibration in simulated data 51
9.5 Measuring agreement between route histories 51
9.5.1 Measuring route history agreement within a chromosome 51
9.5.2 Measuring route history agreement across samples . . . 52
9.6 Inferring punctuated gains 52
9.7 Assessing non-parsimony from cell line data 53
9.8 Measuring non-parsimony in clear cell renal cell carcinomas . . 54
9.9 Measuring CIN in single cells 55
9.10 Classifying segments in punctuated gains 56
9.11 Nearest neighbor tests 56

1 Summary

In these supplementary methods, we describe the theoretical basis for Gain Route Identification and Timing In Cancer (GRITIC), our novel method for timing complex copy number gains from SNV data. We first derive the quantitative relationship between SNV multiplicity proportions and the timing of simple copy number gains and discuss a number of assumptions and limitations that underpin mutation time.

Then, we introduce a tree-based representation of copy number event histories and use this to infer the relationship between SNV multiplicity proportions and gain timings for complex copy number states. We next describe how these principles are applied in GRITIC, a new generalized method for timing copy number gains. Finally, we describe the technical details of the GRITIC implementation.

1.1 Principles of mutation time

Using a limited number of assumptions, the timing of clonal copy number gains can be measured relative to the tumor SNV burden. When a copy number gain occurs, SNVs in the region of the gain are duplicated onto the newly gained allele and are therefore present on two copies in the cell. The number of allelic copies that an SNV is present on is known as its multiplicity.

Absent a subsequent copy number loss, SNVs that occur before the gain will have multiplicities greater than one. We can then apply the infinite sites assumption (1), which posits that the genome can be treated as infinite in size and, therefore, any base pair can be mutated at most once in the development of a tumor. Therefore, the only route for a mutation to have multiplicity greater than one is through duplication in a copy number gain.

Using the infinite sites assumption in segments with a copy number gain, all SNVs with multiplicity two on the gained parental allele must have occurred before the gain and all those with multiplicity one must have occurred after (Fig.

M1a). This can be used to infer the timing of the gain relative to the accumulation of SNVs. The greater the number of SNVs with multiplicity two, the later the copy number gain occurred (Fig. M1b). For a given copy number segment, the larger number of parental alleles is defined as the major copy number and the smaller the minor copy number. Under the infinite sites assumption, the multiplicity of an SNV can be no larger than the major copy number of the section of the genome where it is located.

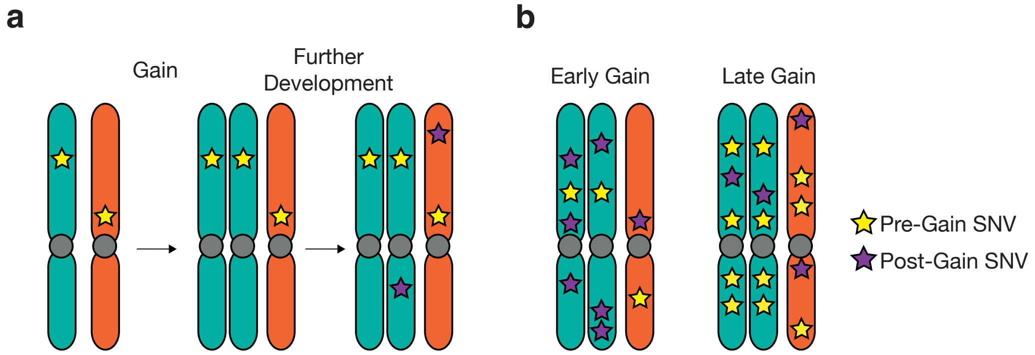

Figure M1: **a.** Schematic of the accumulation of SNVs during tumor development. SNVs on the gained allele are copied over to the new allele. **b.** Illustration of the concept that the earlier a copy number gain occurs, the greater the ratio of SNVs on single copies to multiple copies.

Given the above, gain timing can be assessed quantitatively by calculating the ratio of SNVs with multiplicity two to multiplicity one, after correcting for total genome content and the presence of SNVs on the non-gained allele. This gives the timing of the gain measured in mutation time. If only clonal SNVs are considered, mutation time has useful properties. It can be defined on a scale from zero to one, where zero is conception and one is the emergence of the progenitor cell to all cells in the tumor biopsy, the most recent common ancestor (MRCA).

Mutation time is defined to be linear with respect to SNV accumulation. As the mutation rate of a tumor typically increases over development, mutation time is non-linear with respect to real time. Equal intervals of mutation time

likely correspond to shorter real time periods as the tumor develops (Fig. M2a). Correspondingly, equal intervals in real time correspond to larger periods of mutation time over tumor development (Fig. M2b).

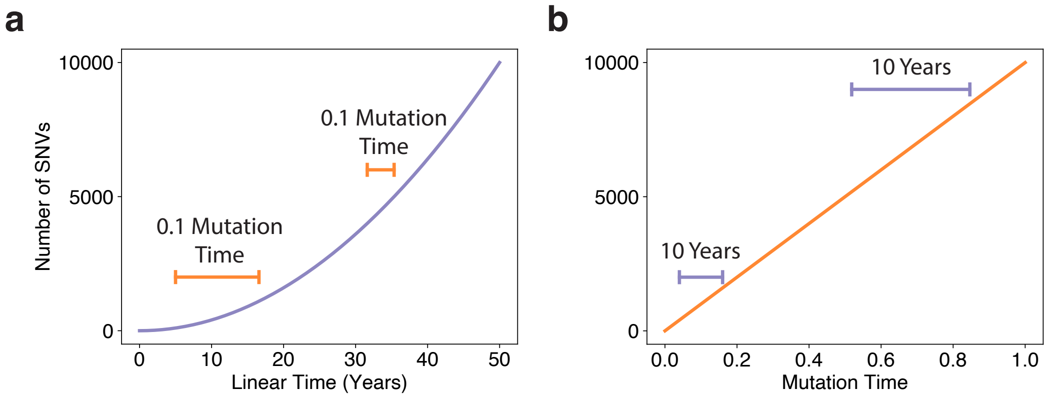

Figure M2: **a**. Schematic of a mutation accumulation in a tumor with a linearly increasing mutation rate. Equal units of mutation time are shown at different points in linear time. **b**. Mutation accumulation in the same tumor in mutation time. Equal units of real time are shown starting at different points in mutation time.

Although non-linear with respect to real time, mutation time is useful because it preserves order. If one gain occurs earlier than another in mutation time, it occurs earlier during tumor development. Mutation time can therefore be applied to build timelines for copy number gains in individual tumors, identifying which gains are the earliest to arise and which only occur when the tumor has already accumulated a number of SNVs and copy number gains.

2 Mutation time in practice

To illustrate how to use SNV multiplicities to time a simple gain, we consider the case of a genomic region with a single copy number gain (Fig. M3). The following derivation follows from the principles first outlined in the initial SNV-based gain timing methods (2, 3, 4, 5).

Any mutations that occur on the gained parental allele in mutation time period

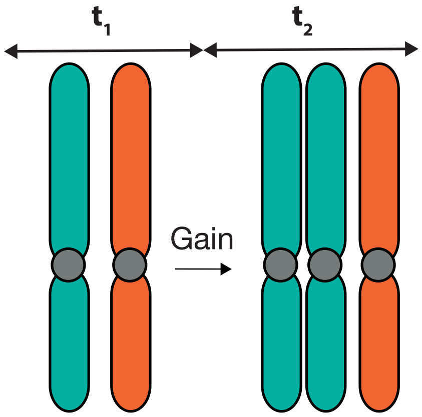

Figure M3: Schematic of a single copy number gain leading to a 2+1 state. The time period t_1 corresponds to the period between conception and the time of the gain and the period t_2 between the gain and the emergence of the most recent common ancestor of the tumor.

t_1 will result in two copies. Those that occur on the non-gained allele at any time and on the gained allele during t_2 will only be present on one copy. We therefore obtain a set of equations for the number of mutations on i copies N_i.

$$\begin{aligned} N_1 &= \sigma(t_1 + 3t_2) \\ N_2 &= \sigma t_1 \end{aligned} \tag{1}$$

Here σ is the mutation rate for the gained segment. σ is a constant as it is defined relative to mutation time. By the definition of mutation time, we also have that $t_1 + t_2 = 1$, and by reformulating the equations to use the proportion of SNVs on i copies $m_i = \frac{N_i}{\sum_j N_j}$, we can solve for the time of the gain t_1.

$$t_1 = \frac{3m_2}{m_1 + 2m_2} \tag{2}$$

Note that the gain timing t_1 is independent of the mutation rate σ. This is a very useful property of mutation time as it allows for gain timing to be compared

within a tumor without directly considering the local mutation rate.

We now examine a second case where the non-gained allele is lost during development (Fig. M4). After considering the number of SNVs with different multiplicities at the final state, we arise at a similar set of equations to (1).

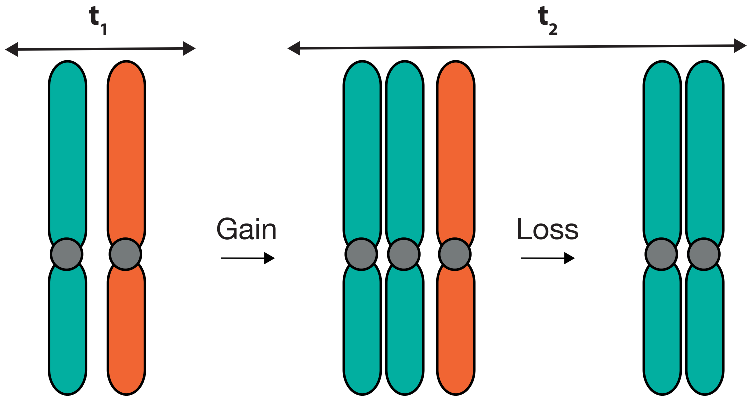

Figure M4: Schematic of a single copy number gain and loss leading to a 2+0 state. The time period t_1 corresponds to the period between conception and the time of the gain and the period t_2 between the gain and the emergence of the most recent common ancestor of the tumor. The loss occurs at an arbitrary time across the clonal period.

$$\begin{aligned} N_1 &= 2\sigma t_2 \\ N_2 &= \sigma t_1 \end{aligned} \tag{3}$$

The SNVs present on the allele that was lost cannot be observed, but this is not necessary to time the gain. The solved equation (4) is identical to the 2+1 case (2) up to a decrease in the multiplicative constant. The reduction in the multiplicative constant corrects for the lack of multiplicity one SNVs that would have occurred from the lost allele. This equation applies regardless of when the loss occurs during the clonal evolutionary period.

$$t_1 = \frac{2m_2}{m_1 + 2m_2} \tag{4}$$

Finally, consider the case of gains leading to a 2+2 copy number state. Similar to before, we have the following multiplicity equations:

$$\begin{aligned} N_1 &= \sigma(2t_2 + 4t_3) \\ N_2 &= \sigma(2t_1 + t_2) \end{aligned} \tag{5}$$

There are now four variables and three equations (including $\sum_i t_i = 1$) and therefore this system of equations is under-determined. It can however, be solved for t_1 and t_2 together.

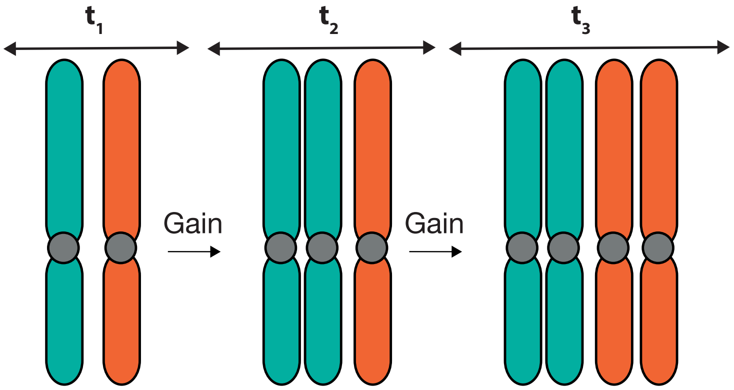

Figure M5: Schematic of the copy number gains leading to a 2+2 state. The time period t_1 corresponds to the period between conception and the time of the first gain, t_2 to the period between the first and second gain and the period t_3 to the period between the second gain and the emergence of the most recent common ancestor of the tumor.

$$t_1 + \frac{t_2}{2} = \frac{2m_2}{m_1 + 2m_2} \tag{6}$$

Further progress can be made if we assume that the two copy number gains are simultaneous, such as in a whole genome duplication (WGD). Then $t_2 = 0$ and t_1 is fully specified. This was the innovation first applied by Gerstung *et al.* to time gains leading to 2+2 states (6). Note that under this condition, equation 6 is identical to the timing equation for a 2+0 gain, equation 4. This follows as both parental alleles in the 2+2 state can each be considered as a separate 2+0 state with identical gain timing.

3 Limitations and assumptions of mutation time

The principles of timing copy number gains outlined in the previous section rely on a number of assumptions and have limitations in order to obtain accurate timing.

3.1 Timing in the clonal period

Mutation time is typically only extended to the clonal evolutionary period. This is because all observed SNVs that occurred during this period were all present in a single cell, the tumor's MRCA. This means that they accumulated sequentially and can therefore be used as a reference timescale.

In the subclonal period of the tumor, this is no longer the case. Different mutations can accumulate in parallel across different subclones, or sequentially in descendant subclones. While parallel SNV accumulation can be modelled in mutation timing frameworks (6), it relies on accurate subclonal phylogenies and SNV assignments to each subclone to obtain the correct accumulation models. As subclonal phylogenies obtained from single sample biopsies are often ambiguous (7), timing from such data is typically restricted to the clonal evolutionary period.

3.2 Mutation rates

The SNV multiplicity ratios are normalized for the overall mutation burden of each segment. This means that a uniform mutation rate across the genome is not necessary to compare the timing of gains across different segments within a single tumor.

Instead, a less stringent assumption is required, that the relative mutation rate remains proportional across all regions during tumor development, at least at the length scales (typically $\approx$ Mb) of the gained segments that are timed. Processes that lead to local hyper-mutation such as kataegis (8) can violate this assumption. However, such mutations typically have distinguishable properties (8, 9) and so can be filtered.

3.3 Indels

Only SNVs are used to define mutation time. Indels are not included, even though they also accumulate over the course of tumor development. This is because the greater degree of alteration caused by indels compared to SNVs causes a systematic alignment bias. Fewer indel-containing reads will correctly align to the reference genome compared to SNVs, causing indel multiplicities to be systematically underestimated. Although computational methods have been developed to correct this alignment bias (10), the simplest approach is to define mutation time using SNVs only.

3.4 The number of SNVs required to time a gain

A sufficient number of SNVs is required to time copy number gains with reasonable precision. This can be problematic for certain cancer types with low mutation rates (5). More generally, it means that whole genome sequencing data is required for timing, as the number of SNVs measured by whole exome sequencing or targeted panels is typically too low to time all but the largest copy

number gains.

3.5 Infinite sites assumption

A recent study tested the validity of the infinite sites assumption across the Pan-Cancer Analysis of Whole Genomes (PCAWG) cohort (11). Although sequential mutations to the same base pair could be identified in a number of tumors, they remain overall very rare in the context of the total SNV burden. The limited violations will cause a slight bias towards measuring later gains, due to the increase in SNVs with multiplicity greater than one.

3.6 Subclonal copy number changes

Subclonal copy number events will lead to SNVs with fractional multiplicity states. Most SNV gain timing methods assume entirely clonal tumor copy number profiles and therefore only measure integer multiplicity states. This can lead to systematic biases in the measurement of gain timing. For example, a subclonal loss on a gained allele would lead to an underestimation in the number of SNVs with multiplicity >1 and therefore the timing measurement to be biased earlier.

It is possible to infer limited subclonal copy number from whole genome sequencing data (12). *MutationTimeR* used this information to correct the effect of subclonal copy number changes on gain timing measurements. However, this requires an assumption of which of the subclonal copy number state was present in the tumor's most recent common ancestor. Nevertheless, an assumption of clonality has been found to be reasonable for most tumor copy number events (7) and so any timing bias resulting from subclonal changes will generally be small. In this work, we assume a single clonal copy number profile for each tumor.

4 Problems with timing more complex gains

The equations used to relate SNV multiplicity proportions to timing are often under-determined. Consider a segment that undergoes three copy number gains to arrive at a 3+2 state (Fig. M6).

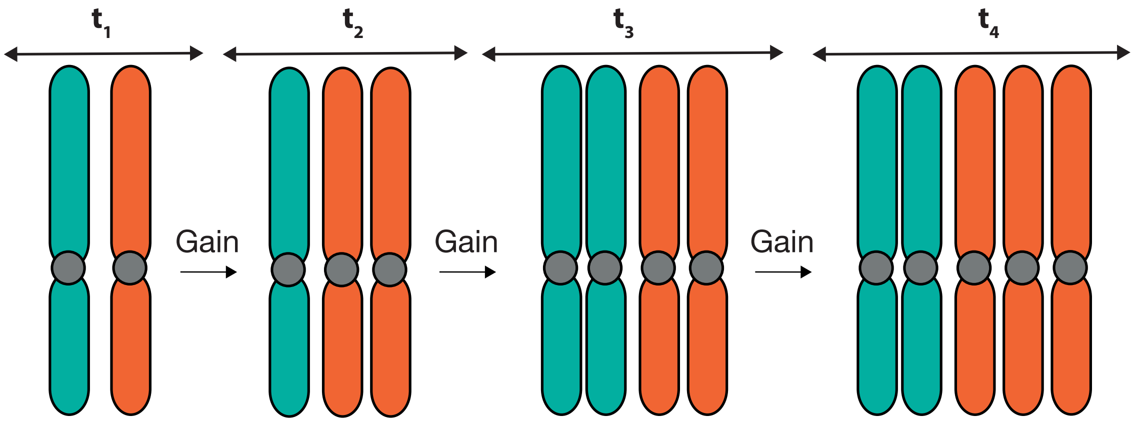

Figure M6: Schematic of a set of copy number gains leading to a 3+2 state.

As before, we can write the equations for the number of mutations with each clonal multiplicity:

$$\begin{aligned} N_1 &= \sigma(t_2 + 3t_3 + 5t_4) \\ N_2 &= \sigma(t_1 + 2t_2 + t_3) \\ N_3 &= \sigma t_1 \end{aligned} \quad (7)$$

Now there are five unknowns and four equations (including $\sum_i t_i = 1$). This means this set of equations is under-determined. However the timing of the first gain can be fully solved (6).

$$t_1 = \frac{5m_3}{m_1 + 2m_2 + 3m_3} \quad (8)$$

If we assume that the first and second gain occur at the same time so $t_2 = 0$, as

could occur during a WGD (Fig. M7a), then the equations become fully determined. This is the assumed route history and equation used by *MutationTimeR* to time the gains in a 3+2 state (6).

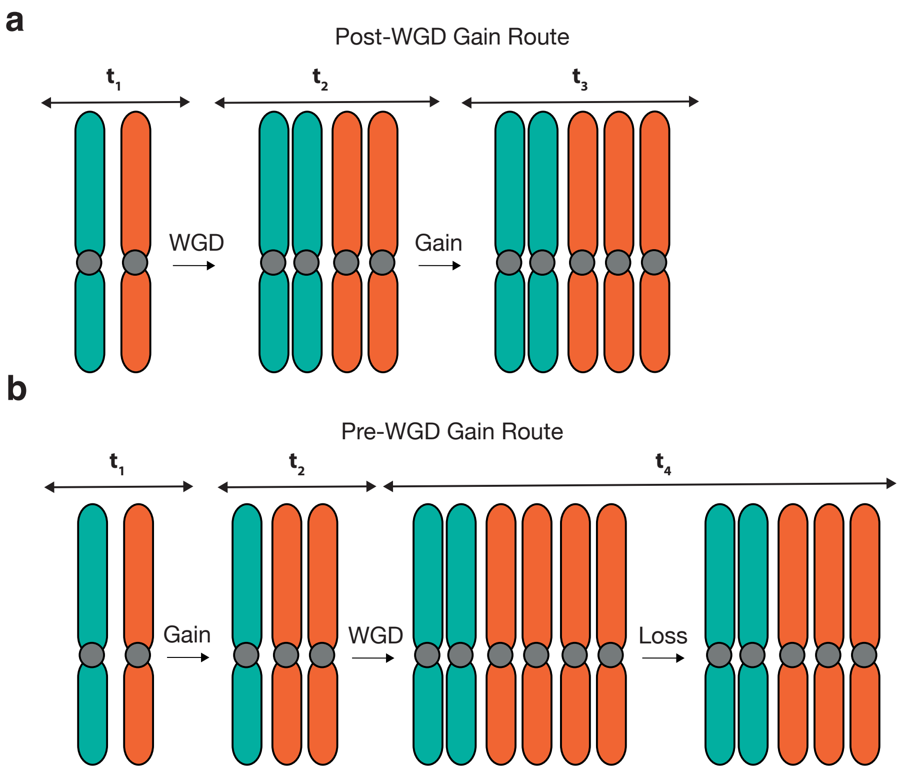

Figure M7: Schematic of two routes in a WGD tumor that lead to a 3+2 state. **a,** The most parsimonious route involving a single gain post-WGD **b,** A less parsimonious route with a pre-WGD gain and post-WGD loss.

$$
\begin{aligned}
t_1 &= \text{WGD Timing} = \frac{5m_3}{m_1 + 2m_2 + 3m_3} \\
t_3 &= \frac{5(m_2 - m_3)}{m_1 + 2m_2 + 3m_3}
\end{aligned}
\tag{9}
$$

This route posits that a single independent gain occurs after the WGD. How-

ever, it is reasonably plausible, with an additional loss event, that the gain could instead occur before the WGD (Fig. M7b) which leads to a separate set of equations for the timing of the independent gain and the WGD.

The routes displayed in Figure M7 are two potential histories that lead to a 3+2 copy number state. The route given in Figure M7a is the most parsimonious, with the fewest events, and so is the one that is often assumed to occur (6, 13). However, the route in Figure M7b is also plausible and only requires a single loss, a common event in WGD tumors. This is not exhaustive, there are other routes that can lead to a 3+2 copy number state. In the next section, we develop a representation of all event histories that can lead to gained copy number states.

5 Representing routes as binary trees

A copy number gain will give rise to two alleles with a shared set of SNVs. This inheritance pattern can be represented in a binary tree structure. Greenman *et al.* (4) was the first to use tree structures to represent the gain history of a given segment. In this study, the structural variant and copy number information were used to jointly estimate a sequence of events that give rise to a given copy number profile. The time between each event was defined as a separate period. A tree was then constructed with a set of nodes for each time period corresponding to each allele. The directed edges between the nodes represented the inheritance patterns between the time periods. A pair of trees was used to represent the history of both parental alleles.

We developed a conceptually similar tree representation that allowed the possible route histories for a given copy number segment to be enumerated. In this representation, all leaf nodes represent the observed alleles and each ancestral node a copy number gain (Fig. M8). As with Greenman *et al.*, each copy number segment is represented by two trees, corresponding to both parental

alleles. Gains that arise from a WGD are represented as a separate node class as they result in simultaneous gains across the tumor and therefore can be distinguished from independent copy number gains.

As this tree representation uses nodes to represent copy number events rather than time periods, each tree structure represents a distinct route history. As it generically represents copy number gain inheritance patterns, all possible route histories can be described using this tree structure. Therefore, this representation can be used to systematically enumerate the possible routes that lead to a given copy number state, without relying on structural variant data as in Greenman *et al.* (4).

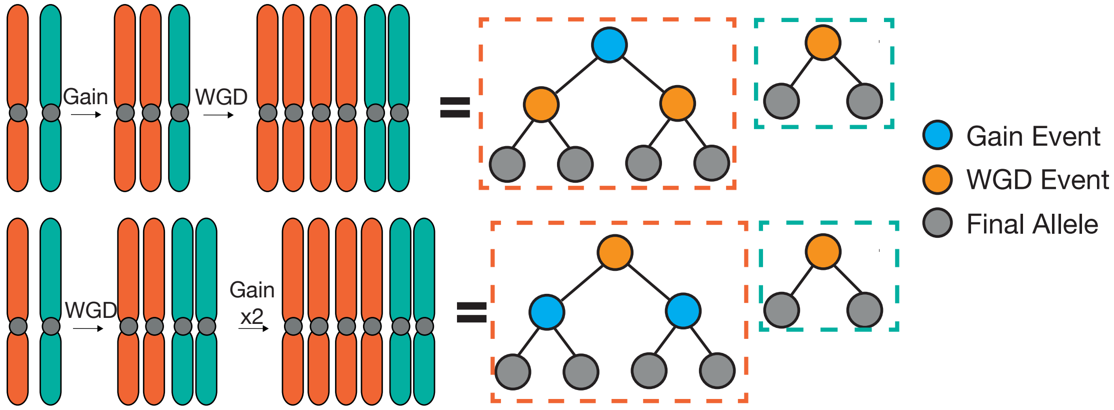

Figure M8: Examples of two routes to a 4+2 copy number state and their corresponding tree representation.

We generate all possible binary trees using a simple recursive approach. We ensure that each tree structure is unique by only generating trees where the number of nodes on the right side of a bifurcation is greater than or equal to those on the left side. After generating all possible tree structures, we then enumerate all possible valid combinations of WGD nodes for the tree structure. Restricting to cases with no more than one WGD, only trees with at most a single WGD node in all paths from the initial to the leaf nodes are considered valid. Figure M9 gives the tree representation of all gain routes leading to a

4+2 copy number state under these constraints. While unlikely, it is possible to have tree representations in WGD tumors that have no WGD nodes. This can occur when all additional alleles gained through the WGD are lost.

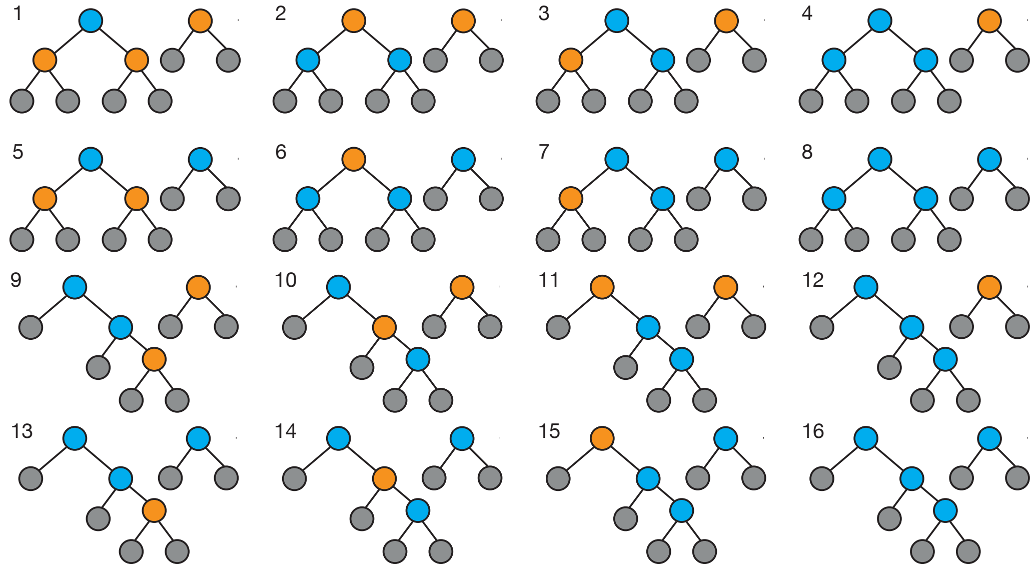

Figure M9: Tree representation of all possible gain routes detectable using SNV multiplicities that lead to a 4+2 copy number state, assuming no more than a single WGD. Losses are omitted from this representation for clarity.

We used the tree representations to enumerate all possible routes across a range of copy number states in WGD tumors. The number of routes rises exponentially with increasing copy number (Fig. M10). As an example, there are 8 possible routes for a 3+2 copy number state and 17,112 possible routes for a 10+4 copy number state.

Certain tree representations imply the occurrence of a copy number loss. In genome-duplicated tumors, trees with leaf nodes that do not inherit from a WGD node must have had a loss event. (Fig. M11). This is because a WGD causes all alleles to be duplicated. Therefore, any allele that does not share an inheritance with at least one other allele through a WGD must have lost the corresponding allele(s) that were gained during the WGD (Fig. M11).

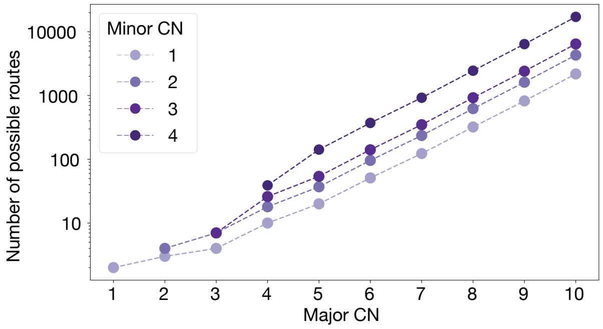

Figure M10: The number of theoretically distinguishable unique routes that can result in different allele-specific states given a single WGD, for different copy number states.

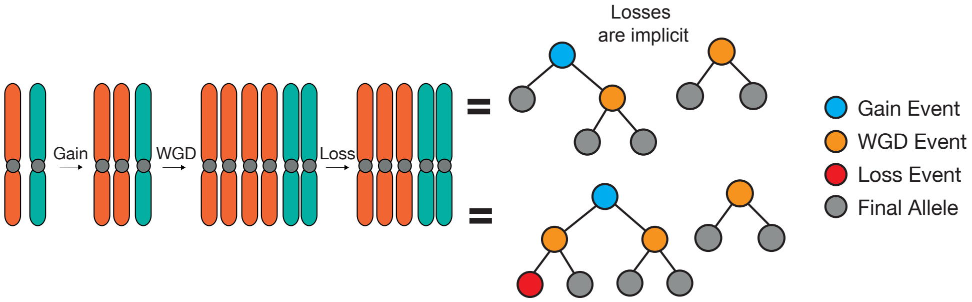

Figure M11: Schematic of a set of copy number events leading to a 3+2 copy number state and the associated tree representation. Although a copy number loss is necessarily implied by the event history, it is omitted for clarity.

By enumerating all possible tree structures, it is possible to identify all possible routes leading to a given copy number state. By possible routes, we refer to all routes that can potentially leave a trace in terms of shared SNVs between alleles. Gains that are subsequently lost cannot be timed. For example, the timing of the gain in a 1+1 state that arises through a gain and then a subsequent loss cannot be measured through this technique (Fig. M12). The only exception to this are the losses of gained alleles that arise through a WGD, as these can be inferred from the presence of a WGD in the tumor as a whole.

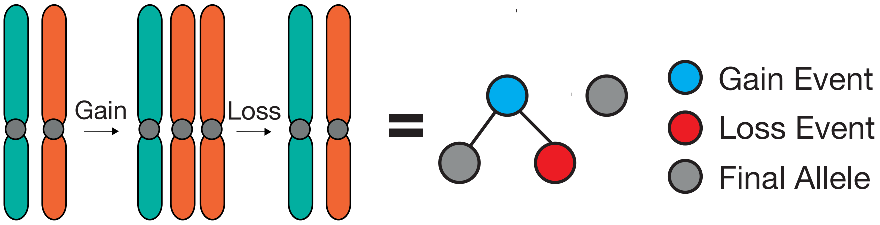

Figure M12: Schematic of a route history and its corresponding tree representation where a 1+1 state gains an allele which is then lost. The gain in this route history cannot be timed, as no record of the gain remains in the SNV data.

6 Using the representations to time complex gains

The tree structures provide a useful basis to time the copy number gains in the routes they represent. They can be straightforwardly applied to infer the relationship between SNV multiplicity and timing similar to equations 1, 3 and 7. As an example, consider the route given in Figure M13. The equations for

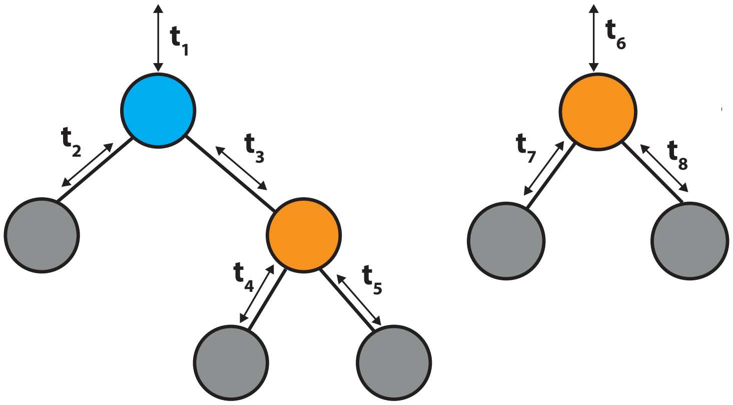

Figure M13: A tree diagram representing a route leading to a 3+2 copy number state, annotated with the time periods between copy number events.

the number of SNVs with each multiplicity state can be written as follows:

$$\begin{aligned} N_1 &= \sigma(t_2 + t_4 + t_5 + t_7 + t_8) \\ N_2 &= \sigma(t_3 + t_6) \\ N_3 &= \sigma t_1 \end{aligned} \tag{10}$$

There are two separate constraints that apply to these multiplicity equations. Due to the definition of mutation time, the sum of the time periods of all paths from each root to the leaves must equal one. Additionally, the WGD is simultaneous, and therefore there is an additional constraint that $t_1 + t_3 = t_6 = t_{WGD}$. Using these constraints and some relabelling of time periods, we can arrive at the same equations as in equation 7.

This can be applied to any copy number tree. The relationship between gain timing and SNV multiplicity and timing as well as the two timing constraints can be represented in two separate binary matrices $\mathbf{A}_M^R$ and $\mathbf{A}_C^R$ defined separately for each route R.

$$\sigma \mathbf{A}_M^R \mathbf{t} = \mathbf{N} \tag{11}$$

$$\mathbf{A}_C^R \mathbf{t} = \mathbf{C} \tag{12}$$

Here, $\mathbf{N}$, $\mathbf{C}$, and $\mathbf{t}$ are the vectors encoding the SNV multiplicities, gain timing constraints and time periods, respectively. Therefore, $\mathbf{A}_M^R$ is the matrix encoding the relationship between the gain timing for the segment and the number of mutations on each multiplicity. $\mathbf{A}_C^R$ encodes the constraints that are applicable to each gain timing. For example, the two matrices for the previous route (Fig.

M13) are given by:

$$\sigma \mathbf{A}_M^R \mathbf{t} = \sigma \begin{pmatrix} 0 & 1 & 0 & 1 & 1 & 0 & 1 & 1 \\ 0 & 0 & 1 & 0 & 0 & 1 & 0 & 0 \\ 1 & 0 & 0 & 0 & 0 & 0 & 0 & 0 \end{pmatrix} \begin{pmatrix} t_1 \\ t_2 \\ t_3 \\ t_4 \\ t_5 \\ t_6 \\ t_7 \\ t_8 \end{pmatrix} = \begin{pmatrix} N_1 \\ N_2 \\ N_3 \end{pmatrix} \tag{13}$$

$$\mathbf{A}_C^R \mathbf{t} = \begin{pmatrix} 1 & 0 & 1 & 1 & 0 & 0 & 0 & 0 \\ 1 & 0 & 1 & 0 & 1 & 0 & 0 & 0 \\ 1 & 1 & 0 & 0 & 0 & 0 & 0 & 0 \\ 0 & 0 & 0 & 0 & 0 & 1 & 1 & 0 \\ 0 & 0 & 0 & 0 & 0 & 1 & 0 & 1 \\ 1 & 0 & 1 & 0 & 0 & 0 & 0 & 0 \\ 0 & 0 & 0 & 0 & 0 & 1 & 0 & 0 \end{pmatrix} \begin{pmatrix} t_1 \\ t_2 \\ t_3 \\ t_4 \\ t_5 \\ t_6 \\ t_7 \\ t_8 \end{pmatrix} = \begin{pmatrix} 1 \\ 1 \\ 1 \\ 1 \\ 1 \\ t_{WGD} \\ t_{WGD} \end{pmatrix} \tag{14}$$

It is not necessary to invert these general matrix equations directly to find a relationship between multiplicity proportions and gain timing for the route. Indeed, if the equation is underdetermined, a direct solution can be somewhat complex to interpret and use. Instead, we can avoid this by using equations 11 and 12 directly.

It is straightforward to use computational methods to find a single arbitrary valid solution $\mathbf{t_S}$ to equation 12. Then, by sampling vectors $\mathbf{t_N}$ from the null space of $\mathbf{A}_C$ such that $\mathbf{A}_C\mathbf{t_N} = \mathbf{0}$ we can sample from the full set of solutions to equation 12 as any $\mathbf{t_S} + \mathbf{t_N}$ will be a solution. This is discussed further in section 8.6.

The timing vector $\mathbf{t}$ represents the time periods between each copy number gain event. The timing of an individual gain can be found by summing the values

of t_i found on the path from the root of the route tree to the node corresponding to the gain in question.

Finally, we can use equation 11 to find the multiplicity proportions that correspond to each sampled timing vector. The likelihood of observing the SNV alternate read counts given the multiplicity proportions can then be used to build a posterior distribution over the gain timing. This is discussed in more detail in section 8.3.

7 Multiplicity spaces

So far, we have outlined a general method to identify all timeable event histories that can give rise to a given complex copy number state, accounting for a potential WGD timing constraint. We then showed how these representations can be used to sample possible gain timing values and associated multiplicity proportions for each route.

We now consider the space of possible multiplicity proportions for different routes across a number of copy number states. By definition, all possible clonal multiplicity proportions for a segment with a major copy number of n_A will exist in the standard simplex of dimension $n_A - 1$. In this section, we show that within the simplex for a copy number state, the subspace of possible multiplicity proportions within the simplex is often distinct for different routes. Therefore, this permits different route histories to be distinguished using SNV information.

7.1 Multiplicities spanned by a 3+0 copy number state

As the simplest example of a complex gained route, consider a 3+0 copy number state. In a WGD tumor, there are three possible route histories (Fig. M14). We arbitrarily label these routes A, B and C. In route A, the independent gain occurs before the WGD, whereas it occurs after the WGD in route B. Finally, route

C has two independent gains, meaning that all extra alleles gained through the WGD have been lost.

All three routes have the same overall tree structure. This structure has a fully determined relationship between SNV multiplicity and gain timing. Therefore, both the first and second gain timing can be uniquely determined from SNV multiplicity. Nevertheless, the WGD constraint restricts the possible multiplicity space. This allows us to make inferences on parsimony in copy number solution evolution. Route B is the most parsimonious solution for this state in a WGD tumor. This is because routes A and C imply additional losses as they contain final alleles that do not inherit from a WGD node.

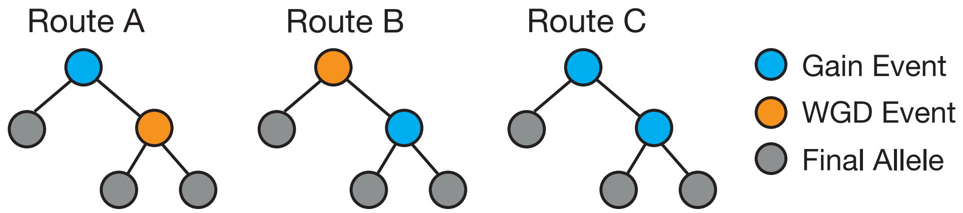

Figure M14: Tree representations of the three possible routes that can lead to a 3+0 state in a WGD tumor.

For a given WGD timing, routes A and B have a single degree of freedom. They therefore have a rank one range of possible multiplicity states. In route A, the independent gain primarily dictates the relative proportion of multiplicity two and three SNVs, whereas in route B it is the relative proportion of multiplicities one and two. We sample the possible multiplicity states using the Markov Chain Monte Carlo (MCMC)-based hit and run algorithm (Section 8.6, Fig. M15).

The set of possible multiplicity proportions for routes A and B change according to the WGD timing. Later genome-duplications increase the potential range of gain timing for route A and thus widen the span of possible multiplicity states. Similarly, the range of possible multiplicity states for route B is reduced with later WGD timing.

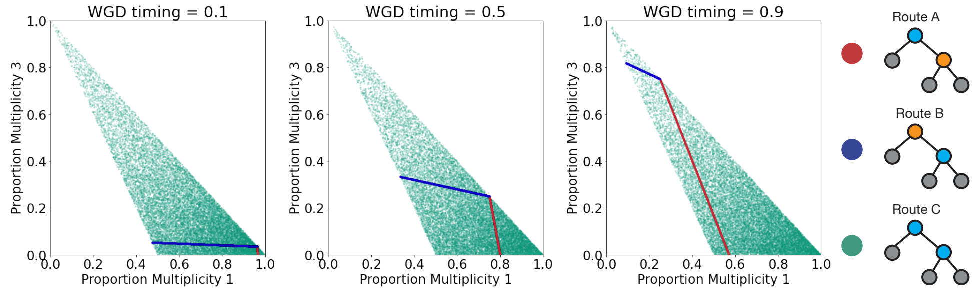

Figure M15: Samples of possible multiplicity states spanned by the three routes that can lead to a 3+0 state in a WGD tumor. Shown for three example WGD timings.

Route C has two independent gains and thus has two degrees of freedom in the multiplicity states. Because neither gain arises from the WGD, the space of possible states is the same regardless of WGD timing. As the timing of either independent gain in route C could be equal to a given WGD timing, the possible multiplicity states for routes A and B are both subsets of route C.

Nevertheless, given a multiplicity state that is consistent with route A or B, it remains more likely that it arose from these routes rather than route C, given that route C would require a loss of the WGD gained alleles as well as an independent gain at the same time as the WGD. This principle of parsimony is evident in increased density of multiplicity samples from routes A and B, if all routes are given an equal number of samples.

7.2 Multiplicities spanned by a 4+0 copy number state

Although the timing of the WGD constraint will vary, there is only one overall possible tree structure for major copy number three gains. This means that when combined across all possible WGD timings, all routes for these major copy number states span the same entire set of multiplicity states.

This is no longer the case for higher major copy number states, as there are multiple possible tree structures. Consider a 4+0 copy number state. In this

case, there are two separate tree structures, regardless of the WGD timing constraint (Fig. M16). The two structures diverge based on the third and final gain. If it occurs on one of the alleles created in the second gain, then the route is called unbalanced, otherwise it is called balanced.

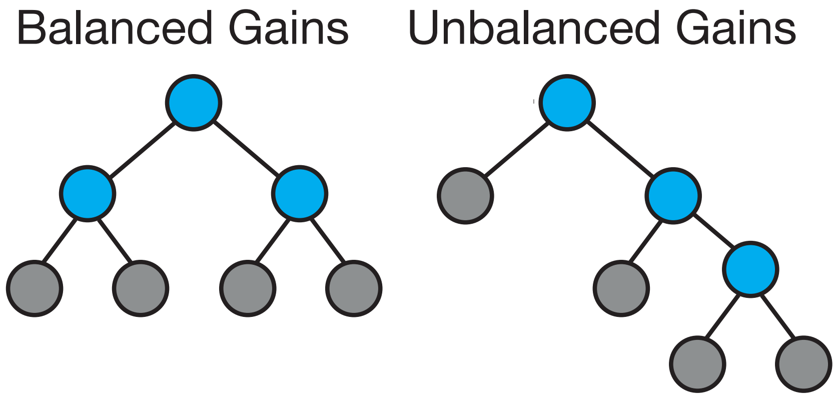

Figure M16: The two possible tree structures for a 4+0 state.

The relationship between multiplicity and gain timing is fully determined for the unbalanced gain structure and under-determined for the balanced gain structure. Without rigorous justification, this can be seen by considering the number of descendants of each gain node. The three gains in the unbalanced case each give rise to a different number of alleles and thus each relates to a different multiplicity state. For the balanced structure, the second and third gains both lead to multiplicity two SNVs and therefore, have under-determined timing. This can be resolved if the two secondary gains are assumed to occur simultaneously.

Therefore, there is ambiguity when timing major copy number four gains, both from the two possible tree structures but also from the under-determined timing of the secondary gains in the balanced gains routes. As discussed earlier, this meant that the timing of major copy number four gains was not fully considered in the three methods used by Gerstung *et al.* (6).

However, the two routes span different possible multiplicity spaces. We there-

fore realized that the routes could be distinguished for a large number of possible gain timings. In the most obvious case, any segment with any number of SNVs with multiplicity three must arise through the unbalanced gains route (Fig. M17). In this case, to visualize the boundaries between the different multiplicity proportions, we systematically enumerate all possible gain timings up to a small precision, rather than sampling using the hit and run approach.

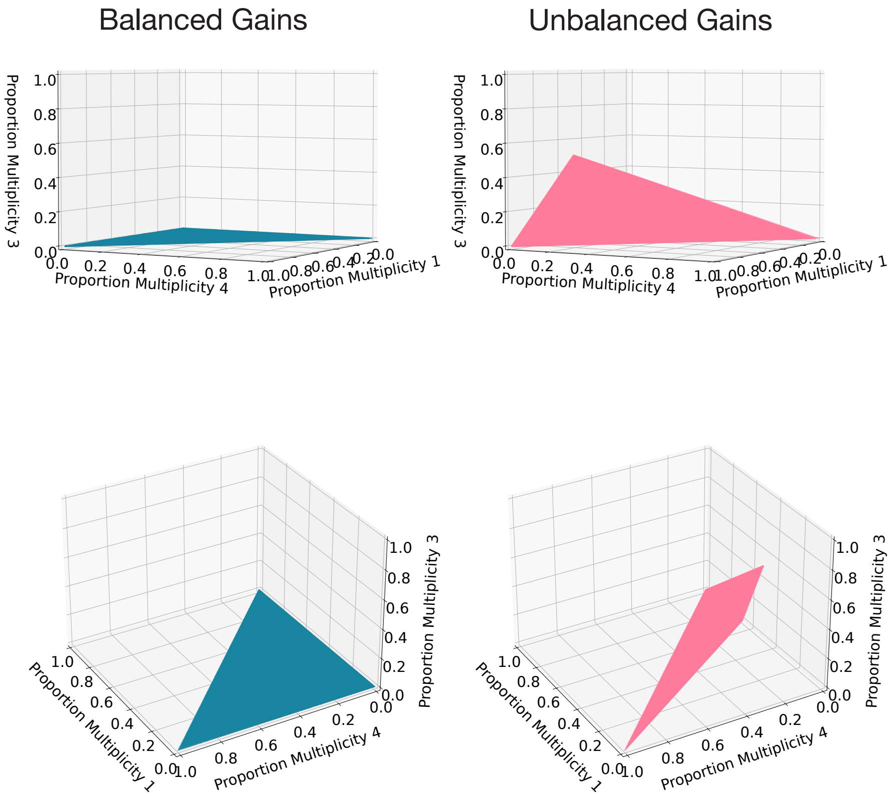

Figure M17: The set of possible multiplicity states corresponding to balanced and unbalanced gains for a 4+0 copy number state.

But even when the unbalanced route is restricted to cases where the second gain immediately follows the first, such that there are no multiplicity three SNVs, there are still differences among the two possible multiplicity spaces (Fig. M18). In these circumstances, the unbalanced gain route only spans a subset of the

full probability simplex across multiplicities one, two and four. In contrast, the balanced gains route spans the entire possible set of multiplicity one, two and four proportions. Therefore, for a large region of this space, SNV multiplicity proportions can be unambiguously assigned to the balanced gain route.

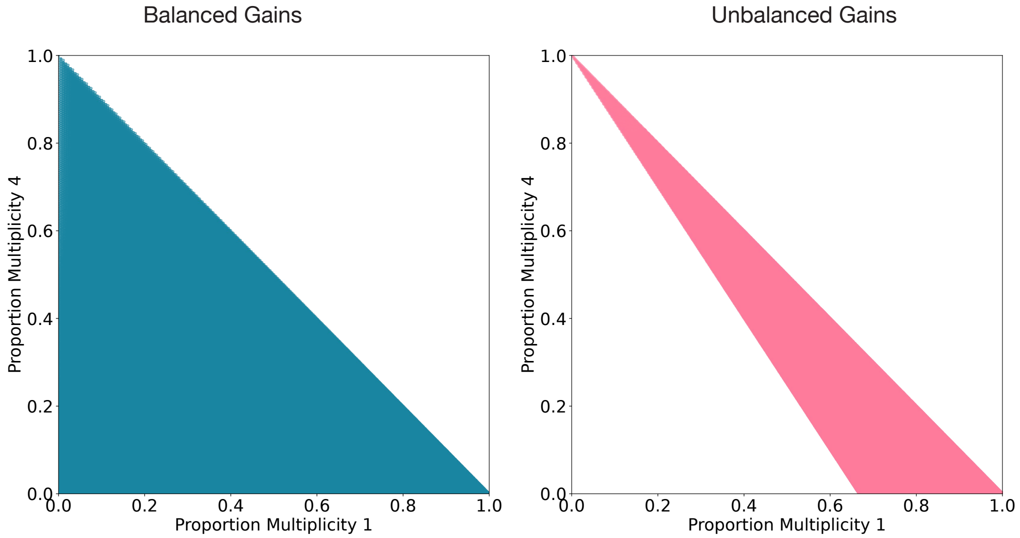

Figure M18: The set of possible multiplicity states corresponding to balanced and unbalanced gains for a 4+0 copy number state. The unbalanced case is restricted to gain timings that lead to no multiplicity three SNVs, to match the space spanned by the balanced case.

This sampling approach also allows for the ambiguity in the timing of the secondary gains in the balanced gain route to be quantified, although not resolved. By collecting samples in a sufficiently local region around a given multiplicity state, the set of possible gain timings that correspond to the multiplicity state can be sampled. This collects any useful timing information about the secondary gains from the multiplicity proportions, even though its precise timing is under-determined. This approach is valid for any under-determined route history.

7.3 Multiplicity spanned by more complex states

For more complex copy number states, the range of possible gain timings and multiplicities span simplexes of higher dimensions and are impossible to directly visualize. As discussed earlier, the number of possible routes also rises exponentially with increasing copy number (Fig. M10).

We display an example of this complexity in Fig. M19. Here, we show the first two principal components of the 5 dimensional space spanned by samples from the possible multiplicity proportions for all 34 routes that lead to a 5+2 copy number state, given an arbitrary WGD timing of 0.4. 90.3% of the total variance can be attributed to these first two components. There is significant overlap between the states, but the constraints for each route mean that different routes have different areas and have density over multiplicity space when sampled with uniform distribution over gain timing.

We then sought to more formally assess whether each route is distinguishable based on their unique multiplicity state spaces and densities, across copy number states. We considered the routes leading to the 20 most common copy number states. We sampled gain timings and associated multiplicity proportions for each route using the hit and run approach (Section 8.6). By calculating the fraction of nearest neighbors for a given multiplicity proportion that arose from the same route (Section 9.11), we could measure how well separated the multiplicity spaces were for each complex copy number state.

We found that an average of 57.8% of nearest neighbors across all routes matched the correct route (Fig. M20). As expected, this varied significantly between copy number states. The number of nearest neighbors with the same route decreases as the number of possible routes increases, from 99.0% for the three routes in the 3+0 state to 21.2% for the 136 routes for 5+4 copy number gains.

Notably, this relationship is not monotonic with regard to the number of routes for a copy number state. For example, the proportion of matching nearest

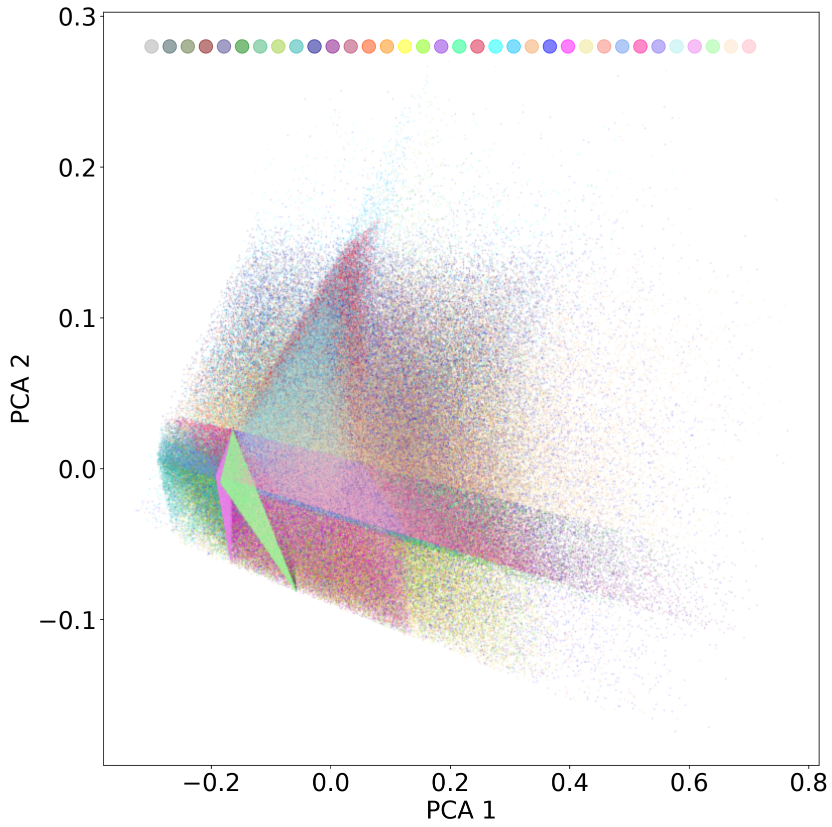

Figure M19: 20,000 samples from the set of possible multiplicity states for all 34 possible routes leading a 5+2 copy number state given a WGD timing of 0.4. Each color represents a distinct route.

neighbors was on average 26.6% across the 226 routes in the 7+2 state compared to 21.2% for the 136 routes leading to 5+4. This can be explained by the minor copy number.

While a higher major copy number increases the number of routes, it also increases the dimensions of the multiplicity space. In contrast, a higher minor copy number still increases the number of routes without expanding the dimension of the multiplicity space. For all states, the proportion of nearest neighbors is significantly higher than would be expected under the random distribution of

multiplicities proportions for each route across the full simplex.

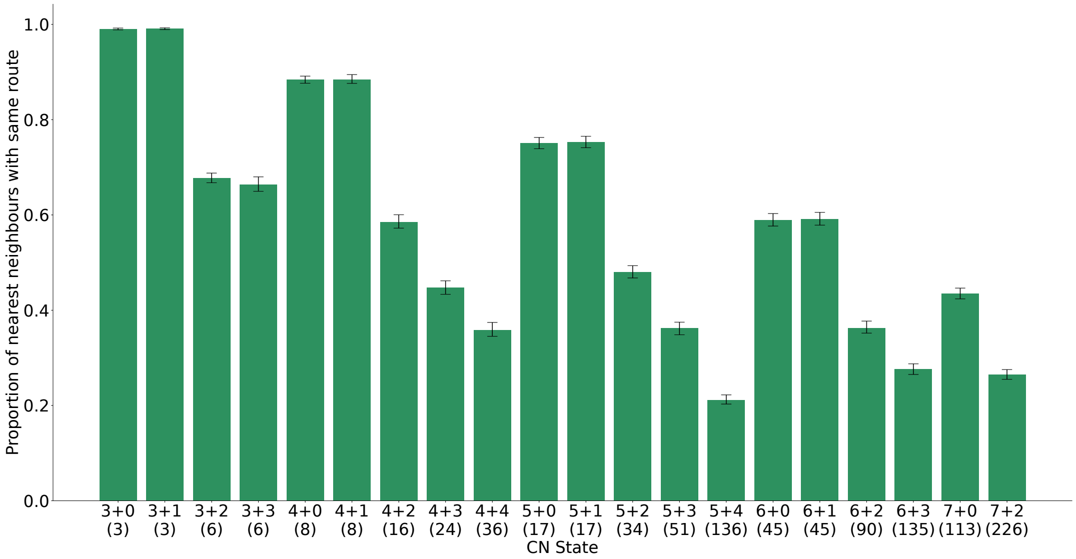

Figure M20: The proportion of nearest neighbors that arise from the same route for a number of complex copy number states. The proportions across a range of WGD timings. The number of possible routes for each state is given in brackets.

It should be emphasized that the separation between the multiplicity states for different routes is partly a result of relative density. Any independent gain can match the timing of a given WGD. Therefore, routes with all independent gains will always be able to match routes with the same tree structure and WGD constraints.

However, as each independent gain represents a new degree of freedom, only a small proportion of samples will have independent gains that match the WGD timing. This can be thought of as a soft parsimony constraint. If a given multiplicity proportion can be formed through a route with WGD constraints, the local density of multiplicity proportions from such a route will be much higher than from the corresponding route with all independent gains.

8 GRITIC Methods

8.1 Generating trees

8.1.1 Generating tree structures

The binary tree structures are generated with a simple recursive approach. GRITIC route representations are unoriented so there is no meaning to left or right side of a bifurcation. Therefore, to ensure that each distinguishable gain route corresponds to a single potential tree, only trees with at least half the leaves on the right side of a bifurcation are generated. One tree is generated for the major allele and a second from the minor allele.

This process generates all possible tree structures for a given copy number state.

8.1.2 Generating WGD nodes

Once the tree structures are identified, we then identify distinguishable set of internal nodes that could correspond to WGD events. Each distinguishable set of WGD internal nodes for a tree, including the empty set, corresponds to a unique gain route R.

We use a brute-force approach to generate all possible combinations of WGD nodes for each given tree structure. All possible sets of any size of the tree's internal nodes are checked to see if it is a valid set of WGD nodes. The only requirement for a set of WGD nodes to be valid is that no WGD node inherits from another. This is because we assume a maximum of a single WGD for any sample we consider.

We then filter this collection of valid sets of WGD nodes to remove equivalent combinations (Fig. M21). For each valid set of WGD nodes for each tree structure, we compute the combined hash of the tree structure and WGD node status using the Weisfeiler Lehman graph hash method (14) as implemented in

`networkx` (15). As each tree with the same hash has an equivalent structure and WGD node status, we only select one tree with each hash. These route trees could likely be much more efficiently generated with a recursive algorithm instead of brute force, but we find that this approach works sufficiently quickly for the tree structures that we consider.

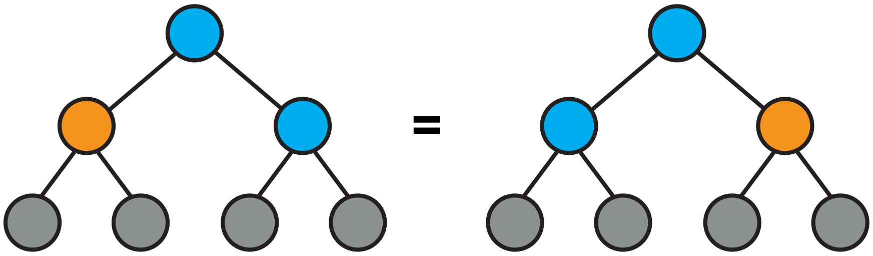

Figure M21: Schematic of two route trees that have an equivalent WGD node structure.

8.2 Generating constraint matrices

Each route tree corresponding to a route R is used to generate two binary matrices that are used to sample timings and multiplicities. As discussed in the main text, the first matrix, $\mathbf{A}_M^R$ encodes the relationship between the timing of each gain and the overall number of mutations. It is a $n_A \times D$ matrix, where n_A is the major copy number of the corresponding copy number state and D is the number of nodes across both tree alleles.

$(A_M^R)_{ij}$ is defined as 1 if node j has i leaf descendants, and 0 otherwise. The leaf nodes themselves are defined as having a single leaf descendant. With this definition we can write equation 11.

$$\sigma \mathbf{A}_M^R \mathbf{t} = \mathbf{N}$$

As described previously, σ is the mutation rate for this segment, $\mathbf{t}$ the vector encoding the gain timing of the nodes and $\mathbf{N}$ is the number of SNVs with each

clonal multiplicity. This equation can be used to provide a mapping between $\mathbf{t}$ and the proportion of SNVs with each clonal multiplicity state $\mathbf{m^c}$.

$$\mathbf{m^c} = \frac{\mathbf{A}_M^R \mathbf{t}}{\sum \mathbf{A}_M^R \mathbf{t}} \tag{15}$$

The second matrix $\mathbf{A}_C^R$ encodes the constraints placed on $\mathbf{t}$. These constraints are that the sum of all t_i on all paths from root to leaf nodes must be 1, given the definition of mutation time. For WGD routes, there is a second constraint that the sum of all t_i all paths from root to WGD nodes must equal some t_{WGD}. $\mathbf{A}_C^R$ is a $P \times D$ matrix, where P is the number of constraint paths for the route. The constraints are encoded in equation 12:

$$\mathbf{A}_C^R \mathbf{t} = \mathbf{C}$$

$(A_C^R)_{ij}$ is defined as 1 if node j is part of the path for constraint i, and 0 otherwise. The constraints C_i are defined as 1 if constraint i is a root-leaf constraint and t_{WGD} if it is a WGD constraint.

8.3 GRITIC model

The core principle behind GRITIC is the identification of the timing of copy number gains from the proportion of SNVs with different multiplicity states in each gained segment. We model these proportions by applying a binomial mixture model to the alterative and reference read counts for the SNV read counts for a given segment.

8.3.1 Modeling variant allele frequencies

The proportion of alternative reads at a given SNV locus is defined as the variant allele frequency (VAF) for each SNV. It is directly proportional to the multiplicity of the SNV. The expected VAF for a mutation with multiplicity x in an

autosomal chromosome can be written as:

$$VAF(x, f, n_T, \rho) = \frac{\rho x f}{n_T \rho + 2(1-\rho)} \tag{16}$$

Here ρ is defined as the proportion of tumor cells in the biopsy, known as the purity. f is the proportion of tumor cells that contain the SNV, the cancer cell fraction (CCF). f is 1 for clonal SNVs and $<$ 1 for subclonal SNVs. The total tumor copy number for the segment is given by n_T. The $2(1-\rho)$ term in the denominator arises as non-cancerous cells have two copies of each autosomal region. For the X chromosome in samples with XY sex chromosomes, this term is changed to $(1-\rho)$. The Y chromosome is not considered for timing in GRITIC.

The equation uses the constant mutation multiplicity assumption (16), where it is assumed that each SNV has the same multiplicity in all of the cells that contain the SNV. With the infinite sites assumption, an SNV can have a maximum multiplicity of the major copy number n_A of the segment.

We further assume that all subclonal SNVs have a multiplicity of one. With this assumption, we can make a simplification. We redefine x to be equal to the product of the multiplicity state and the CCF of the SNV f. Under this redefinition, x is unchanged for clonal SNVs as f is 1. For subclonal SNVs x is equal to f and therefore each subclonal cluster is considered as a distinct multiplicity state. Then, we can simplify the VAF as:

$$VAF(x, n_T, \rho) = \frac{\rho x}{n_T \rho + 2(1-\rho)} \tag{17}$$

For a sample with S subclonal clusters $s_1, s_2, ..., s_S$ and a genomic region with major copy number n_A, the possible multiplicities can be stored in a vector $\mathbf{x} = [s_1, s_2, .., s_S, 1, ..., n_A]$.

8.3.2 Likelihood for a single SNV

The number of SNV alternative read counts is commonly modelled with a binomial distribution, both in SNV subclonal clustering methods such as *DPClust* (12, 17) as well as other methods that use SNVs to time copy number gains, *CancerTiming* (3) and *PhylogicNDT* (13). Other approaches use beta-binomial distributions to model read count overdispersion both for SNV clustering such as in *PyClone* (18), as well as in the gain timing method *MutationTimeR* (6).

While it is clear that overdispersion is required to model bisulfite, exome or single-cell RNA sequencing, it is not obvious that it is a better model for high-coverage whole genome sequencing (7). Indeed, the latest version of *MutationTimeR* (6) has the default overdispersion parameter for the beta-binomial set to zero[1]. Therefore, GRITIC uses a binomial distribution to model the number of alternative reads at an SNV locus.

The likelihood of observing r_A alternative read counts for a SNV given that the multiplicity M of the SNV is x_i, and it has total read counts r_T, all possible multiplicity states $\mathbf{x}$, tumor purity ρ and total copy number n_T is given by:

$$\begin{aligned} P(r_A|M=x_i,\mathbf{x},r_T,n_A,n_T,\rho) &= \frac{Bin_{PMF}(r_A;r_T,VAF(x_i,n_T,\rho))}{\sum_j Bin_{PMF}(r_A;r_T,VAF(x_j,n_T,\rho))} \\ &= \frac{\binom{r_T}{r_A}(\frac{x_i\rho}{\rho n_T+2(1-\rho)})^{r_A}(1-\frac{x_i\rho}{\rho n_T+2(1-\rho)})^{r_T-r_A}}{\sum_j \binom{r_T}{r_A}(\frac{x_j\rho}{\rho n_T+2(1-\rho)})^{r_A}(1-\frac{x_j\rho}{\rho n_T+2(1-\rho)})^{r_T-r_A}} \end{aligned} \quad (18)$$

8.3.3 Model Likelihood

We next seek to evaluate the likelihood of observing the read counts across all j SNVs in the gained segment given the overall proportion of SNVs with each multiplicity.

[1] `https://github.com/gerstung-lab/MutationTimeR` accessed 5th February 2024.

This proportion is encoded in a vector $\mathbf{m} = [m_{s_1}, m_{s_2}, ..., m_{s_S}, m_1, ..., m_{n_A}]$, that gives the proportion of SNVs with each multiplicity x_i in $\mathbf{x}$.

The likelihood of observing all the alternate SNV read counts $r_{A,j}$ given a multiplicity proportion $\mathbf{m}$ for a segment is given by a binomial mixture model over all j SNVs and all i possible multiplicities in $\mathbf{x}$. For brevity, the values $\mathbf{x}, r_{T,j}, n_A, n_T, \rho$ are represented by θ_j.

$$P(\mathbf{r_A}|\mathbf{m}, \boldsymbol{\theta}) = \prod_j \sum_i m_i P(r_{A,j}|M_j = x_i, \theta_j) \tag{19}$$

However, it is not $\mathbf{m}$ that we are directly interested in, but rather the corresponding gain timing vector $\mathbf{t}$. We therefore sought to use the likelihood in equation 19 to construct a posterior distribution for $\mathbf{t}$ given the SNV read counts. The proportion of mutations with each clonal multiplicity, $\mathbf{m^c}$, can be obtained from equation 15.

If there are no subclonal multiplicity peaks, then $\mathbf{m^c} = \mathbf{m}$ but otherwise the proportion of SNVs with each subclonal peak needs to be incorporated to use the likelihood in equation 19.

To achieve this, we define a new vector $\mathbf{c}$ that denotes the proportion of mutations in each cluster. c_0 denotes the proportion of SNVs in the clonal cluster, and $c_1, ..., c_S$ give the proportion of SNVs in each of the S subclonal clusters as determined by a subclonal deconvolution algorithm. Therefore, for arbitrary S:

$$\mathbf{m}(\mathbf{t}, \mathbf{c}, \mathbf{A}_M^R) = [c_1, ..., c_S, c_0 m_1^c, ..., c_0 m_{n_A}^c] \tag{20}$$

8.3.4 Parameter priors

We chose a uniform prior for $\mathbf{t}$ over the set of values of $\mathbf{t}$ that satisfy the constraint equation 12 for the route R : $\mathbf{A_C^R t} = \mathbf{C}$. Therefore, with the volume occupied by the set of valid states denoted by V_R, the prior for $\mathbf{t}$ given a route

R is written as:

$$p(\mathbf{t}|R) = \frac{1}{V_R} \tag{21}$$

This prior term encodes the principle of parsimony, with more constrained routes having a higher prior probability over the space of valid states. We also use a uniform prior $P(R)$ over each of the routes for a given segment. As the number of routes for a given copy number state is fixed, $P(R)$ is just a multiplicative constant and so can be neglected.

Finally, $\mathbf{c}$ is given a weak Dirichlet prior $p(\mathbf{c}) = Dir(\boldsymbol{\alpha})$ where $\boldsymbol{\alpha}$ is defined as:

$$\alpha_i = \frac{\omega_i}{\sum_{j=0}^{S} \omega_j} + 1 \tag{22}$$

Here $\boldsymbol{\omega}$ is the vector of the number of SNVs in each of the clonal and S subclonal clusters across the entire tumor sample, as determined by a subclonal deconvolution algorithm.

8.3.5 Power to detect correction

The higher the multiplicity of a given SNV, the greater the fraction of mutated reads and thus the higher the likelihood of the SNV being detected. This can lead to an overestimate of the proportion of SNVs with high multiplicities. This bias will be particularly apparent in tumors with low purity or depth of sequencing. We therefore applied a heuristic approach to correct for differences in the power to detect each multiplicity state.

We approximate the conditions where a mutation would be detected by assuming that an SNV will only be detected if and only if three mutant reads are detected. For each clonal and subclonal multiplicity state $x_i \in \mathbf{x}$, we estimate the proportion of SNVs, π_i that would be missed by this threshold.

$$\pi_i = \frac{1}{N}\sum_{j=1}^{N}(1-P(r_{A,i} \leq 2|n_{T,i}, M)) = \frac{1}{N}\sum_{j=1}^{N}\sum_{k=0}^{2} Bin_{PMF}(k; r_{T,j}, VAF(x_i, n_{T,j}, \rho)) \tag{23}$$

Then, for each multiplicity proportion $\mathbf{m}$ corresponding to a set of gain timings, we calculate the corrected proportion $\mathbf{m}'$ that would be expected to be observed given the power to detect.

$$m'_i(\mathbf{t}, \mathbf{c}, \mathbf{A}_M^R) = \frac{\pi_i m_i(\mathbf{t}, \mathbf{c}, \mathbf{A}_M^R)}{\sum_j \pi_j m_j(\mathbf{t}, \mathbf{c}, \mathbf{A}_M^R)} \tag{24}$$

8.3.6 Model posterior and marginal likelihoods

The posterior probability of gain timing $\mathbf{t}$, clone share vector $\mathbf{c}$ and route R given the data is written as:

$$\begin{aligned} P(\mathbf{t}, \mathbf{c}, R|\mathbf{r_A}, \boldsymbol{\theta}) &= \frac{P(\mathbf{t}|R)P(\mathbf{c})P(R)P(\mathbf{r_A}|\boldsymbol{\theta}, \mathbf{t}, \mathbf{c}, R)}{P(\mathbf{r_A}, \boldsymbol{\theta})} \\ &= \frac{\frac{1}{V_R} Dir_{PMF}(\mathbf{c}; \boldsymbol{\alpha}) \prod_j \sum_i m'_i(\mathbf{t}, \mathbf{c}, \mathbf{A}_M^R) P(r_{A,j}|M_j = x_i, \theta_j)}{P(\mathbf{r_A}, \boldsymbol{\theta})} \end{aligned} \tag{25}$$

The marginal likelihood for the route R is given by

$$P(R|\mathbf{r_A}, \boldsymbol{\theta}) = \int_{\mathbf{t}}\int_{\mathbf{c}} \frac{\frac{1}{V_R} Dir_{PMF}(\mathbf{c}; \boldsymbol{\alpha}) \prod_j \sum_i m'_i(\mathbf{t}, \mathbf{c}, \mathbf{A}_M^R) P(r_{A,j}|M_j = x_i, \theta_j)}{P(\mathbf{r_A}, \boldsymbol{\theta})} d\mathbf{c} d\mathbf{t} \tag{26}$$

8.4 Sampling states

In practice, we do not directly compute the full integrals described in the previous section. Instead for each route R, we directly take M_R samples from the

prior over $\mathbf{t}$ using the hit and run algorithm to give a set of valid timing vectors $\mathbf{T}$, as described in section 8.6. These are combined with M_R independent samples for the Dirichlet prior for $\mathbf{c}$ to give a set of clonal share vectors $\mathbf{C}$, which with equation 20 can be used to produce a set of multiplicity proportion vectors.

We then use Monte Carlo techniques to obtain posterior distributions for our parameters of interest. As $\mathbf{T}$ and $\mathbf{C}$ are directly sampled from the prior, only an evaluation of the likelihood is required.

The marginal likelihood for route R in equation 26 is approximated as:

$$P(R|\mathbf{r_A}, \boldsymbol{\theta}) \approx \frac{\frac{V_R}{M_R} \sum_k^{M_R} \frac{1}{V_R} \prod_j \sum_i m_i'(\mathbf{T}_k, \mathbf{C}_k, \mathbf{A}_M^R) P(r_{A,j}|M_j = x_i, \theta_j)}{\sum_{v \in \mathbf{R}} \frac{V_v}{M_v} \sum_k^{M_v} \frac{1}{V_v} \prod_j \sum_i m_i'(\mathbf{T}_k, \mathbf{C}_k, \mathbf{A}_\mathbf{M}^\mathbf{v}) P(r_{A,j}|M_j = x_i, \theta_j)} \quad (27)$$

Here in the denominator we are summing over each route v for the full set of valid routes $\mathbf{R}$ for the relevant copy number state.

The posterior distribution of gain timing $\mathbf{T}_l$ and clonal share $\mathbf{C}_l$ given a route R is then written as :

$$P(\mathbf{T}_l, \mathbf{C}_l|R, \mathbf{r_A}, \boldsymbol{\theta}) \approx \frac{\prod_j \sum_i m_i'(\mathbf{T}_l, \mathbf{C}_l, \mathbf{A}_M^R) P(r_{A,j}|M_j = x_i, \theta_j)}{\sum_k^{M_R} \prod_j \sum_i m_i'(\mathbf{T}_k, \mathbf{C}_k, \mathbf{A}_\mathbf{M}^\mathbf{v}) P(r_{A,j}|M_j = x_i, \theta_j)} \quad (28)$$

8.5 WGD timing estimation

In WGD tumors, GRITIC uses the simultaneous occurrence of the duplication as a constraint to improve the timing of gains in complex states. The WGD is timed using a multistage procedure, leveraging information from across the genome. Only samples with a modal major copy number of two have a WGD timing estimation, as described in the main methods.

In a WGD sample, the vast majority of segments with major copy number two are assumed to have been gained during the WGD. Therefore, these are the

segments that are used to time the WGD. However, some segments may violate the proportionality assumption of mutation time (section 3.2), which could bias our estimate of the WGD timing. Therefore we first independently obtain the posterior gain timing for each segment with major copy number two, to identify those with those with consistent timing. The assumption that the two gains that lead to 2+2 segments occur simultaneously is enforced by treating such segments as 2+0, as described in section 2.

Then 90% credible intervals are computed for each major copy number two segment. The point in mutation time that intersects the gain timing credible intervals of the greatest total base pair length of major copy number two segments is calculated. As described in the main methods, if less than 60% of the base pairs spanned by the timeable major copy number two segments had posterior gain timing distributions with overlapping 90% credible intervals, then the sample was excluded (section 9.2). This is because it was likely WGD but violated the principle of simultaneous duplication as measured by mutation time.

The segments with gain timing that intersect this point are assumed to have arisen during the WGD. Therefore to time the WGD itself, these segments are combined and the posterior timing computed again using equation 28. Three posterior distributions are obtained, one for each of the 2+0, 2+1 and 2+2 combined segments.

As we are assuming the prior for t_{WGD} is uniform, and that the data from the segments from different copy number states are independent, the full posterior distribution for t_{WGD} is then proportional to the product of the three posterior distributions for the different copy number states.

As the WGD timing is estimated by combining information across major copy number two segments, we apply a less stringent criteria for the minimum number of SNVs per segment. All major copy number two segments with at least 10 SNVs are timed in WGD samples, instead of the limit of 20 SNVs for all other

segments.

$$P(t_{WGD}|\boldsymbol{\theta}_{2+1}, \boldsymbol{\theta}_{2+0}, \boldsymbol{\theta}_{2+2}) \propto P(t_{WGD}|\boldsymbol{\theta}_{2+1})P(t_{WGD}|\boldsymbol{\theta}_{2+0})P(t_{WGD}|\boldsymbol{\theta}_{2+2}) \quad (29)$$

Here $\boldsymbol{\theta}$ represents all relevant data for the merged segments with the given copy number state. In practice, a discrete WGD posterior for each copy number state is estimated by binning the likelihood weighted timing samples $\mathbf{T}$ into 200 evenly spaced bins between 0 and 1. The joint distribution is then computed with an element-wise multiplication over the bins to get a histogram approximation of $P(t_{WGD}|\boldsymbol{\theta}_{2+1}, \boldsymbol{\theta}_{2+0}, \boldsymbol{\theta}_{2+2})$. The midpoint of each bin is used as the bin's corresponding WGD timing value for downstream analysis.

8.6 Hit and run algorithm

To obtain uniform samples of valid solutions to equation 12, we use an MCMC hit and run algorithm conceptually similar to the approach implemented in the `walkr` package (19). The algorithm starts at an initial solution to equation 12, and each new position is found by moving in a direction defined by a vector uniformly sampled from the null space of $\mathbf{A}_C^R$, ensuring that each $\mathbf{t} \geq \mathbf{0}$. Periodically the chain is reset, and if the tumor is WGD, new WGD constraints for $\mathbf{A}_C$ are sampled from the WGD posterior timing distribution obtained in section 8.5.

Algorithm Hit and run gain timing sampling

```
procedure HITANDRUN(A_C^R, N, Target Size)
    Timing Store ← [ ]                         ▷ Initialize empty array to store states
    n ← rank A_C^R
    while Timing Store Size < Target Size do
        if tumor is WGD then
            Sample WGD timing T_WGD from posterior
            Update timing constraints vector N with new T_WGD
        end if
        Find solution t_0 where A_C^R t_0 = N using non-negative optimisation
        for i = 1, 2, ..., SamplesPerChain do
            x ~ N(0_n, I_n)
            d ← (x / ||x||_2) null A_C^R           ▷ Obtain vector d in null A_C^R
            l_neg ← − max_i d[d>0]_i / t_{i−1}[d>0]_i   ▷ Minimum scale factor for d
            l_pos ← max_i d[d<0]_i / t_{i−1}[d<0]_i     ▷ Maximum scale factor for d
            λ ~ U(l_neg, l_pos)                      ▷ Sample scale factor for d
            t_i ← t_{i−1} + λd
            if i > BurnInSteps and i mod SkipSteps = 0 then
                Add t_i to Timing Store
            end if
        end for
    end while
end procedure
```

We set $BurnInSteps$ to 25 and $SkipSteps$ to 5. $SamplesPerChain$ was set to $BurnInSteps + 500 \cdot SkipSteps$ such that the number of stored values of $\mathbf{t}$ per chain was 500. In practice, we found that the initial solution is often in a region of the space that is difficult for the sampler to move away from. We therefore allow a very large number of initial steps, until $||\mathbf{t}_i - \mathbf{t}_0||_2 \geq 0.001$. We permit up to 50,000 steps for the initial move away from the start position, before then

applying a further 25 burn-in steps. The total number of timing samples, Target Size, was adjusted dynamically depending on the complexity of the route and the procedure for doing so is given in the next section.

This procedure was simplified in the case where $\mathbf{A}_C^R$ had rank one, corresponding to routes with a single independent gain. For computational efficiency, the states were sampled with 500 uniformly spaced intervals along the direction of the null space, with the limits given as in the main algorithm. The set of sampled timing states was then shuffled.

8.7 Ensuring sufficient density of samples

Increasing the number of sampled timing states improves the precision of the timing measurements and route probabilities. To ensure the most efficient use of computational resources, we implemented a process that sampled timing states up to a sufficient density. To account for the density of the full sampling process, we computed the density in a space formed by concatenating the timing and subclone proportion vectors.

The density of sampled states was approximated using a nearest neighbors approach. As a proxy for density, we computed the proportion of a random subset of states that had at least one neighboring sample within a Euclidean distance of 0.05. We defined the space to have reached a sufficient sampling density when this density measure reached 90%.

This density computation is periodically computed during the sampling process. Once the threshold is met, the sampling is stopped. Due to the curse of dimensionality, this density threshold can take a very long time to be reached for high dimension space. Therefore, we use a secondary backup threshold that stops sampling once 500,000 samples have been reached.

In practice, this means that some copy number states finish with a low density of sampled timing vectors. For example, the average final density across all

7+0 routes is 0.316 but is as low as 0.0006 for some routes. Nevertheless, it is clear from the tests using simulated data that the current densities permit the gain timing in complex segments to be measured with sufficient precision. With further work, a more sophisticated sampling procedure could be implemented that further optimizes the trade-off between run time and precision.

Currently, GRITIC samples uniformly across all possible timing states to compute the marginal likelihood for each route (equation 27). The gain timings conditioned on each route are obtained from resampling these states according to their multiplicity likelihood. An accurate timing distribution could be obtained with substantially fewer samples if the posterior gain timing for each route was sampled directly, perhaps using a Metropolis-Hastings approach. However, computing the marginal likelihood of each route directly from the posterior gain timing distribution is significantly more involved (20, 21) and we find that the current approach works well in practice.

8.8 Subclonal multiplicity simplification

Each additional subclonal cluster increases the dimension of the sampled space and thus the number of samples required to adequately cover it. However, the proportion of SNVs in each subclone is not relevant for the gain timing, we are only interested in the proportion of clonal SNVs. Therefore, to aid convergence, we limit each tumor to two subclones.

For any tumor with more than two subclones, the subclone with the largest CCF is maintained and the smaller subclones are merged by taking the average CCF of the subclones weighted by the number of SNVs they contain. This improves inference speed on tumors with a large number of subclones and we do not expect that it will impact the accuracy of estimating gain timing from clonal SNVs.

8.9 Counting the number of events

We calculate the total number of events that correspond to each route such that a high number of events can be penalized to provide a lower bound on non-parsimony. Calculating the number of gain events is trivial and only dependent on the tree structure. It is simply the sum of all independent gain nodes in a given tumor plus an additional event corresponding to the WGD if the tumor is classified as such.

In the case of non-WGD tumors, the only loss that can be identified is a single loss if the minor copy number is zero. Identifying the number of losses for a route is more involved for WGD tumors. A minor copy number of zero or one implies a pre- or post-WGD loss respectively (Fig. M22). However as discussed in the main text, there are a number of implicit losses that can be identified in WGD tumors from the relative timing of gains and the WGD.

We assume a single loss event must have occurred between all connections of two non-WGD nodes with timing values that intersect the WGD timing (Fig. M22). This is because the WGD must have occurred at some point between the events corresponding to the two nodes, and yet the gains resulting from the WGD are not observed in this branch of the route tree. Therefore, a loss must have occurred to remove the gained allele resulting from the WGD on this branch.

Hence, given a gain timing $\mathbf{t}$, WGD timing t_{WGD} and tree corresponding to the route R, the number of implied losses is equal to the number of edges between non-WGD corresponding nodes that connect two nodes with timing that intersects t_{WGD}. To estimate the number of implied losses undergone by the segment given a particular route R, we take the average of the number of implied losses from 300 independent samples from the posterior distribution of $\mathbf{t}$ given R. We found that using 300 samples from the gain timing posterior was sufficient to give a consistent estimate of the number of loss events that depend on the gain timing, with an average standard error of 0.016.

The average number of events for each route is equal to the sum of the number of gain and WGD events, the number of implied losses resulting from the WGD and any fixed losses that can be identified from the minor copy number.

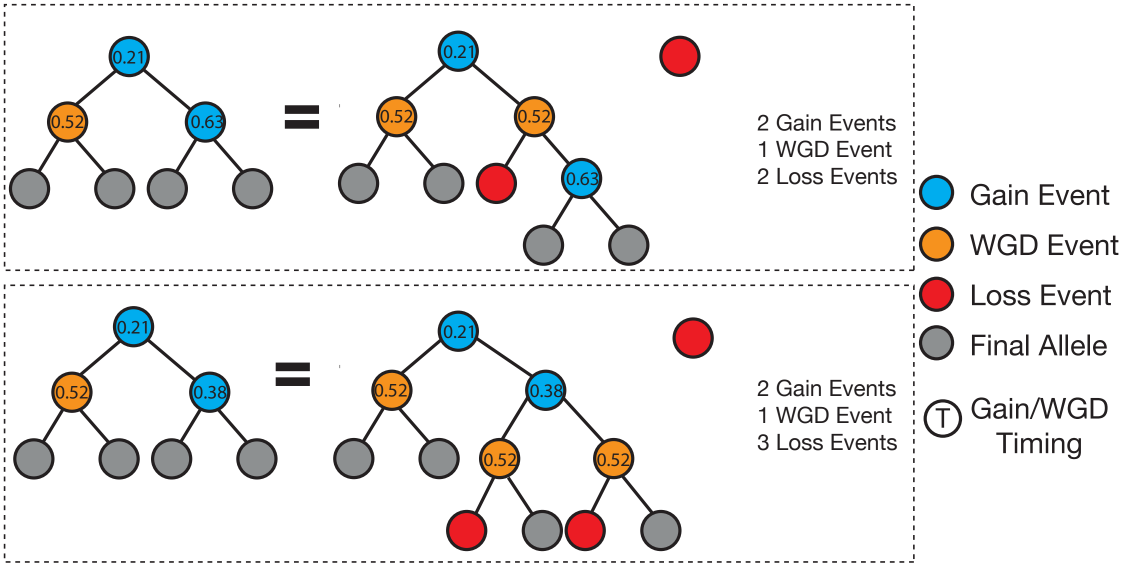

Figure M22: Example of the different number of loss events implied by different gain timings for a particular route leading to a 4+0 state given a WGD timing.

9 Other methods

9.1 Cancer type annotation

As stated in the main text, the cancer type classifications for each sample were obtained from a unified annotation of the PCAWG and Hartwig cohorts (22). The cancer type information for 143 PCAWG and 775 Hartwig tumors was not available from this set of unified annotations.

We obtained the cancer type information for these tumors through a mapping approach. For all tumors with a unified annotation, we mapped the cancer type metadata information from the original metadata provided from PCAWG and

Hartwig to the unified annotation. A mapping was considered reliable if more than 80% of the tumors with a given set of cohort cancer type metadata mapped to a single unified annotation. We then used the reliable metadata mappings to obtain a unified cancer type information for all 143 of the remaining PCAWG tumors and 694 of the Hartwig tumors. This left 81 Hartwig tumors without a unified cancer type annotation, which were removed from the analysis.

9.2 Sample filtering

Across PCAWG and Hartwig, whole-genome sequencing processed through the Hartwig pipeline was obtained for 7,890 tumors. We then applied several quality filters to this cohort.

To ensure sufficient coverage for SNV multiplicity inference, we required samples with an NRPCC of at least 5, a threshold set from simulation (Fig. S50). NRPCC is the expected number of reads in a tumor for a clonal SNV with a multiplicity of one (7). It is calculated as:

$$NRPCC = \frac{\rho}{\rho\Psi_T + \Psi_N(1-\rho)}d \tag{30}$$

Here, ρ is the tumor purity, Ψ_T is the tumor ploidy, d is the depth of sequencing and Ψ_N is the normal ploidy (2 for females, 1.93 for males). In total, 61 tumors had an NRPCC of less than 5. For patients with multiple tumor samples, we selected the sample labeled as preferred from the PCAWG cohort and one representative sample that had the highest NRPCC in the Hartwig cohort. This led to the removal of 398 tumor samples. As described in 9.1, we filtered the 81 samples from the Hartwig cohort where we were unable to obtain a unified cancer type annotation.

We filtered samples in which the major copy number mode was greater than two. This is because these tumors likely had more than one WGD, an evolutionary history that is currently not supported by GRITIC. This encompassed

456 tumors in both cohorts. The 175 tumors with a major copy number mode of two but with less than 60% overlap of the timing of major copy number two segments by total base pairs were also excluded, as they were likely genome duplicated, but violated the principle of simultaneous gains required by GRITIC. We also removed 81 samples from the Hartwig cohort with insufficient cancer type annotation.

Only clonal copy number gains in segments with at least 20 SNVs were timed, a threshold also found to be sufficient for accurate inference from simulations (Fig. S50). To ensure that the inference for each sample was completed in a reasonable timeframe, we restricted GRITIC to timing copy number states with no more than 500 possible routes for WGD tumors (Supplementary Table 1).

Of the tumors with a major copy number mode of one or two, 757 tumors from the combined cohort were not included as they contained no independently gained regions with sufficient SNVs.

After applying all filters together, 1,799 tumors were removed. This left 6,091 tumors for the analysis.

9.3 Sample simulation

9.3.1 Representative cohorts

We tested GRITIC using simulated samples with known ground-truth gain timing and route histories, aiming to make the simulations as representative of true samples as possible. We randomly selected a tumor with a major copy number mode of two from Hartwig and PCAWG to form the basis of each simulated sample. The original copy number profile and purity of the selected base tumors were maintained in the simulated sample.

The true WGD timing was sampled from a uniform distribution from 0.01 to 0.99. The multiplicity proportions were set to match the simulated ground-truth timing for all segments with at least 20 SNVs, the same threshold used in the anal-

ysis of real samples. Segments with major copy number two were simulated to have multiplicity proportions corresponding to a gain occurring at the time of the WGD. The gain timing and corresponding clonal multiplicity proportions for higher copy number states were simulated by selecting the 250th sample from our MCMC sampler with a given route. The proportion of subclonal mutations was directly obtained from SNV clustering information for each sample. As in the main GRITIC method, all subclonal SNVs were assumed to have a multiplicity of one.

The number of SNVs in each multiplicity state was obtained from a multinomial draw using clonal and subclonal multiplicity proportions and the number of SNVs in the gained segment from the base tumor. Alternative read counts for each SNV were then drawn from a binomial distribution using the variant allele frequency equation (7) for the given multiplicity and total coverage drawn from a Poisson distribution with a mean equal to the average read coverage of the segment in the base sample.

Two cohorts were simulated, one where all routes for a given copy number state were sampled with equal probability, one where only the most parsimonious routes were selected. 5000 samples were simulated for each of these cohorts.

9.3.2 Cohort with uniform gain timing

To obtain a control cohort for our gain timing relative to WGD analysis, we simulated an additional cohort where routes were sampled such that gain timing was approximately uniformly distributed for any WGD timing. To account for any possible relationship between copy number state and WGD timing, the true WGD timing for each sample in this cohort was taken from the corresponding true WGD timing of the base sample profile.

A heuristic approach was used to obtain a cohort with approximately uniformly distributed gain timing. For each sample, we selected the true route per segment such that each sample had a gain timing distribution that was approxi-

mately uniform. To obtain a simulated sample, the true gain timing was first obtained as above for all possible routes in each segment. Then we sampled 1,000 combinations of one route per segment and tested each combination for uniform gain timing. We evaluated the proportion of true gains that occurred pre-WGD, weighted by base pair length. A combination was rejected if this proportion differed from the true WGD gain timing by more than 0.1, ensuring that copy number gains accumulated roughly linearly with respect to mutation time. We then selected the final combination of true routes for a sample was selected based on the combination that gave a gain timing with the highest p-value using Kolmogorov–Smirnov test against a uniform distribution from 0 to 1. 2500 samples were simulated for this cohort.

9.3.3 Simulation for setting minimum quality thresholds

The samples were simulated using a similar method to the representative cohort. As before, a tumor with major copy number mode of two was randomly chosen from the PCAWG and Hartwig cohort to serve as a base copy number and subclonal profile. However, the depth of sequencing and tumor purity were randomly generated for each of these samples by randomly sampling an NRPCC and tumor purity. The sequencing depth for each segment with total copy number n_T was sampled from a poisson distribution with average depth d given by:

$$d = \frac{NRPCC(n_T\rho + 2(1-\rho))}{\rho} \tag{31}$$

As before, a true WGD timing was randomly sampled for each simulated tumor. The timing of the major copy number two segments in the base profile were simulated according to the true WGD timing and the true number of mutations.

All segments with major copy number greater than two were removed from the base profile and replaced with simulated segments with varying numbers of

mutations from a randomly selected copy number state. The true timing, and multiplicity proportions for each gained segment were generated identically to the approach for the representative simulated cohorts. 5001 samples were simulated for this cohort.

9.4 Assessing route probability calibration in simulated data

We assessed the calibration of the gain route probabilities inferred from GRITIC using a binning approach. We placed the probability of each gain route for all simulated segments in 20 evenly spaced bins from zero to one. The true probability for each bin was then calculated from the mean number of routes in each bin that were the true simulated route for the corresponding segment. The model was judged to be well calibrated if this true probability fell within the corresponding bin.

9.5 Measuring agreement between route histories

As a further validation of GRITIC, we conducted two analyses to test the agreement of the route histories for inferred by GRITIC for major copy number 3 and 4 segments, the two most common states. We tested the agreement of route histories using two approaches, one to test agreement within individual chromosomes and a second to test agreement across the cohort.

9.5.1 Measuring route history agreement within a chromosome

We classified segments on each chromosome as having either a pre-WGD or post-WGD gain and tested whether the agreement between segments on the same chromosome was greater than expected from the sample as a whole. We assigned each segment as undergoing a pre-WGD gain if at least 50% of the posterior samples from the joint route timing distribution had at least one pre-WGD gain, otherwise it was given a post-WGD assignment. The route

assignments of all possible pairs of major copy number three and major copy number four segments in the same chromosome were counted. This was compared to a permutation model where the route assignments for each segment were permuted between segments of the same major copy number state within each sample.

9.5.2 Measuring route history agreement across samples

In our second validation, we checked whether different chromosomes had similar tendencies to be gained pre-WGD for both major copy number 3 and 4 states. We computed the probability that a chromosome was gained pre-WGD by averaging the pre-WGD gain posterior probability for all segments on the chromosome, weighted by base pair length. Only one of major copy number three or major copy number four segments were considered for a given chromosome, whichever copy number state had the highest total base pair length. Each chromosome required a minimum 1Mb total gain of either major copy number three or four to be included. The pre-WGD gain probabilities for different chromosomes were averaged across samples in the PCAWG and Hartwig cohort for each major copy number state.

9.6 Inferring punctuated gains

We aimed to identify tumors that had gains in different chromosomes that occurred closer together in mutation time than would be expected under independent draws from the distribution of gain timing. Such samples were classified as having a synchronous or punctuated set of gains. Following an approach similar to that of Gerstung *et al.* (6), we performed a permutation-based procedure at the tumor level to obtain a threshold to define synchronous gains for each sample. We restricted this analysis to tumors that had at least 15 samples of the same primary tumor type. Additionally, tumors with fewer than five gained chromosomes were excluded and classified as uninformative.

To identify synchronous gains in each sample, we defined a synchronicity score for each sample, which we term the median weighted gain timing distance. First, we created pseudo-samples with exact gain timing for each segment by drawing from the posterior timing distribution of each gained segment. We calculate the point in the mutation time that has the minimum distance to the timing of all segments weighted by the segment width. For segments with multiple gains, only the distance between the gain and closest timing was measured. A total of 100 pseudo-samples were drawn for each sample, and the median minimum weighted gain timing distance across the samples was computed.

We then compared the median distance to that obtained under a null distribution, obtained with 1,000 independent permutations performed with different random seeds for all samples using the curveball method (23), which randomly swaps chromosomes across patients within the same cancer type, while maintaining the number of chromosomes gained per patient and the number of gains per chromosome across the cohort.

Each tumor was independently defined as punctuated if its median weighted gain timing distance was lower than 95% of the permuted samples for that tumor. This can be interpreted as gains within a sample occurring closer in mutational time than expected by chance, for a given cancer type.

9.7 Assessing non-parsimony from cell line data

We used existing cell line data with provided copy number calls (24). We examined the copy number of tetraploid clones at passages four and 50 and measured any change in copy number at each base pair between passages. We plotted the transitions weighted by segment width between major copy number two, three and four segments at passage four, and major copy number three and four at passage 50. Each transition was labeled as parsimonious if the major copy number at passage four would have been expected under the most parsimonious route history expected from the major copy number at passage

50.

9.8 Measuring non-parsimony in clear cell renal cell carcinomas

We selected the 54 genome-duplicated clear cell renal cell carcinomas from PCAWG and Hartwig. We then selected segments from these samples with a major copy number of three or four, a minor copy number of no more than two and at least a 50% probability on the most parsimonious or non-parsimonious route(s) with the fewest number of events.

These copy number states and routes were selected because they have a clear relationship between parsimony and gain timing. For segments with a major copy number of four, the most parsimonious route had the first gain pre-WGD, and the two non-parsimonious routes with the joint lowest number of events had the first gain post-WGD. The reverse is true for major copy number three segments, where the sole non-parsimonious route with the fewest number of events has a single pre-WGD gain.

We aggregated the average non-parsimonious probability for each chromosome weighted by segment base pair length, normalizing the route probabilities within the selected routes. Statistical significance was calculated using a permutation test and 95% confidence intervals by bootstrapping samples.

This analysis was restricted to the two most parsimonious routes for copy number states with major copy numbers of three and four and a minor copy number of no more than two 2 as these routes have a clear relationship between route gain timing and parsimony/non-parsimony. We only included segments that had at least 50% of their posterior probability on the two most parsimonious routes. With the non-parsimony penalty, this accounted for 95.9% by base pair length of the gains in the clear cell renal cell carcinomas. Without the penalty, the fraction was reduced to 72.8%, but similar patterns in terms of non-parsimony

were observed.

9.9 Measuring CIN in single cells

We utilized 10x single-cell whole genome sequencing data from a single undifferentiated soft tissue sarcoma (25). The tumor contained three distinct tumor cell sub-populations with average ploidies of 1.2, 2.4, and 3.9, respectively, which were determined by fluorescence-activated cell sorting.

Based on these ploidies and their copy number profiles, the diploid and tetraploid populations are likely to represent cells resulting from two rounds of WGD following an initial haploidization event. We ran ASCAT.sc (`https://github.com/VanLoo-lab/ASCAT.sc`) using phased germline SNPs generated by Battenberg (15) on a matched normal to obtain allele-specific copy number profiles for all tumor cells. Noisy cells were removed based on the standard filtering metrics in ASCAT.sc.

Cells with a higher ploidy can have genomes with a greater number of potential copy number states. To obtain an estimate of CIN corrected for this bias, all cell populations were fitted with copy number profiles with ploidy between 3.7 and 5.5.

We then used MEDICC2 (26) to build copy number phylogenies for each subpopulation separately, with a minimum segment length of 2.5Mb. The copy number heterogeneity of each population was inferred by measuring the number of copy number events required to transform the common ancestral profile, as inferred by MEDICC2, into each of the cells in the subpopulation.

To account for a small number of cells with noisy copy number profiles which were not removed by ASCAT.sc, the ancestral profile was defined as the profile inferred by MEDICC2 to be the ancestor of 90% (or the closest possible fraction) of cells in the population. Only cells that were measured to be descendants of this ancestral profile were considered.

9.10 Classifying segments in punctuated gains

We classified the gained segments based on their base pair length, overlapping structural variants and chromosomal position. A gain was classified as intra-chromosomal if there existed a corresponding SV junction within the same chromosome and inter-chromosomal for a corresponding SV junction to a different chromosome.

The form of the gained segments were classified into 6 categories based on their chromosomal position. Only PCAWG segments were classified as high-confidence consensus SV calls were available for this cohort.

	Whole Chromosome (>99% of chromosome)	Spans Centromere (breakpoint on either end of centromere)	Telomere-Bounded (breakpoint within 1% of telomere)	Centromere-Bounded (breakpoint within 1% of centromere)
Whole Chromosome	Yes			
Supra-Centromeric Arm	No	Yes	Yes	
Whole Arm			Yes	Yes
Sub-Arm Segment (Telomere-Bounded)		No	Yes	Yes
Sub-Arm Segment (Centromere-Bounded)			No	No
Sub-Arm Segment (Interstitial)			No	

Table M1: Classification criteria for gained segments based on their chromosomal position.

9.11 Nearest neighbor tests

For each route that can lead to the 20 most common complex copy number states, we ran a chain that sampled 20,000 valid timing vectors using the hit and run approach outlined above. To assess convergence, we partitioned this data into two equal sections, the first and last 9,000 samples. Therefore, 18,000 samples were included for each copy number state. This was computed separately for 11 different WGD timing values between 0.01 and 0.99. As the WGD timing was fixed rather than sampled from a distribution, the chain was run

continually for 20,000 steps instead of being periodically reset, as in GRITIC.

Then as before, each sampled timing state was converted into multiplicity states using equation 11. For each copy number state and WGD timing, we took 1,000 multiplicity states at random and collected their 10 nearest neighbors from the remaining multiplicity states. We assessed the proportion of these nearest neighbors that were from the same copy number route.

We validated that the convergence of the chain did not confound the estimate of nearest neighbors. For each multiplicity state under consideration, we computed the proportion of nearest neighbors with the same route that originated from the same half of the chain as the multiplicity state. Aggregated across all timing states, this proportion was very close to 0.5 for all copy number states (Fig. M23). This suggests that the copy number states had been adequately sampled and the nearest neighbors with the correct route weren't just multiplicity proportions that were sampled in close succession.

It is worth noting that routes with a single degree of freedom have a different sampling procedure that will bias this estimate. As discussed in section 8.6, the single degree of freedom is sampled with uniform spacing between the valid limits before being shuffled. The shuffling of the obtained states would mean a proportion of matching nearest neighbors of 0.5 would be expected. However, 1,010 out of the possible 1,019 routes across all of the evaluated copy number states have more than one degree of freedom.

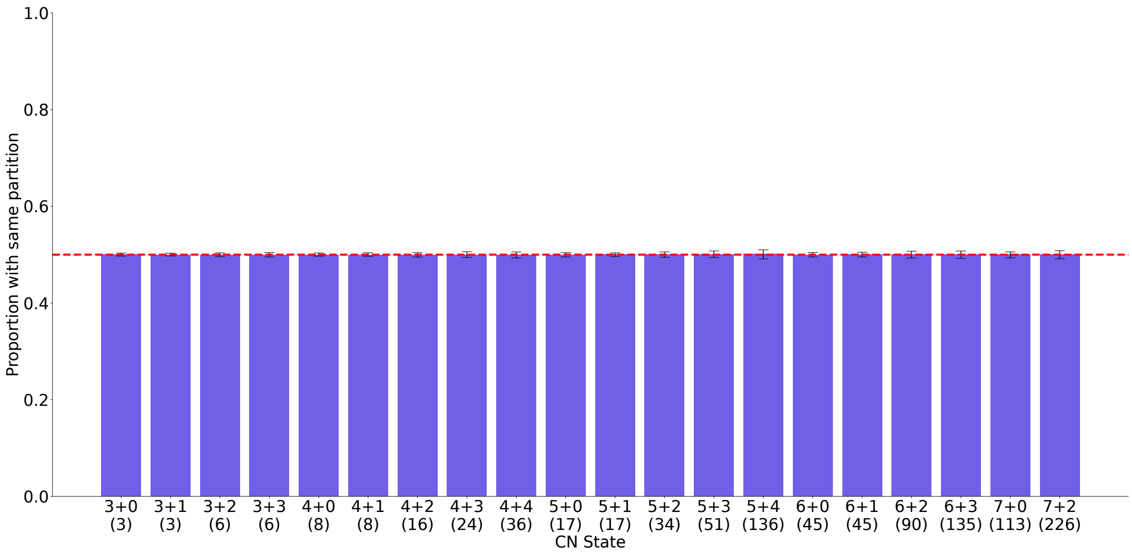

Figure M23: The proportion of nearest neighbors with the same route that arise from the same half of the multiplicity sampled chain as the multiplicity state under consideration. The dashed line indicates the 50% proportion that would be expected under convergence. The number of possible routes for each copy number state is given in brackets.

References

1. Kimura, M. The number of heterozygous nucleotide sites maintained in a finite population due to steady flux of mutations. *Genetics* **61,** 893–903 (1969).
2. Durinck, S *et al.* Temporal dissection of tumorigenesis in primary cancers. *Cancer Discovery* **1,** 137–143 (2011).
3. Purdom, E, Ho, C, Grasso, CS, Quist, MJ, Cho, RJ & Spellman, P. Methods and challenges in timing chromosomal abnormalities within cancer samples. *Bioinformatics* **29,** 3113–3120 (2013).
4. Greenman, CD *et al.* Estimation of rearrangement phylogeny for cancer genomes. *Genome Research* **22,** 346–361 (2012).
5. Jolly, C & Van Loo, P. Timing somatic events in the evolution of cancer. *Genome Biology* **19,** 95 (2018).

6. Gerstung, M *et al.* The evolutionary history of 2,658 cancers. *Nature* **578,** 122–128 (2020).
7. Tarabichi, M *et al.* A practical guide to cancer subclonal reconstruction from DNA sequencing. *Nature Methods* **18,** 144–155 (2021).
8. Nik-Zainal, S *et al.* Mutational processes molding the genomes of 21 breast cancers. *Cell* **149,** 979–993 (2012).
9. Alexandrov, LB *et al.* The repertoire of mutational signatures in human cancer. *Nature* **578,** 94–101 (2020).
10. Köster, J, Dijkstra, LJ, Marschall, T & Schönhuth, A. Varlociraptor: enhancing sensitivity and controlling false discovery rate in somatic indel discovery. *Genome Biology* **21,** 98 (2020).
11. Demeulemeester, J, Dentro, SC, Gerstung, M & Van Loo, P. Biallelic mutations in cancer genomes reveal local mutational determinants. *Nature Genetics* **54,** 128–133 (2022).
12. Nik-Zainal, S *et al.* The life history of 21 breast cancers. *Cell* **149,** 994–1007 (2012).
13. Leshchiner, I *et al.* Inferring early genetic progression in cancers with unobtainable premalignant disease. *Nature Cancer* **4,** 550–563 (2023).
14. Shervashidze, N, Schweitzer, P, Leeuwen, EJv, Mehlhorn, K & Borgwardt, KM. Weisfeiler-Lehman Graph Kernels. *Journal of Machine Learning Research* **12,** 2539–2561 (2011).
15. Hagberg, AA, Schult, DA & Swart, PJ. *Exploring Network Structure, Dynamics, and Function using NetworkX* in *Proceedings of the 7th Python in Science Conference* (Pasadena, CA USA, 2008), 11–15.
16. Satas, G, Zaccaria, S, El-Kebir, M & Raphael, BJ. DeCiFering the elusive cancer cell fraction in tumor heterogeneity and evolution. *Cell Systems* **12,** 1004–1018 (2021).
17. Dentro, SC, Wedge, DC & Van Loo, P. Principles of Reconstructing the Subclonal Architecture of Cancers. *Cold Spring Harbor Perspectives in Medicine* **7** (2017).

18. Roth, A *et al.* PyClone: statistical inference of clonal population structure in cancer. *Nature Methods* **11,** 396–398 (2014).
19. Yao, A & Kane, D. walkr: MCMC Sampling from Non-Negative Convex Polytopes. *The Journal of Open Source Software* **2,** 61 (2017).
20. Wang, YB, Chen, MH, Kuo, L & Lewis, PO. A New Monte Carlo Method for Estimating Marginal Likelihoods. *Bayesian Analysis* **13** (2018).
21. Llorente, F, Martino, L, Delgado, D & Lopez-Santiago, J. *On the computation of marginal likelihood via MCMC for model selection and hypothesis testing* in *2020 28th European Signal Processing Conference* (IEEE, Amsterdam, Netherlands, 2021), 2373–2377.
22. Martínez-Jiménez, F *et al.* Pan-cancer whole-genome comparison of primary and metastatic solid tumours. *Nature* **618,** 333–341 (2023).
23. Strona, G, Nappo, D, Boccacci, F, Fattorini, S & San-Miguel-Ayanz, J. A fast and unbiased procedure to randomize ecological binary matrices with fixed row and column totals. *Nature Communications* **5,** 4114 (2014).
24. López, S *et al.* Interplay between whole-genome doubling and the accumulation of deleterious alterations in cancer evolution. *Nature Genetics* **52,** 283–293 (2020).
25. Steele, CD *et al.* Signatures of copy number alterations in human cancer. *Nature* **606,** 984–991 (2022).
26. Kaufmann, TL *et al.* MEDICC2: whole-genome doubling aware copy-number phylogenies for cancer evolution. *Genome Biology* **23,** 241 (2022).
