# Supplementary material for: The history of chromosomal instability in genome doubled tumors
Source: Cancer Discov. Author manuscript; Available in PMC 2024 Sep 19. (PMC7616501; doi:10.1158/2159-8290.CD-23-1249)
Supplement: Supplementary Figures [file EMS197414-supplement-Supplementary_Figures.pdf]

The history of chromosomal instability in genome doubled tumors
Supplementary Figures

List of Figures

S1 WGD frequencies across cancer types and stage . . . 3
S2 Effect of WGD constraint on timing accuracy . . . 4
S3 Measuring timing accuracy on simulated data . . . 5
S4 Measuring timing accuracy on simulated data by copy number state . . . 6
S5 Measuring timing accuracy on simulated data by copy number state . . . 7
S6 Measuring timing accuracy on simulated data by copy number state . . . 8
S7 Measuring timing accuracy on simulated data by copy number state . . . 9
S8 Measuring inferred route probabilities on simulated data . . . 10
S9 Measuring inferred route probabilities on simulated data . . . 11
S10 Measuring inferred route probabilities on simulated data . . . 12
S11 Measuring inferred route probabilities on simulated data . . . 13
S12 Timing of gains in multi-region tumors . . . 14
S13 Difference in timing between different gain routes . . . 15
S14 Difference in timing between different gain routes . . . 16
S15 Non-parsimony in copy number gain evolution . . . 17
S16 Non-parsimony by copy number state . . . 18
S17 Calibrating a penalty on non-parsimony . . . 19
S18 Calibrating a penalty on non-parsimony . . . 20
S19 Clear-cell sample gain timing . . . 21
S20 Gain route agreement within chromosomes . . . 22
S21 Probability of pre-WGD gains in different chromosomes and copy number states . . . 23
S22 Distribution of gain timing by major copy number . . . 24
S23 Single cell copy number profiles of an undifferentiated sarcoma . . . 25
S24 Distribution of gain rates relative to WGD by cancer type . . . 26
S25 Distribution of gain rates relative to WGD compared to simulations . . . 27
S26 Example sample gain timing posterior . . . 28
S27 Combined distribution over gain timing by WGD status . . . 29
S28 The timing of gains relative to WGD . . . 30
S29 The timing of gains relative to WGD by cancer type . . . 31
S30 Proportion of copy number events post-WGD . . . 32
S31 The relationship between genome gained post-WGD and WGD timing by cancer type . . . 33
S32 The relationship between genome gained pre-WGD and WGD timing by cancer type . . . 34
S33 The relationship between fraction of genome lost pre and post-WGD and WGD timing by cancer type 35
S34 Punctuated gains in WGD tumors . . . 36
S35 Association between chromothripsis and punctuated gains . . . 37
S36 Genomic features of punctuated gains . . . 37
S37 Frequency of arm gains pre and post-WGD and in non-WGD tumors . . . 38
S38 Frequency of arm gains pre and post-WGD and in non-WGD tumors by cancer type . . . 39
S39 Frequency of arm losses pre and post-WGD and in non-WGD tumors by cancer type . . . 40
S40 Effect of oncogene and tumor suppressor gene density on arm gain rates . . . 41
S41 Effect of oncogene and tumor suppressor gene density on arm loss rates . . . 42

S42 Pan-genome frequencies of pre and post-WGD gains by cancer type 43
S43 Pan-genome frequencies of pre and post-WGD gains by cancer type 44
S44 Pan-genome frequencies of pre and post-WGD gains by cancer type 45
S45 Pan-genome frequencies of pre and post-WGD gains by cancer type 46
S46 Pan-genome frequencies of pre and post-WGD gains by cancer type 47
S47 Pan-genome frequencies of pre and post-WGD losses by cancer type 48
S48 Pan-genome frequencies of pre and post-WGD losses by cancer type 49
S49 Pan-genome frequencies of pre and post-WGD losses by cancer type 50
S50 The effect of NRPCC and mutation count on gain timing inference 51
S51 WGD status calling in GRITIC 52
S52 The effect of the non-parsimony penalty on event timing 53

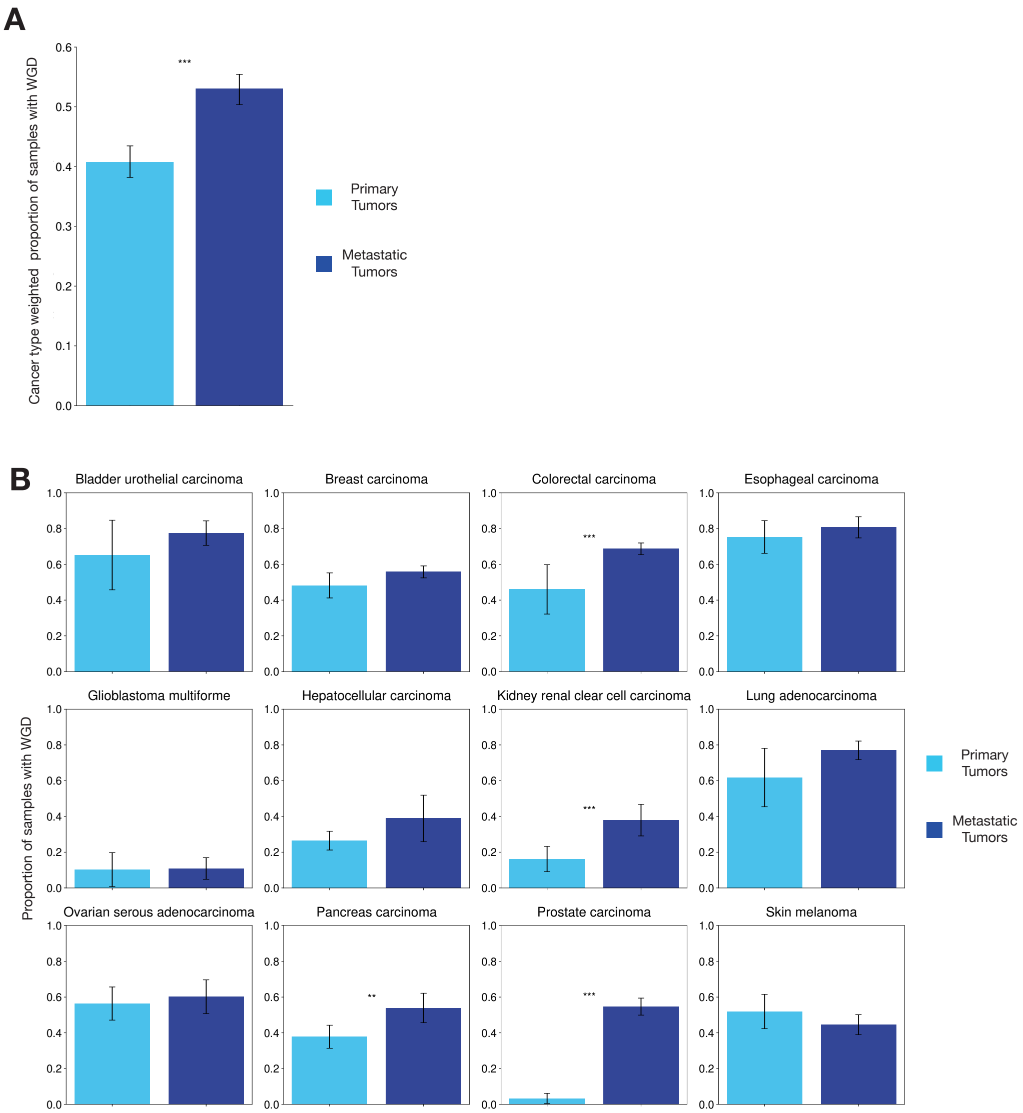

Figure S1: WGD frequencies across cancer types and stage. **A**, The proportion of tumor samples identified as WGD in primary and metastatic cohorts, weighted by cancer type. Only primary tumors with at least 20 primary and metastatic tumors are included. Statistical significance was calculated by proportion test and 95% confidence intervals by bootstrapping over samples. **B**, The proportion of tumor samples identified as WGD in primary and metastatic cohorts across different primary cancer types. Statistical significance was calculated by proportion test and 95% confidence intervals by normal approximation. ** and *** indicate comparisons where $p < 0.01$ and < 0.001 respectively.

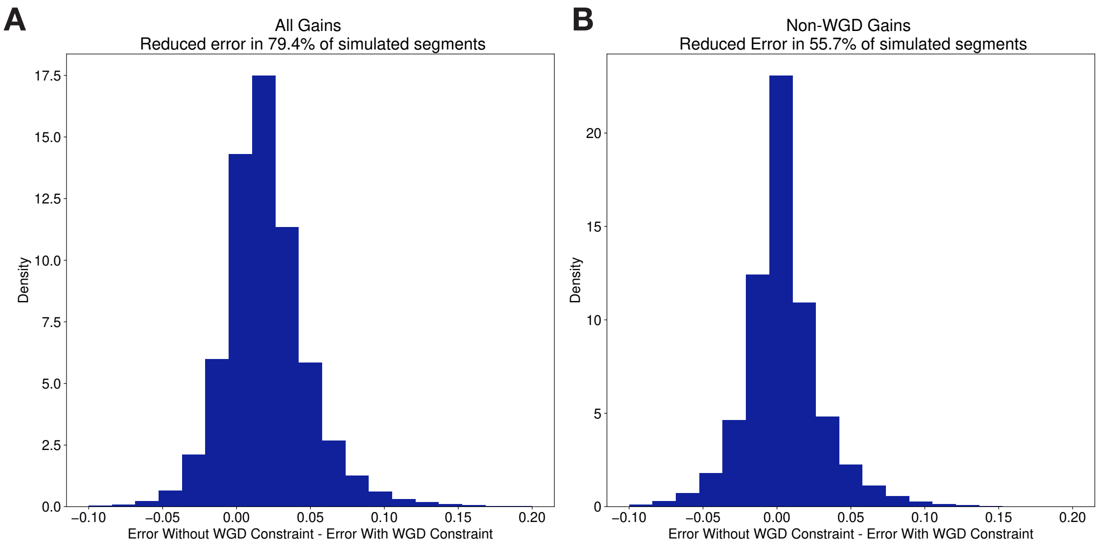

Figure S2: Effect of WGD constraint on timing accuracy. The difference in average error in segment gain timing across a set of representative simulated WGD tumors with and without a simultaneous WGD timing constraint applied to the sample during GRITIC inference. Displayed for all gains (**A**) and gains that did not arise during the WGD (**B**).

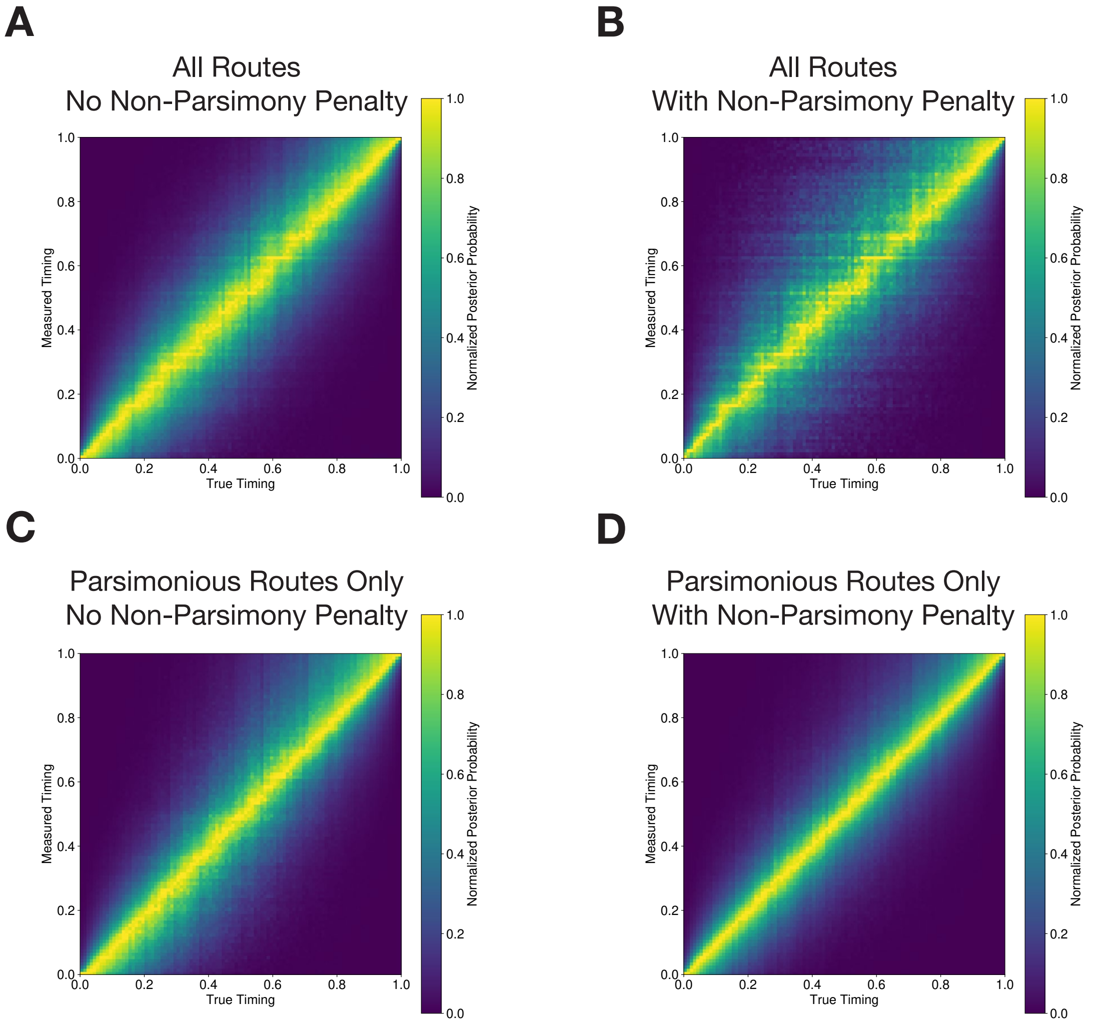

Figure S3: Measuring timing accuracy on simulated data. The distribution of measured posterior probability on gain timing against true simulated gain timing across representative cohorts simulated under different conditions. Each histogram column is divided by its maximum value such that the highest posterior probability per true gain timing bin is normalized to 1. **A**, A cohort simulated with all routes and without a penalty on non-parsimony applied during inference. **B**, A cohort simulated with all routes and with a penalty on non-parsimony applied during inference. **C**, A cohort simulated with parsimonious routes only and without a penalty on non-parsimony applied during inference. **D**, A cohort simulated with parsimonious routes only and with a penalty on non-parsimony applied during inference.

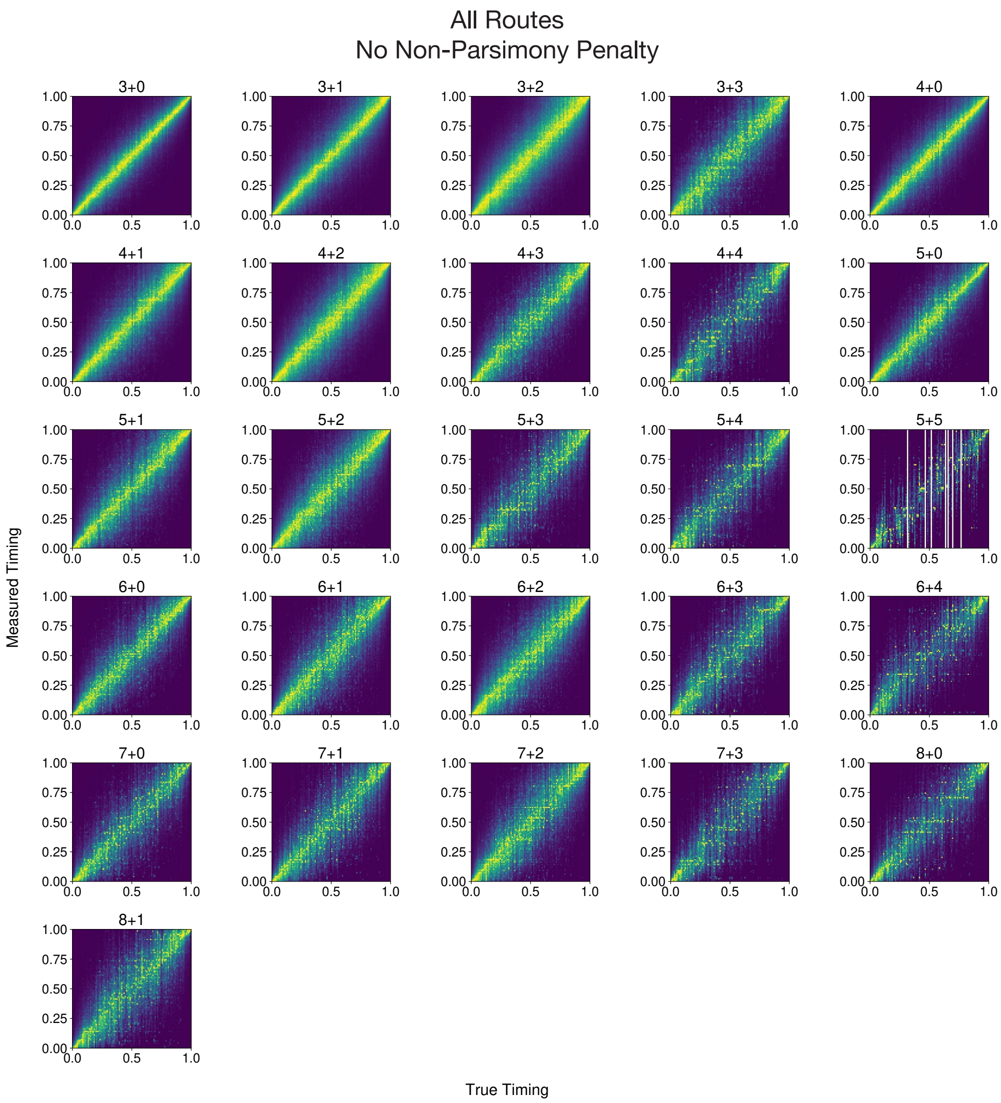

Figure S4: Measuring timing accuracy on simulated data by copy number state. Distribution of measured posterior probability on gain timing against true gain timing for a representative cohort of tumors where all gains from all routes are simulated as the ground truth, and no penalty is applied to non-parsimonious routes during inference. Each histogram column is divided by its maximum value such that the highest posterior probability per true gain timing bin is normalized to 1. True timing values where there was insufficient data for inference are indicated by white columns.

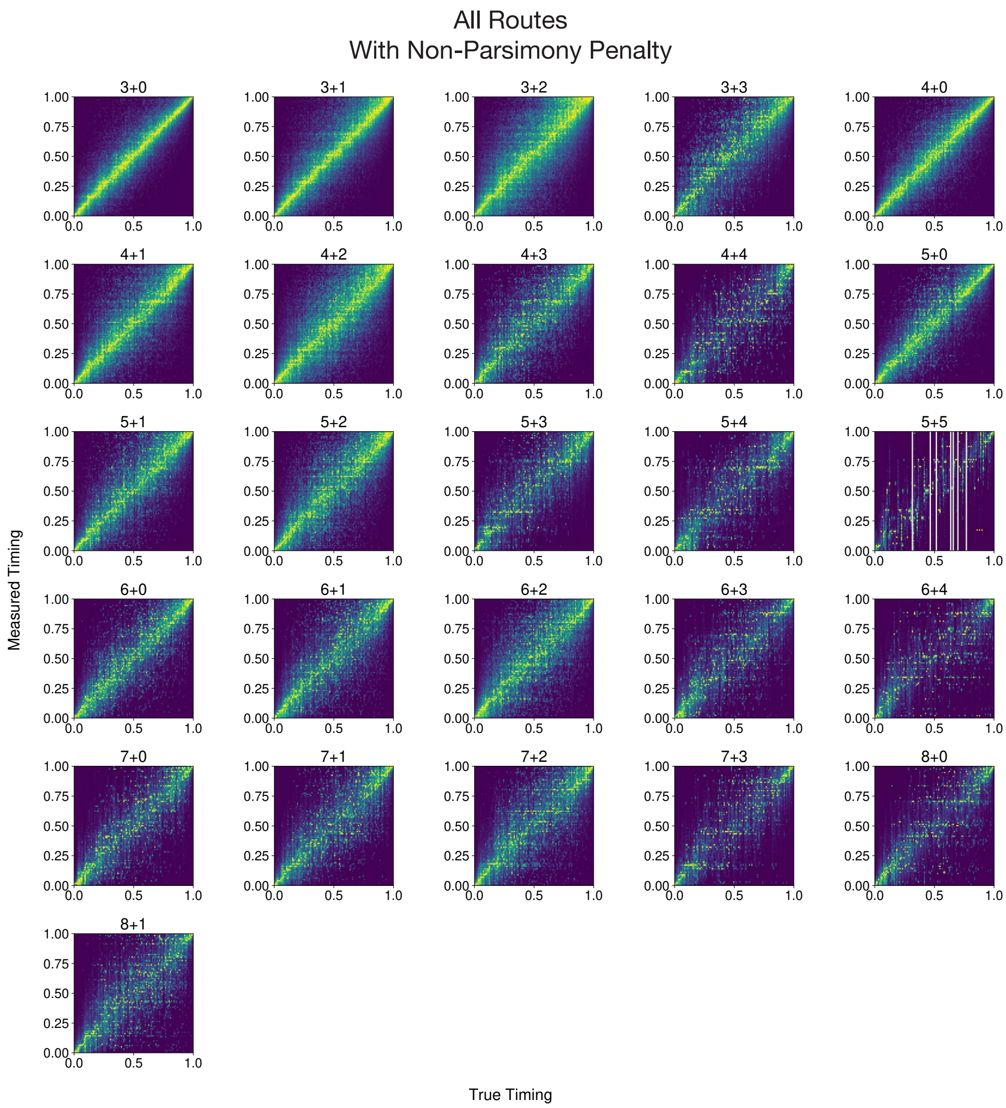

Figure S5: Measuring timing accuracy on simulated data by copy number state. Distribution of measured posterior probability on gain timing against true gain timing for a representative cohort of tumors where all gains from all routes are simulated as the ground truth, and a penalty is applied to non-parsimonious routes during inference (see Methods). Each histogram column is divided by its maximum value such that the highest posterior probability per true gain timing bin is normalized to 1. True timing values where there was insufficient data for inference are indicated by white columns.

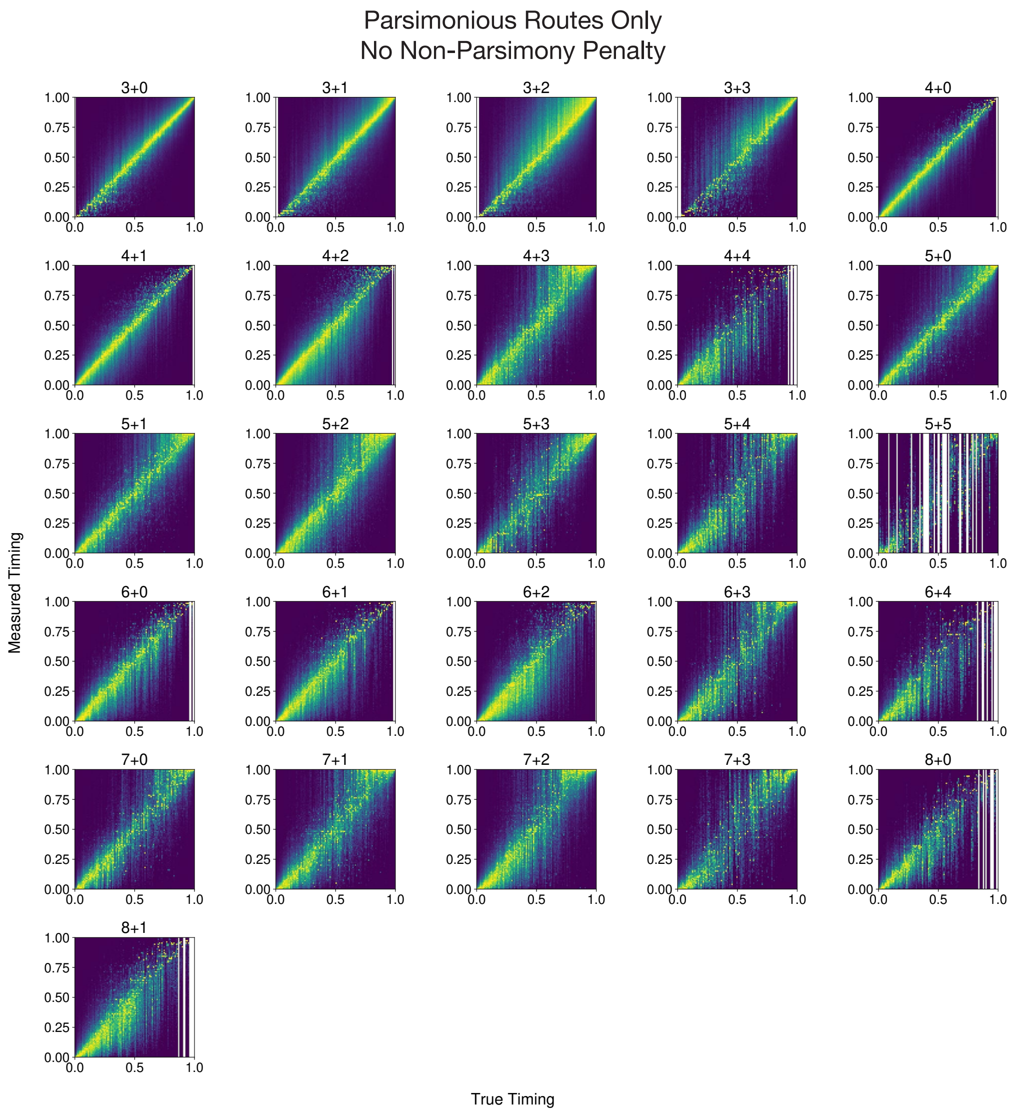

Figure S6: Measuring timing accuracy on simulated data by copy number state. Distribution of measured posterior probability on gain timing against true gain timing for a representative cohort of tumors where all gains from solely parsimonious routes are simulated as the ground truth, with no penalty applied to non-parsimonious routes during inference. Each histogram column is divided by its maximum value such that the highest posterior probability per true gain timing bin is normalized to 1. True timing values where there was insufficient data for inference are indicated by white columns.

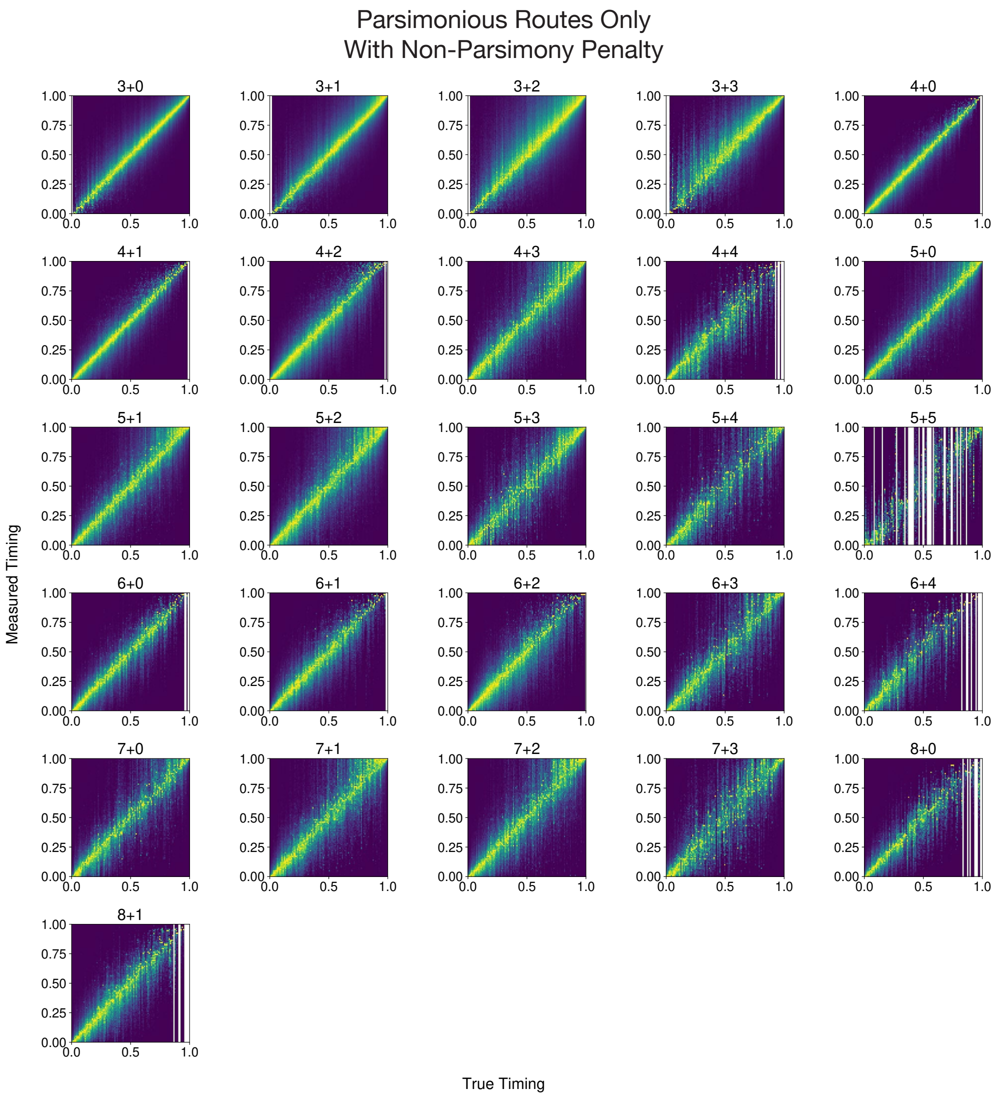

Figure S7: Measuring timing accuracy on simulated data by copy number state. Distribution of measured posterior probability on gain timing against true gain timing for a representative cohort of tumors where all gains from solely parsimonious routes are simulated as the ground truth, with a penalty applied to non-parsimonious routes during inference (see Methods). Each histogram column is divided by its maximum value such that the highest posterior probability per true gain timing bin is normalized to 1. True timing values where there was insufficient data for inference are indicated by white columns.

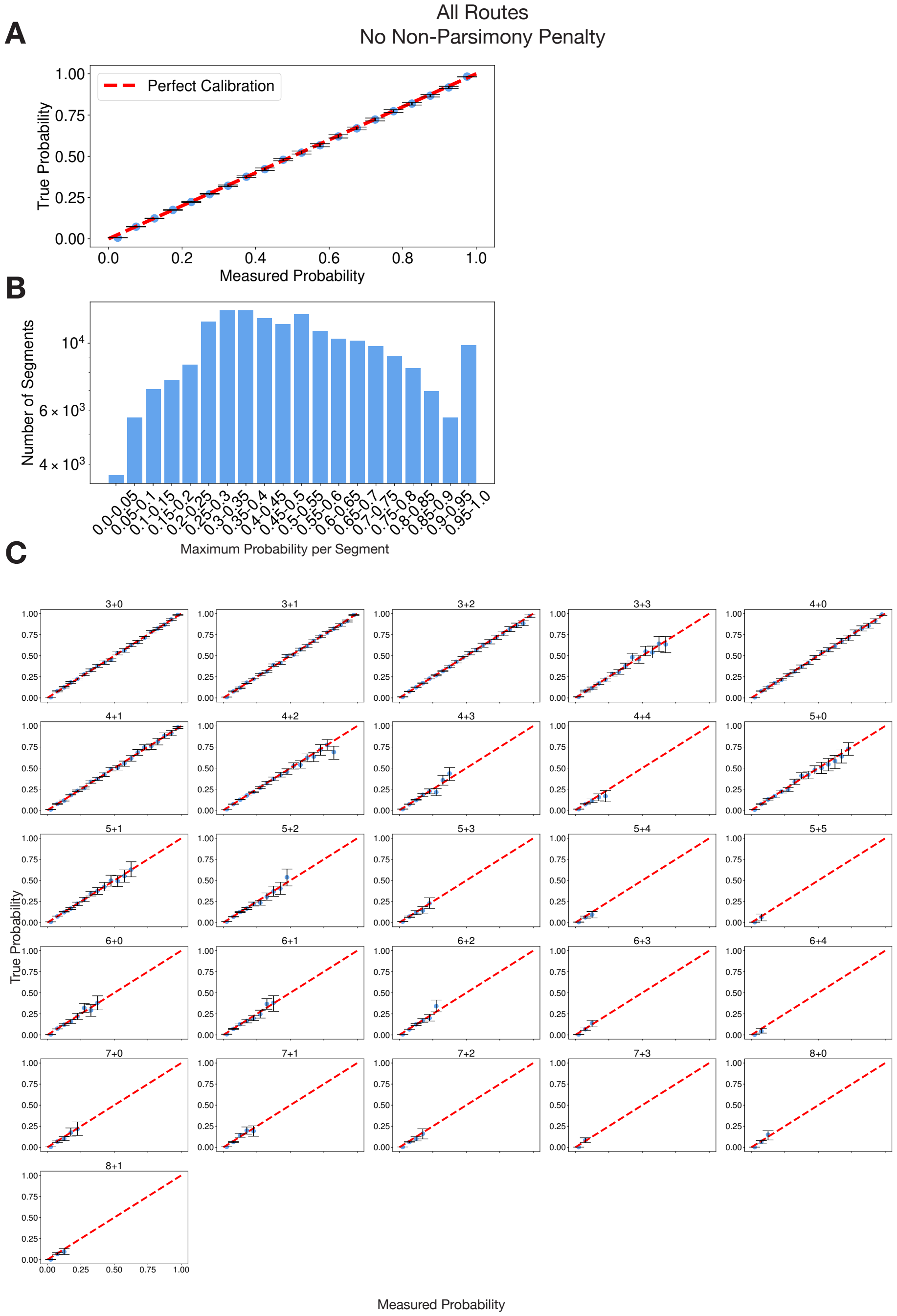

Figure S8: Measuring inferred route probabilities on simulated data. Probability calibration plots for a cohort simulated with all routes and no penalty on non-parsimony applied during inference. **A**, Binned measured probability of different route assignments against true probability calculated as the proportion of segments within each bin of measured probability that have the route assignment corresponding to the probability. 95% confidence intervals were calculated by bootstrapping over samples. **B**, Distribution of the maximum probability across all routes for the segments in the simulated cohort. **C**, Binned measured probability of different route assignments against true probability calculated as the proportion of segments within each bin of measured probability that have the route assignment corresponding to the probability, split by copy number state. 95% confidence intervals were calculated by bootstrapping over samples.

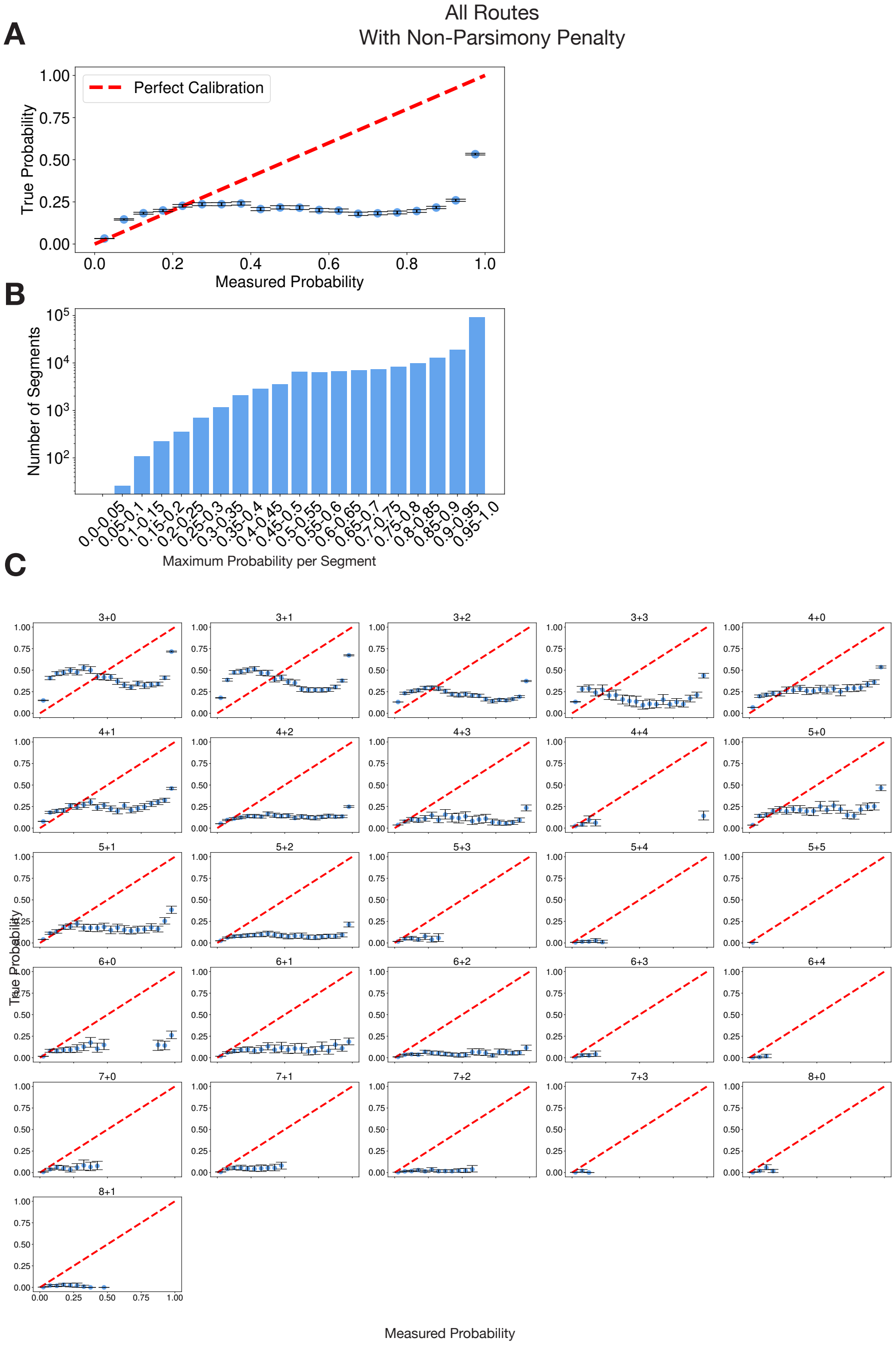

Figure S9: Measuring inferred route probabilities on simulated data. Probability calibration plots for a cohort simulated with all routes and a penalty on non-parsimony applied during inference. **A**, Binned measured probability of different route assignments against true probability calculated as the proportion of segments within each bin of measured probability that have the route assignment corresponding to the probability. 95% confidence intervals were calculated by bootstrapping over samples. **B**, Distribution of the maximum probability across all routes for the segments in the simulated cohort. **C**, Binned measured probability of different route assignments against true probability calculated as the proportion of segments within each bin of measured probability that have the route assignment corresponding to the probability, split by copy number state. 95% confidence intervals were calculated by bootstrapping over samples.

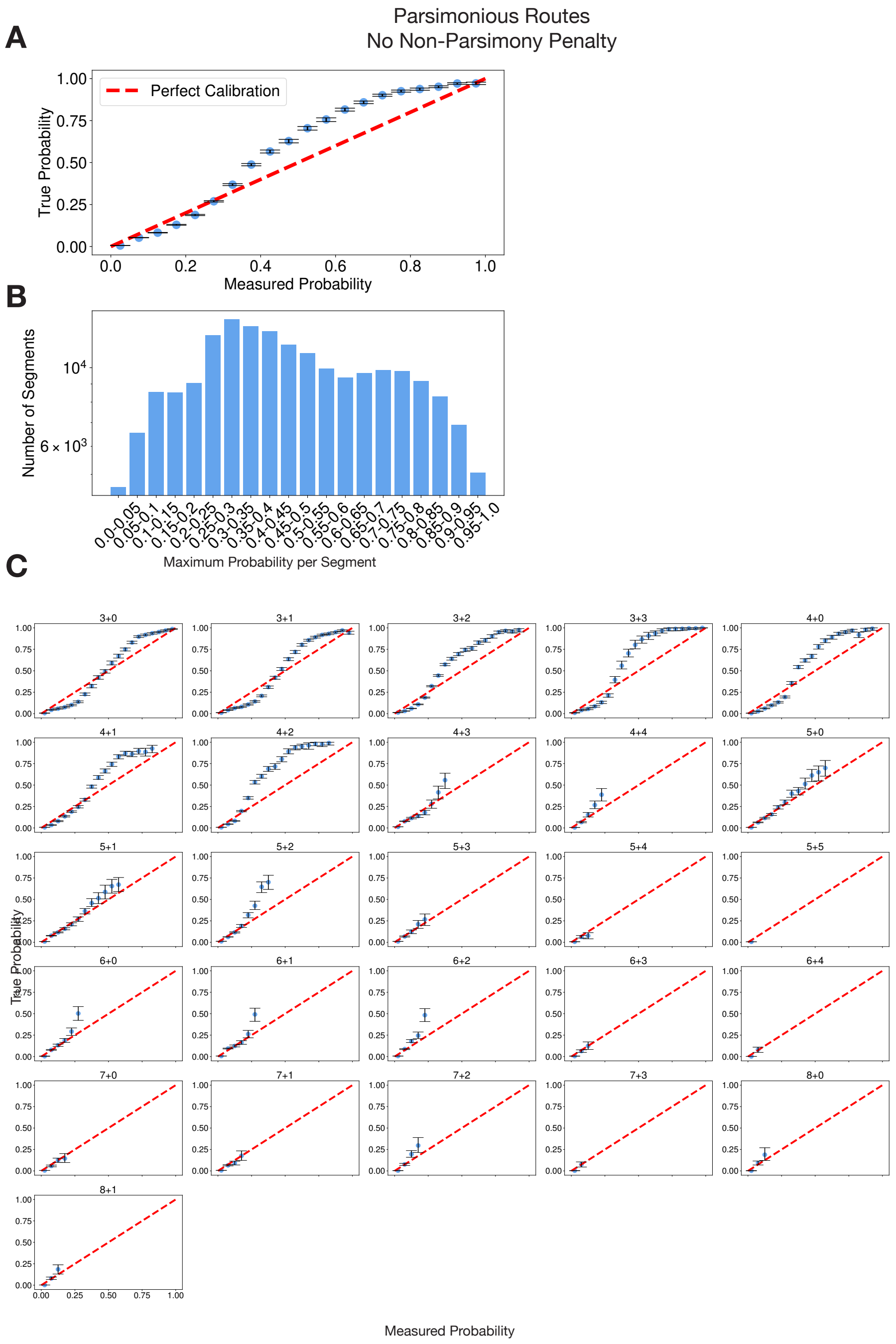

Figure S10: Measuring inferred route probabilities on simulated data. Probability calibration plots for a cohort simulated with parsimonious routes only and no penalty on non-parsimony applied during inference. **A**, Binned measured probability of different route assignments against true probability calculated as the proportion of segments within each bin of measured probability that have the route assignment corresponding to the probability. 95% confidence intervals were calculated by bootstrapping over samples. **B**, Distribution of the maximum probability across all routes for the segments in the simulated cohort. **C**, Binned measured probability of different route assignments against true probability calculated as the proportion of segments within each bin of measured probability that have the route assignment corresponding to the probability, split by copy number state. 95% confidence intervals were calculated by bootstrapping over samples.

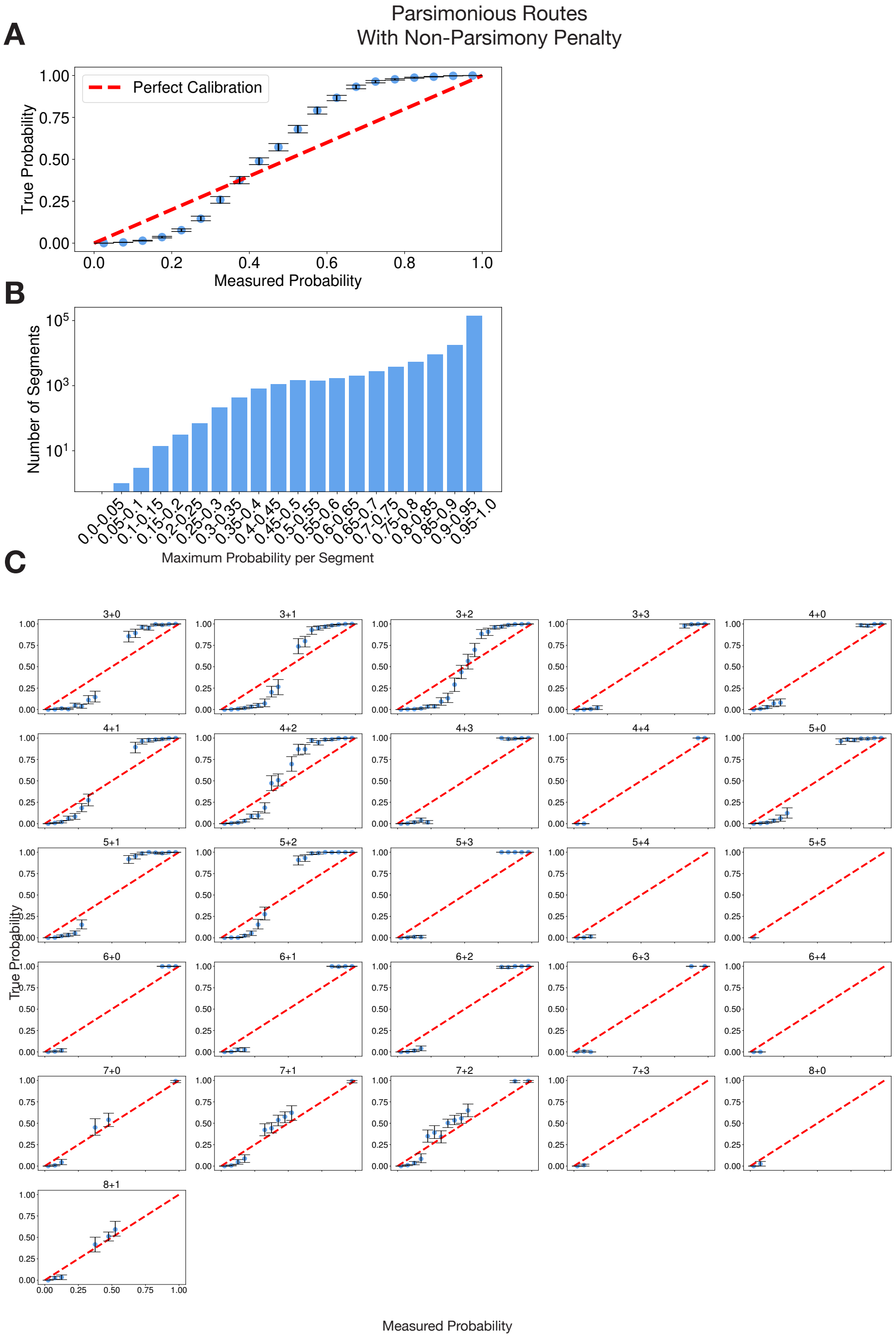

Figure S11: Measuring inferred route probabilities on simulated data. Probability calibration plots for a cohort simulated with parsimonious routes only and a penalty on non-parsimony applied during inference. **A**, Binned measured probability of different route assignments against true probability calculated as the proportion of segments within each bin of measured probability that have the route assignment corresponding to the probability. 95% confidence intervals were calculated by bootstrapping over samples. **B**, Distribution of the maximum probability across all routes for the segments in the simulated cohort. **C**, Binned measured probability of different route assignments against true probability calculated as the proportion of segments within each bin of measured probability that have the route assignment corresponding to the probability, split by copy number state. 95% confidence intervals were calculated by bootstrapping over samples.

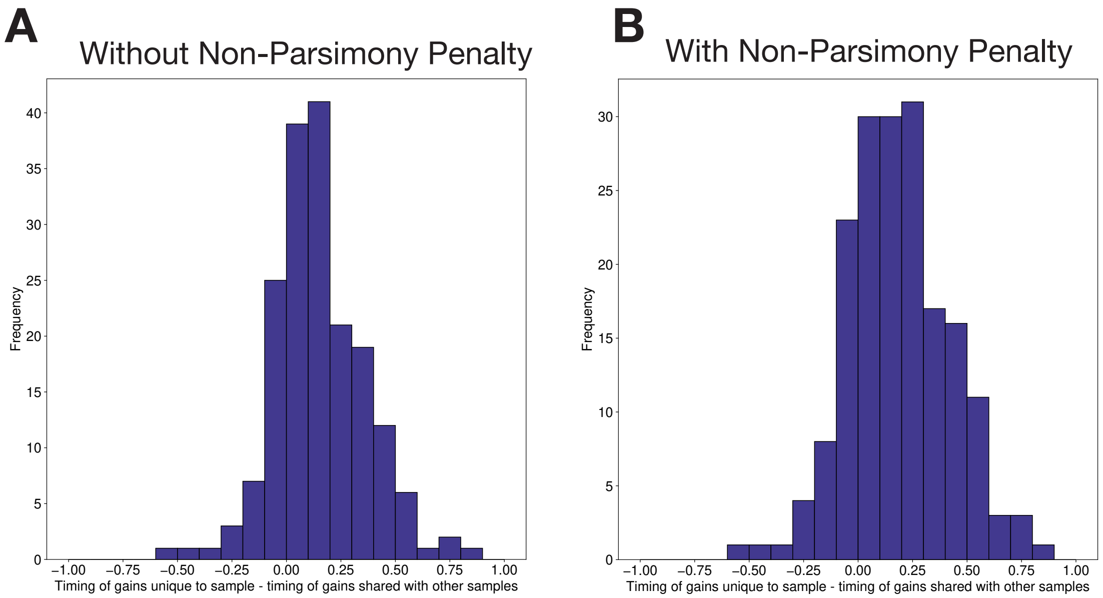

Figure S12: Timing of gains in multi-region tumors. The difference in average first-gain timing of chromosomes uniquely gained in one sample compared to those gained across multiple samples for the same patient in the Hartwig cohort. Measured without (**A**) and with (**B**) a penalty on non-parsimony.

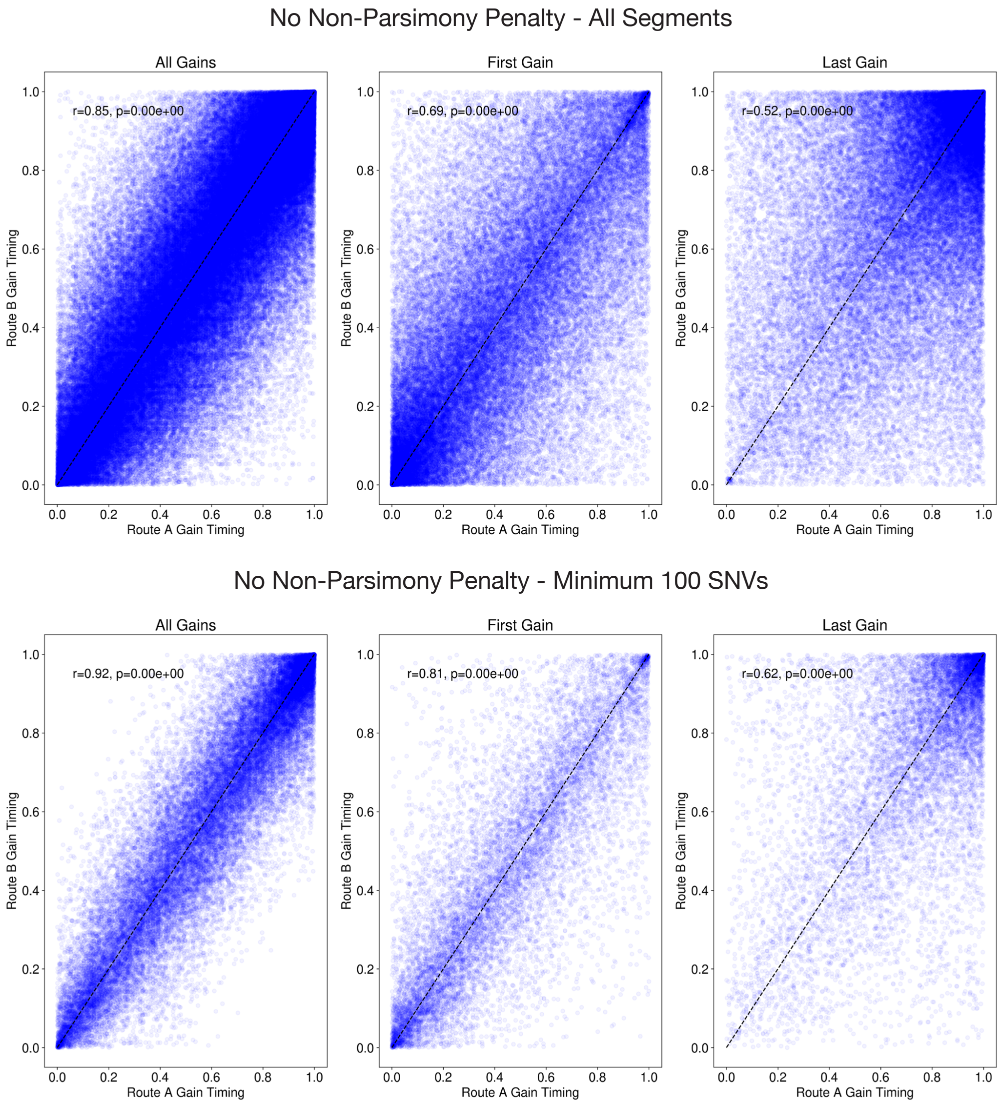

Figure S13: Difference in timing between different gain routes. The timing of gains from different routes in the same sample. Each point represents the timing of a gained segment from the PCAWG and Hartwig cohorts sampled from two different routes from the joint posterior distribution over gain timing and routes. Route A and B are arbitrarily labeled. Only segments with no more than 50% posterior probability on a single gain route are displayed. The timing for all gains, the first gain and last gain per segment are displayed. Comparisons are shown without a penalty on non-parsimony applied, with and without filtering to a minimum of 100 SNVs per gained segment.

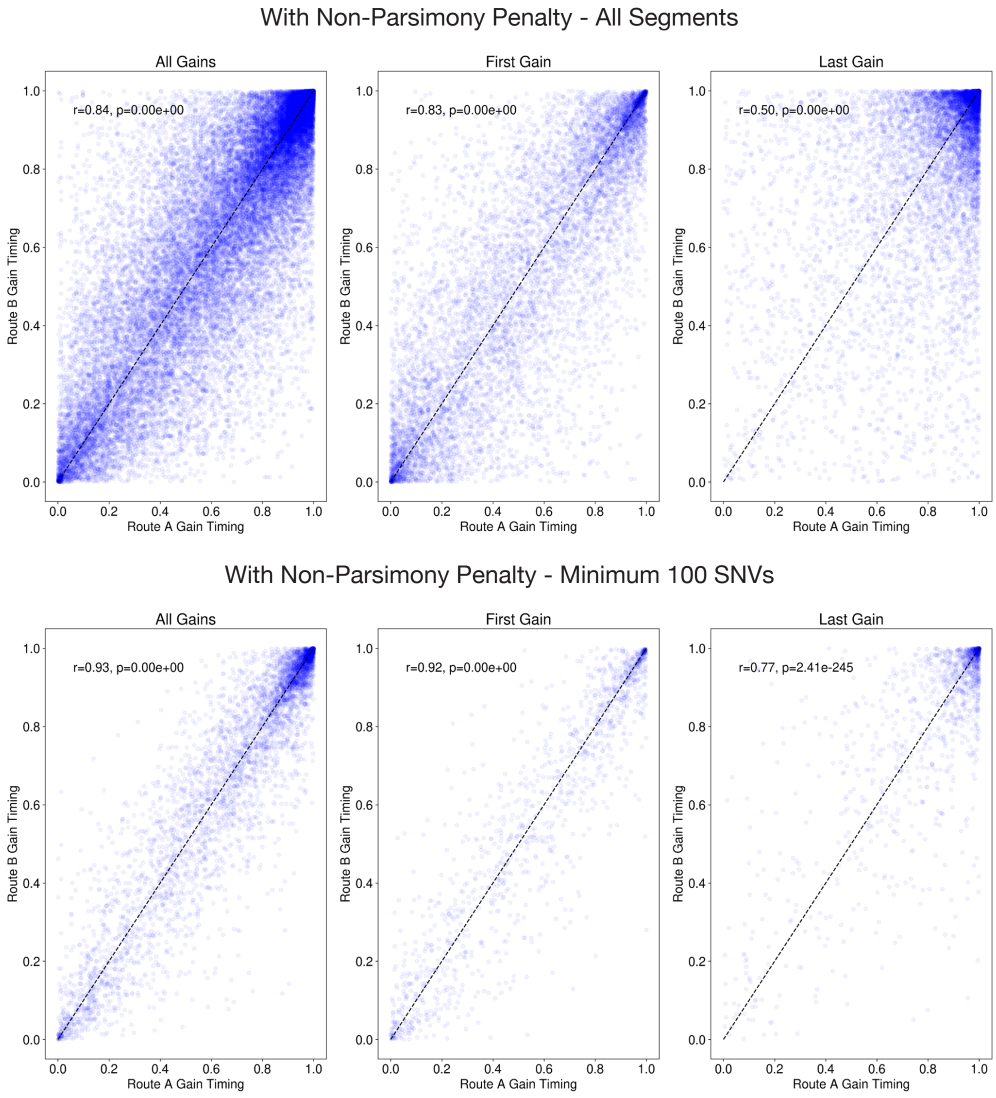

Figure S14: Difference in timing between different gain routes. The timing of gains from different routes in the same sample. Each point represents the timing of a gained segment from the PCAWG and Hartwig cohorts sampled from two different routes from the joint posterior distribution over gain timing and routes. Route A and B are arbitrarily labeled. Only segments with no more than 50% posterior probability on a single gain route are displayed. The timing for all gains, the first gain and last gain per segment are displayed. Comparisons are shown with a penalty on non-parsimony applied, with and without filtering to a minimum of 100 SNVs per gained segment.

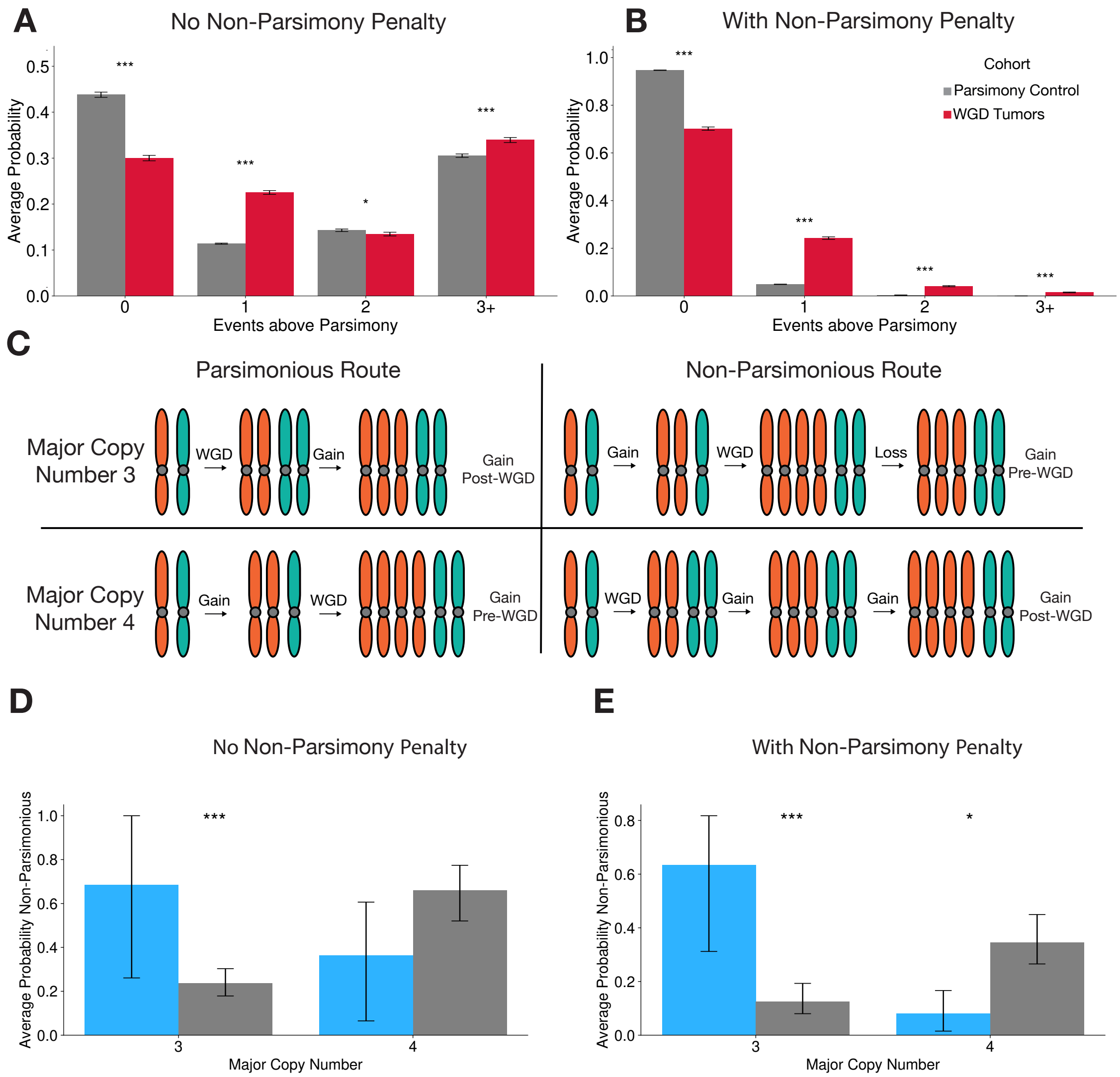

Figure S15: Non-parsimony in copy number gain evolution. **A**,**B**, The average posterior probability on the number of additional events required to reach the final state over the most parsimonious route for complex gained states in the PCAWG and Hartwig cohort compared to a simulated control where only parsimonious routes were included. Measured without (**A**) and with a penalty (**B**) on non-parsimony during inference. Statistical significance is calculated using a permutation test and 95% confidence intervals by bootstrapping over samples. **C**, Schematic demonstrating that parsimonious routes have an earlier independent gain for major copy number three copy number segments in WGD tumors and vice versa for major copy number four segments. **D**-**E**, The average probability on non-parsimonious routes for gained segments in kidney renal cell carcinoma split by major copy number and gain location without (**D**) and with a penalty (**E**) on non-parsimony during inference. Statistical significance is calculated using a permutation test and 95% confidence intervals by bootstrapping over samples. * and *** indicate comparisons where $p < 0.05$ and < 0.001 respectively.

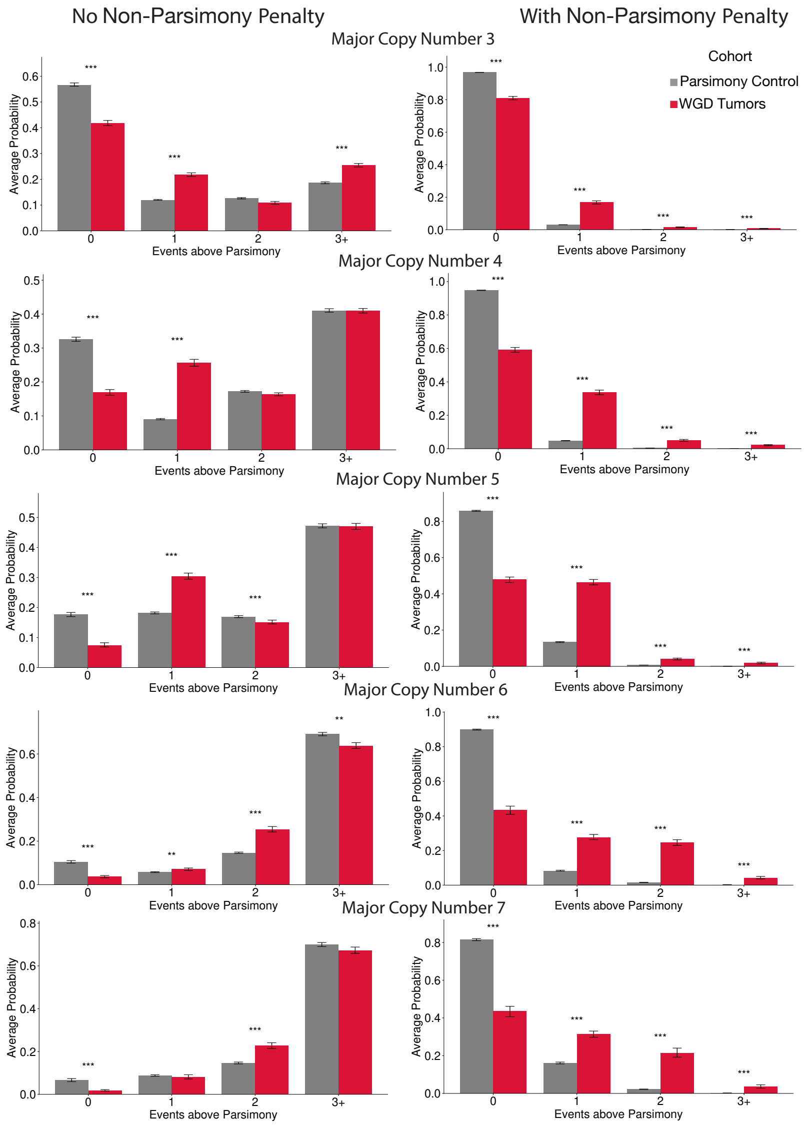

Figure S16: Non-parsimony by copy number state. The average posterior probability on the number of additional events required to reach the final state over the most parsimonious route for complex gained states in the PCAWG and Hartwig cohort compared to a simulated control where only parsimonious routes were included, split by major copy number state. Measured without and with a penalty on non-parsimony during inference. Statistical significance is calculated using a permutation test and 95% confidence intervals by bootstrapping over samples. *, ** and *** indicate comparisons where $p < 0.05$, < 0.01 and < 0.001 respectively.

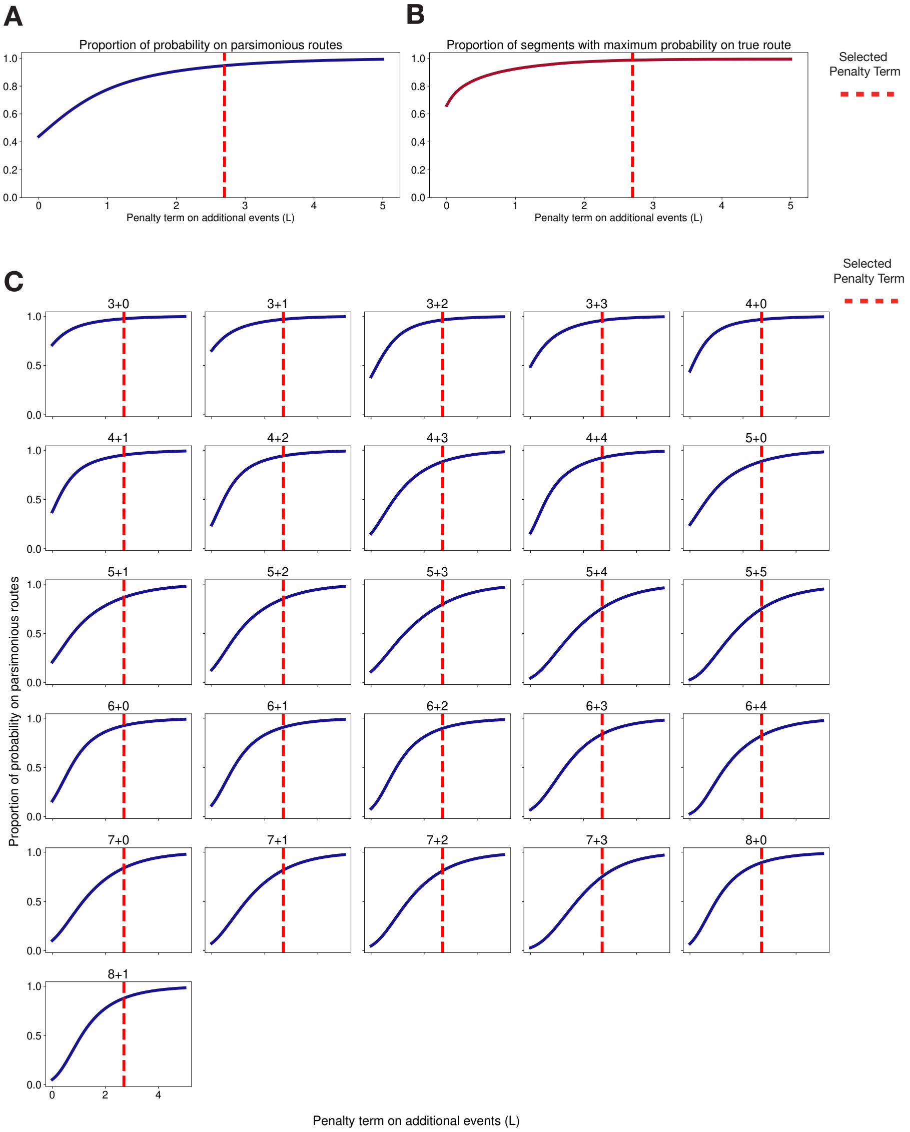

Figure S17: Calibrating a penalty on non-parsimony. **A**, The average probability assigned to parsimonious routes on a representative simulated cohort of parsimonious only copy number routes against a non-parsimony penalty term. The non-parsimony penalty is $e^{(-NL)}$ where N is the number of extra events over the minimum number of possible events in all possible routes for the segment. **B**, The average number of segments with maximum posterior probability assigned to parsimonious routes on a representative simulated cohort of parsimonious only copy number routes against a non-parsimony penalty term. **C**, The average probability assigned to parsimonious routes on a representative simulated cohort of parsimonious only copy number routes against a non-parsimony penalty term, split by copy number state.

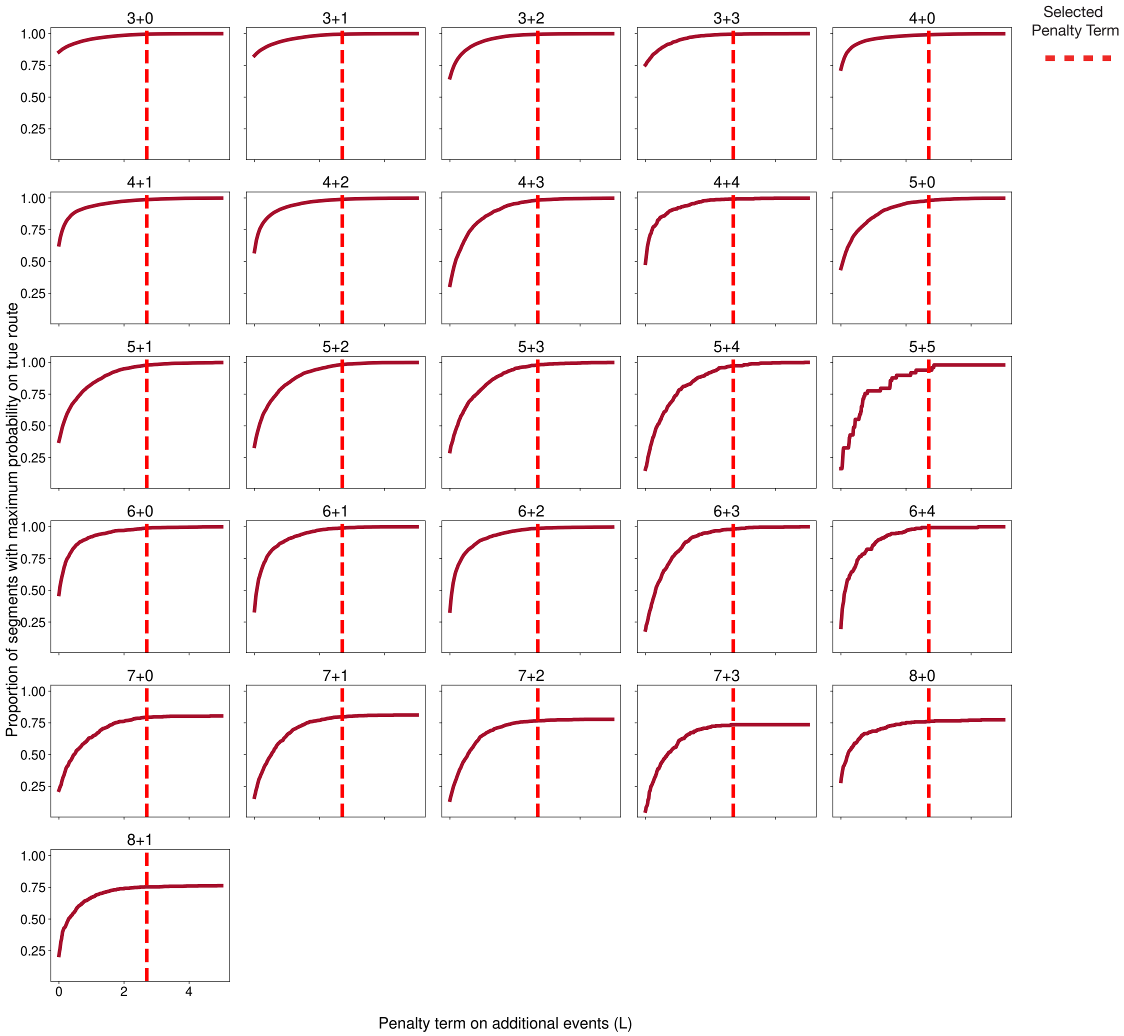

Figure S18: Calibrating a penalty on non-parsimony. The average number of segments with maximum posterior probability assigned to parsimonious routes on a representative simulated cohort of parsimonious only copy number routes against a non-parsimony penalty term, split by copy number state. The non-parsimony penalty is $e^{(-NL)}$ where N is the number of extra events over the minimum number of possible events in all possible routes for the segment.

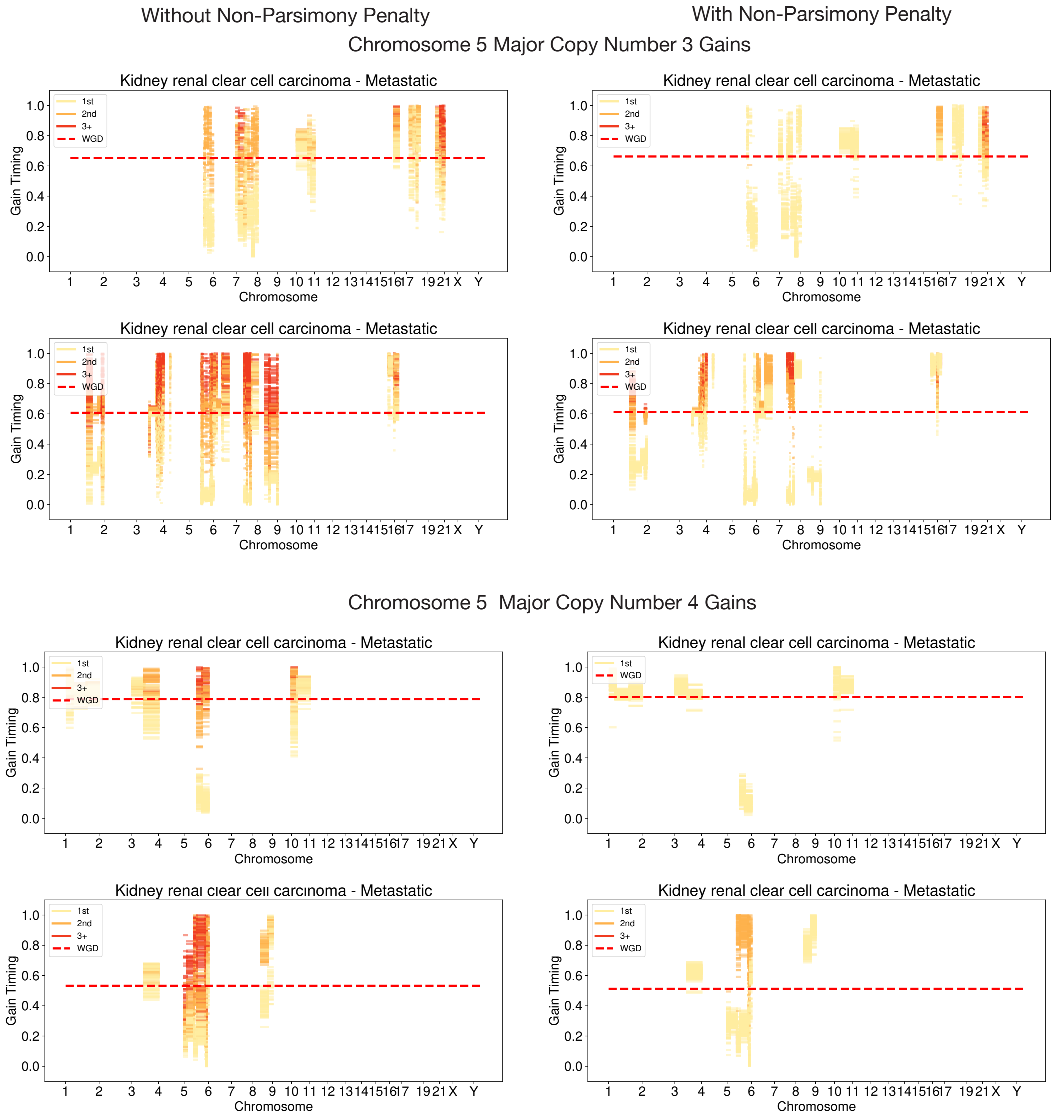

Figure S19: Clear-cell sample gain timing. The posterior distribution over copy number gain timing for a selection of clear-cell renal cell carcinomas with and without a penalty on non-parsimony. Samples are split based on the major copy number of the gains on chromosome 5.

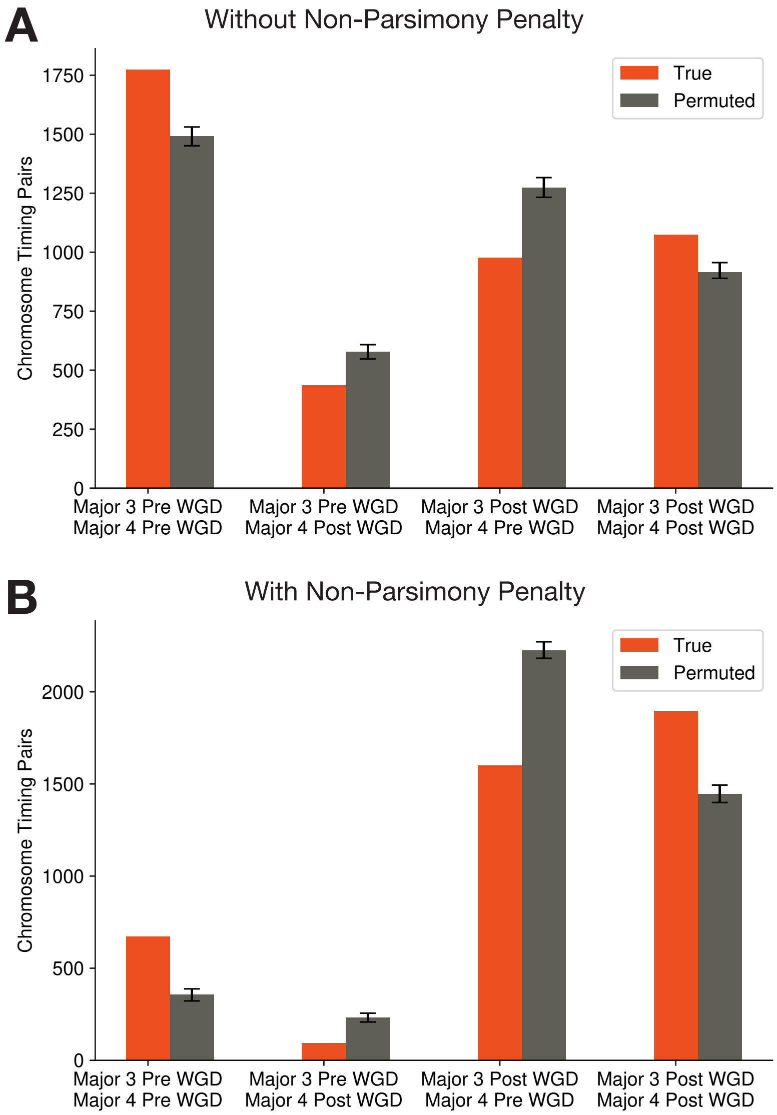

Figure S20: Agreement between gain routes of segments on the same chromosome. The number of pairs of segments with different major copy number on the same chromosome split by the route history of each segment in the PCAWG and Hartwig cohort. The permutation model is formed by permuting route histories between segments with the same copy number state within each sample. Measured without (**A**) and with (**B**) a penalty on non-parsimony.

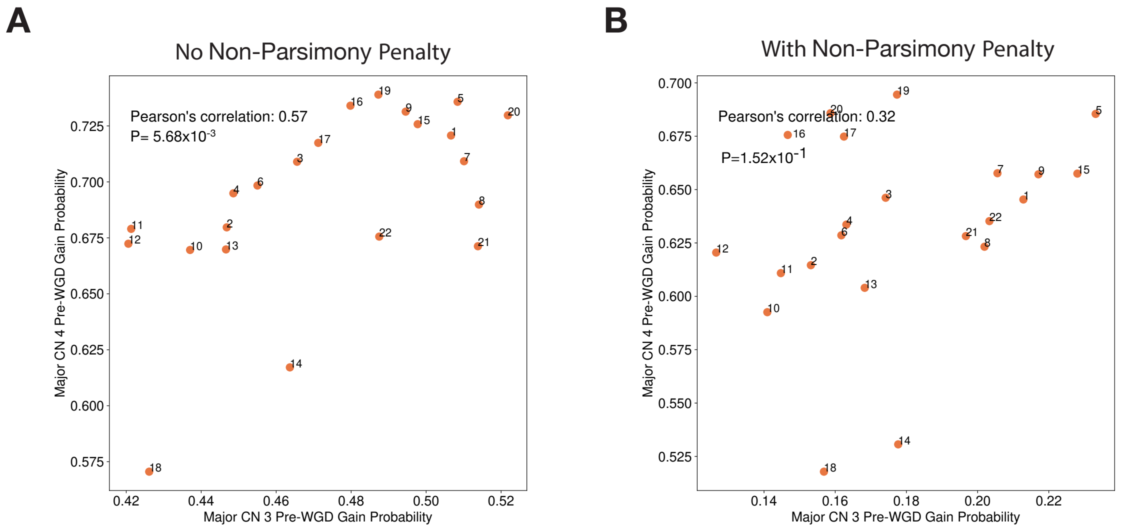

Figure S21: Probability of pre-WGD gains in different chromosomes and copy number states. A,B The average probability that the first gain arises pre-WGD in different chromosomes in major copy number 3 and 4 states as measured in the PCAWG and Hartwig datasets. Measured without (**A**) and with (**B**) a penalty on non-parsimony.

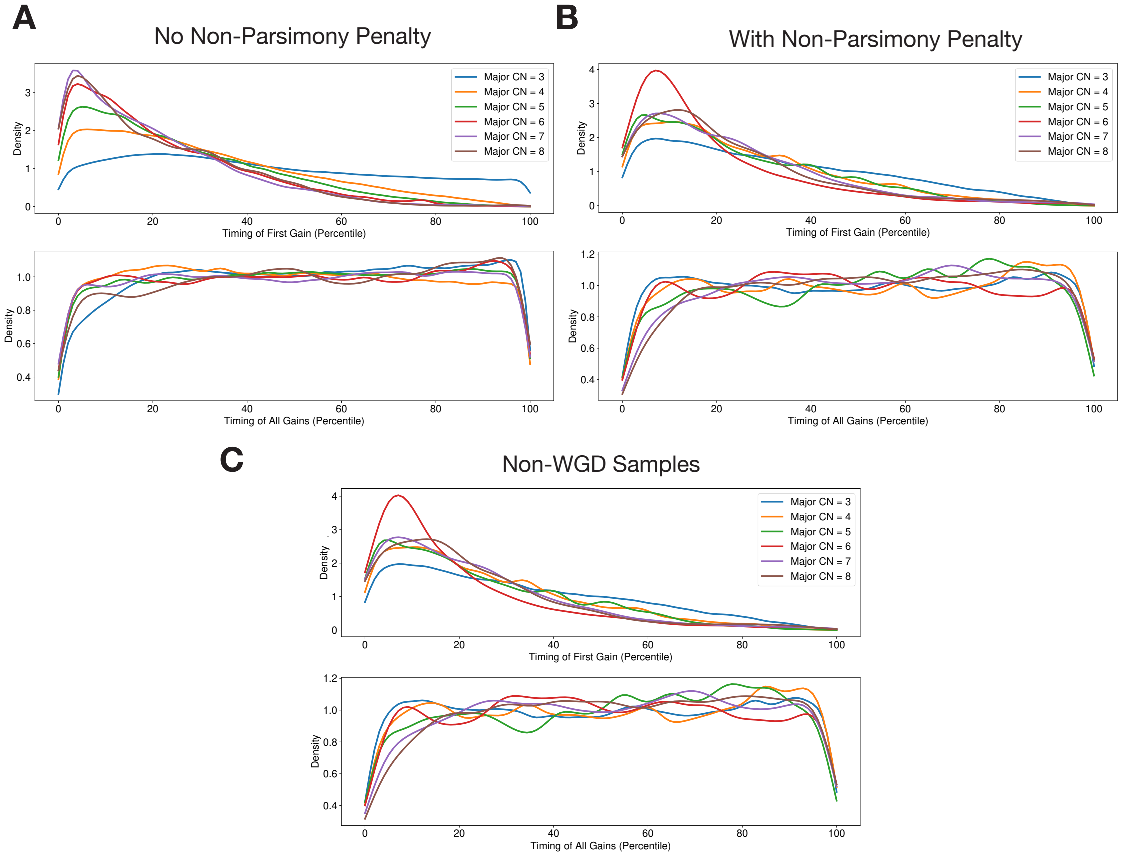

Figure S22: Distribution of gain timing by major copy number. A-C. The distribution of the percentile timing within samples of the first gains and all gains in complex segments, as defined by their quantile ranking within each sample, split by major copy number, without (**A**) and with a penalty (**B**) on non-parsimony during inference in WGD samples and (**C**) in non-WGD samples.

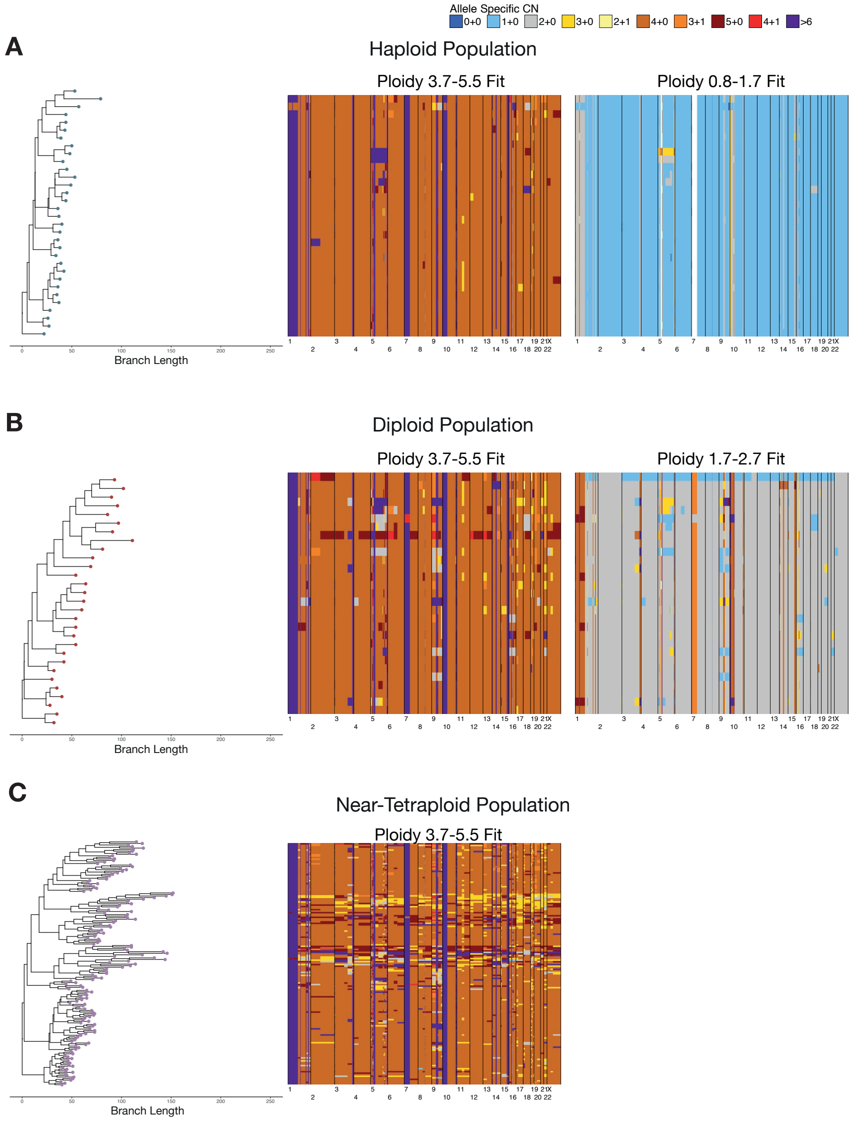

Figure S23: Single copy number profiles of an undifferentiated sarcoma. Single-cell copy number phylogenies and associated allele-specific copy number profiles from an individual undifferentiated sarcoma. These are split by different ploidy populations within the same tumor (**A**-**C**). One set of copy number profiles is fitted to true tumor cell ploidy as determined by fluorescence-activated cell sorting, the second is fitted to a near-tetraploid state to get an unbiased estimate of copy number heterogeneity.

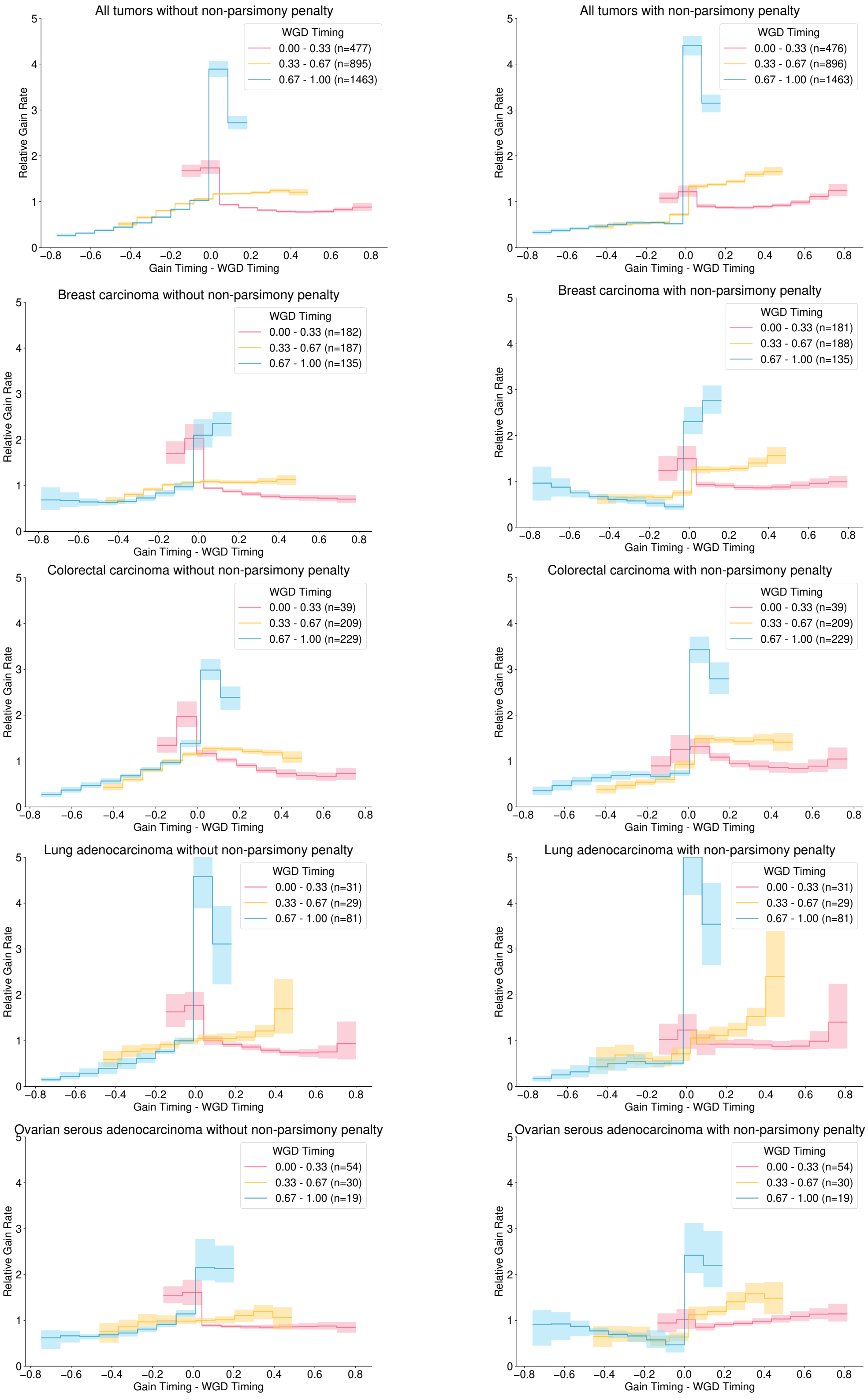

Figure S24: Distribution of gain rates relative to WGD by cancer type. The normalized rate of gains relative to WGD timing for a selection of cancer types with and without a penalty on non-parsimony applied during inference. 95% confidence intervals are calculated by bootstrapping over samples.

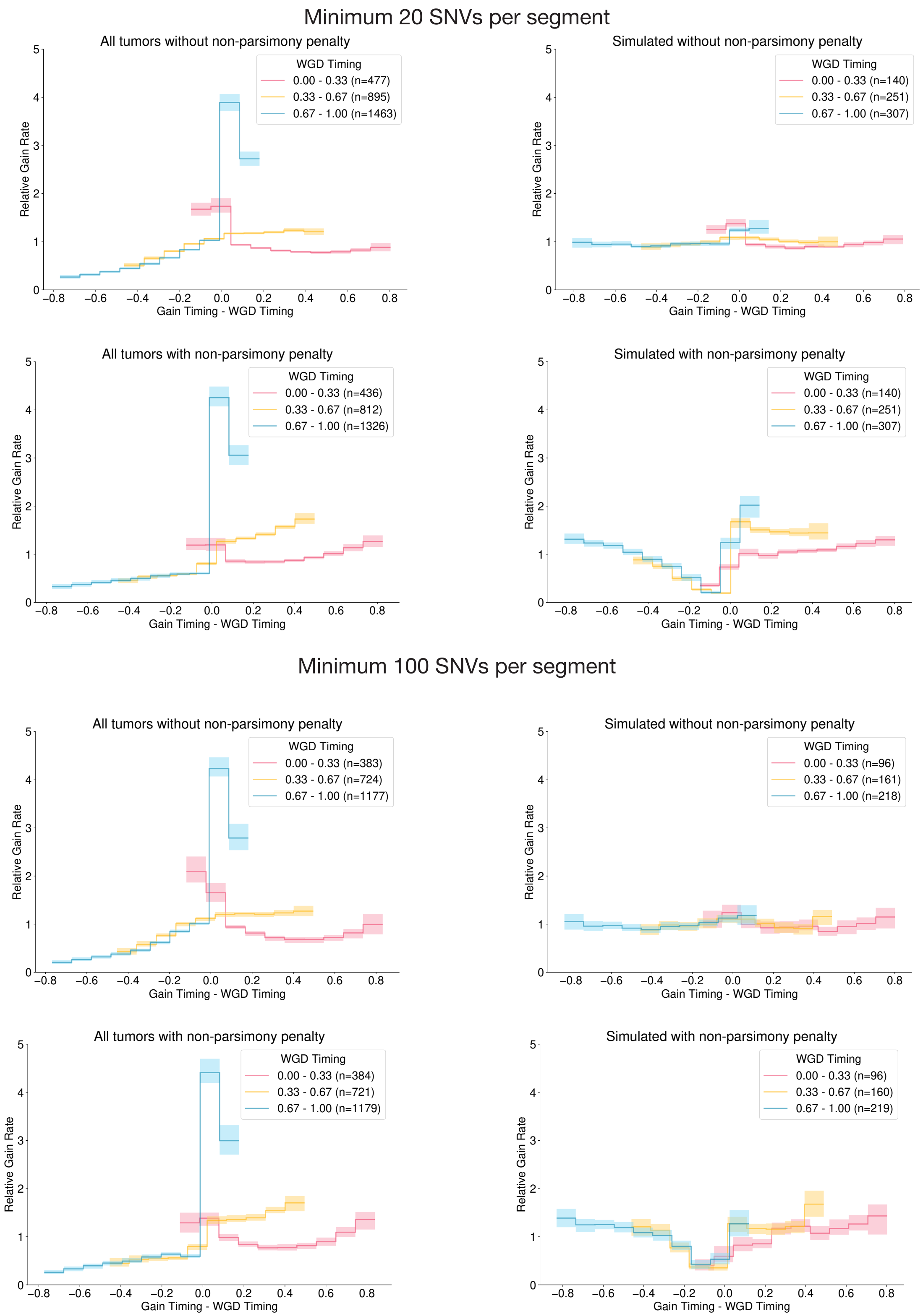

Figure S25: Distribution of gain rates relative to WGD compared to simulations. The normalized rate of gains relative to WGD timing for the PCAWG and Hartwig cohort and a cohort simulated to have a uniform rate of gains relative to the WGD. Applied with and without a penalty on non-parsimony during inference. 95% confidence intervals are calculated by bootstrapping over samples.

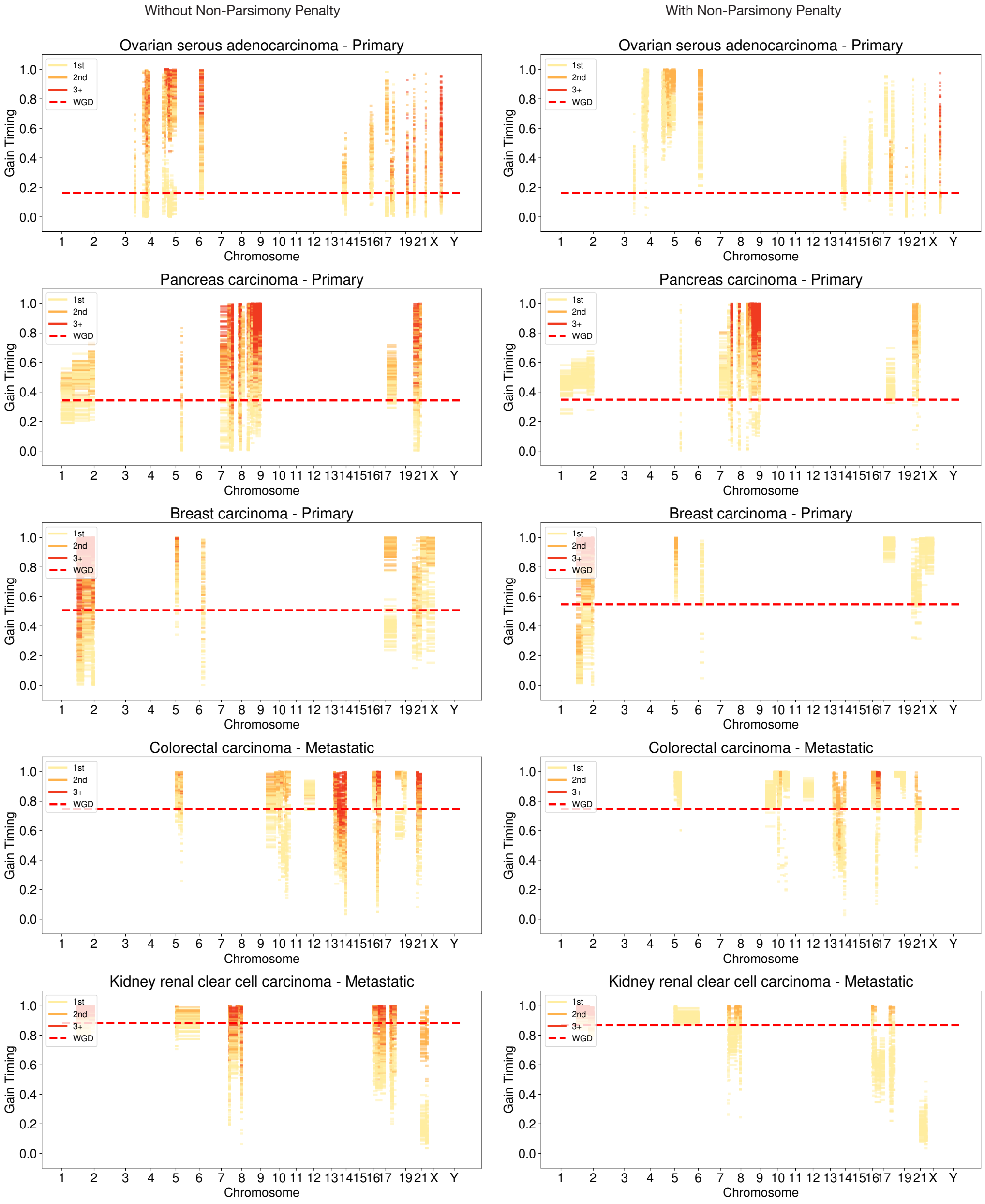

Figure S26: Example sample gain timing. The posterior gain timing distribution for a number of genome duplicated tumors in the PCAWG and Hartwig cohort, with and without a penalty on non-parsimony applied.

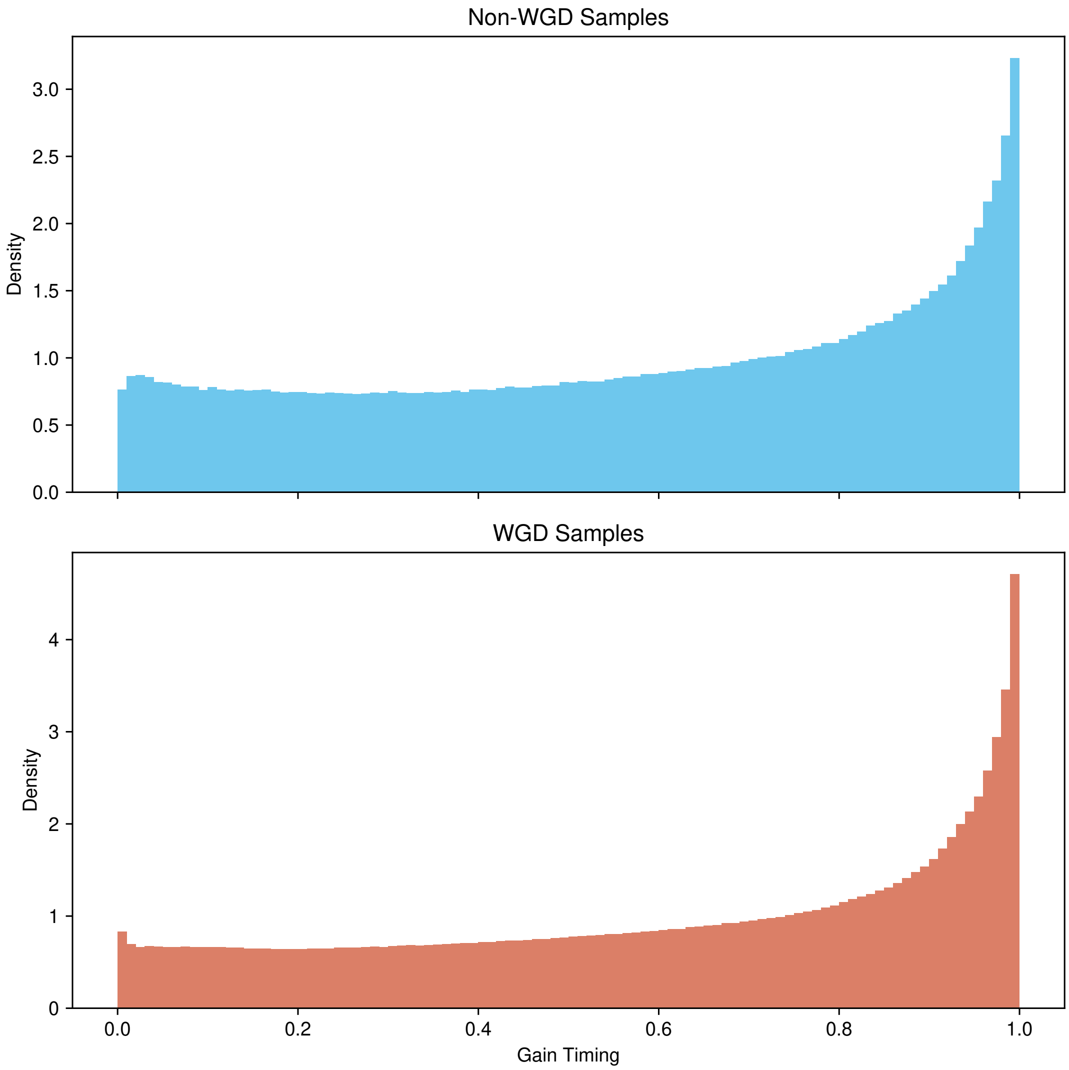

Figure S27: Combined distribution over gain timing by WGD status. Distribution of gain timing across all segments and samples for non-WGD and WGD samples. Inference without penalty on non-parsimony.

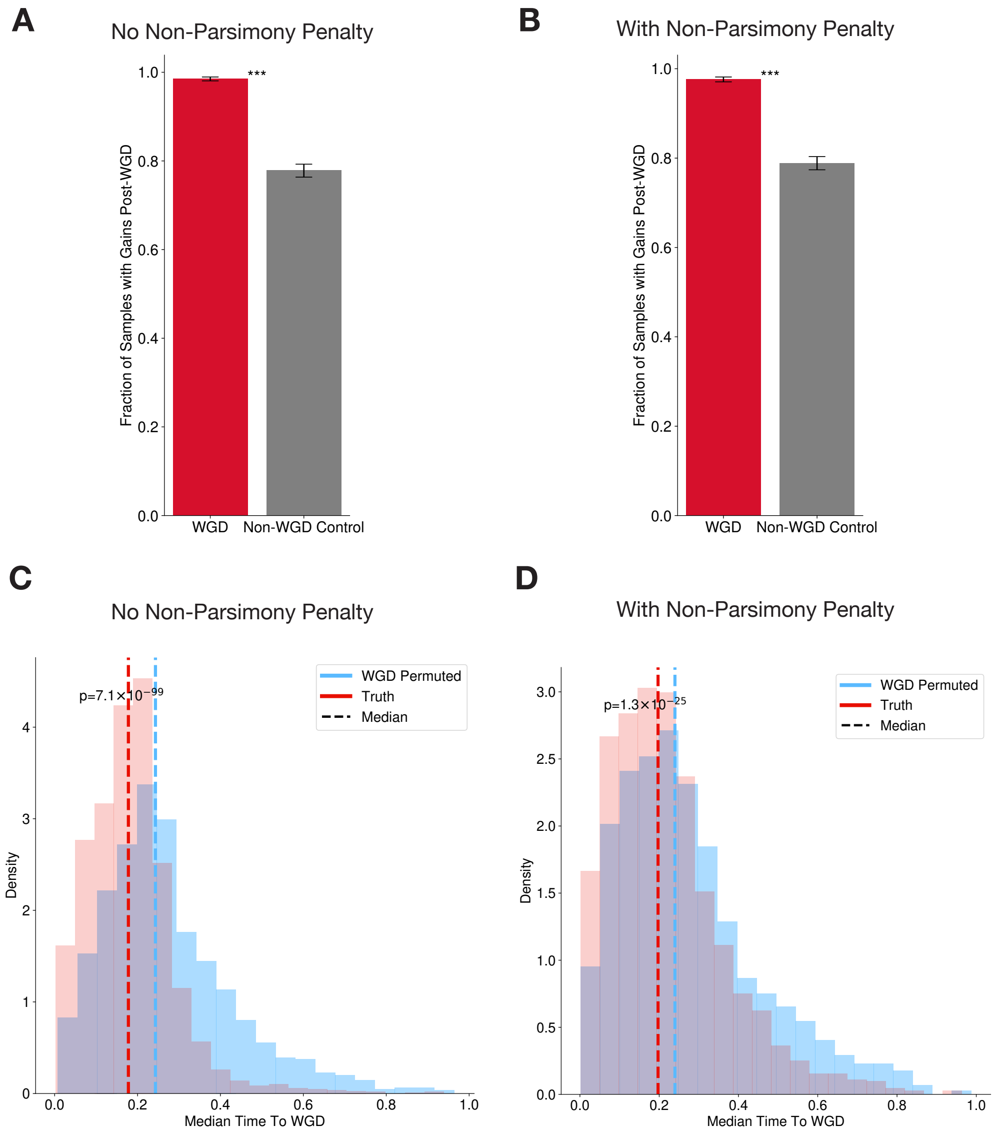

Figure S28: The timing of gains relative to WGD. A,B Proportion of samples with gains post-WGD for WGD tumors and a cohort of control non-WGD tumors with a pseudo-WGD timing randomly sampled from WGD tumors with the same cancer type without (**A**) and with (**B**) a penalty on non-parsimony during inference. Statistical significance is calculated with a permutation test and 95% confidence intervals by bootstrapping over samples **C,D** Distribution of the median mutation time between the distribution of the timing of all independent gains and the median WGD timing for each sample for the correct WGD timing and a cohort where WGD timing is permuted between samples of the same cancer type. Applied without (**C**) and with (**D**) a penalty on non-parsimony during inference. Statistical significance was calculated by Mann Whitney U test. *** indicates a comparison where $p < 0.001$.

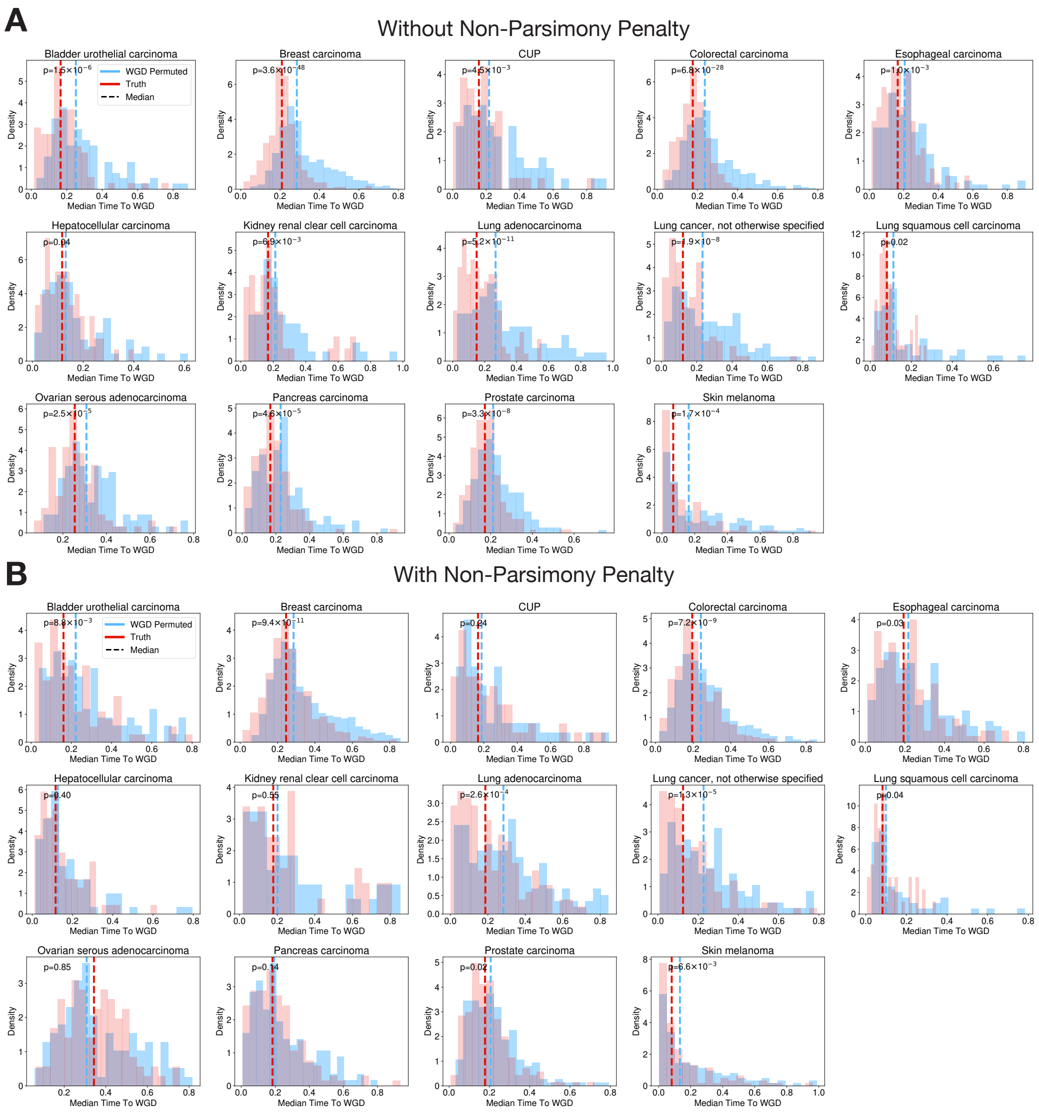

Figure S29: The timing of gains relative to WGD by cancer type. **A**,**B** Distribution of the median mutation time between the distribution of the timing of all independent gains and the median WGD timing for each sample for the correct WGD timing and a cohort where WGD timing is permuted between samples of the same cancer type. Split by cancer type. Measured without (**A**) and with (**B**) a penalty on non-parsimony during inference. Statistical significance was calculated by Mann Whitney U test.

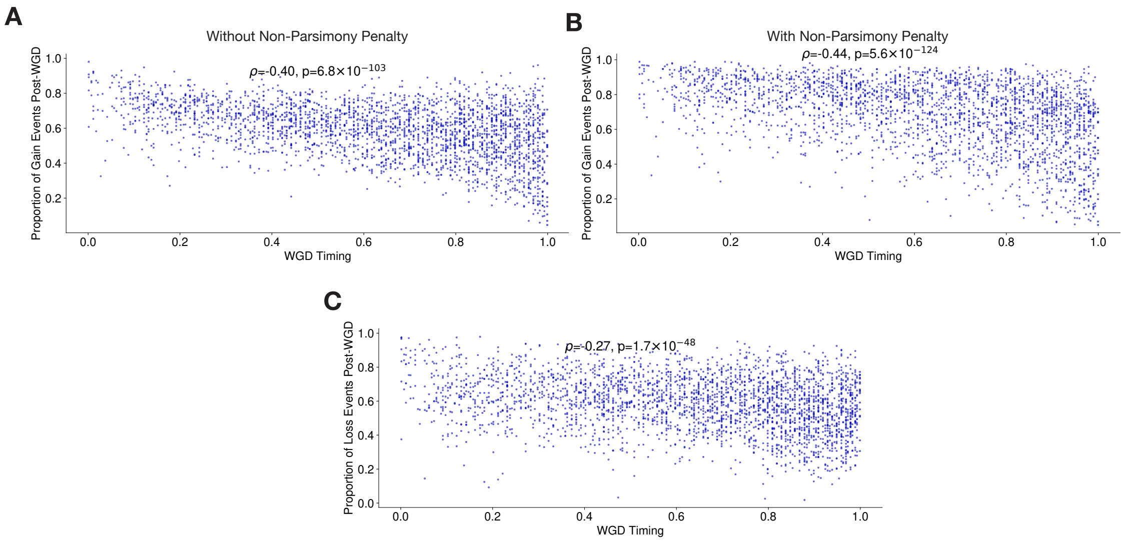

Figure S30: Proportion of copy number events post-WGD. The proportion of copy number segments per sample identified as occurring post-WGD for gains measured without a penalty on non-parsimony (**A**), gains with a penalty on non-parsimony (**B**) and for loss events (**C**). Correlation coefficient measured by Spearman's. For tumors with early WGD, most gains and losses occur after the genome duplication. However, the proportion of gains post-WGD for samples with a late WGD has a much wider range, likely reflective of increased instability post-WGD.

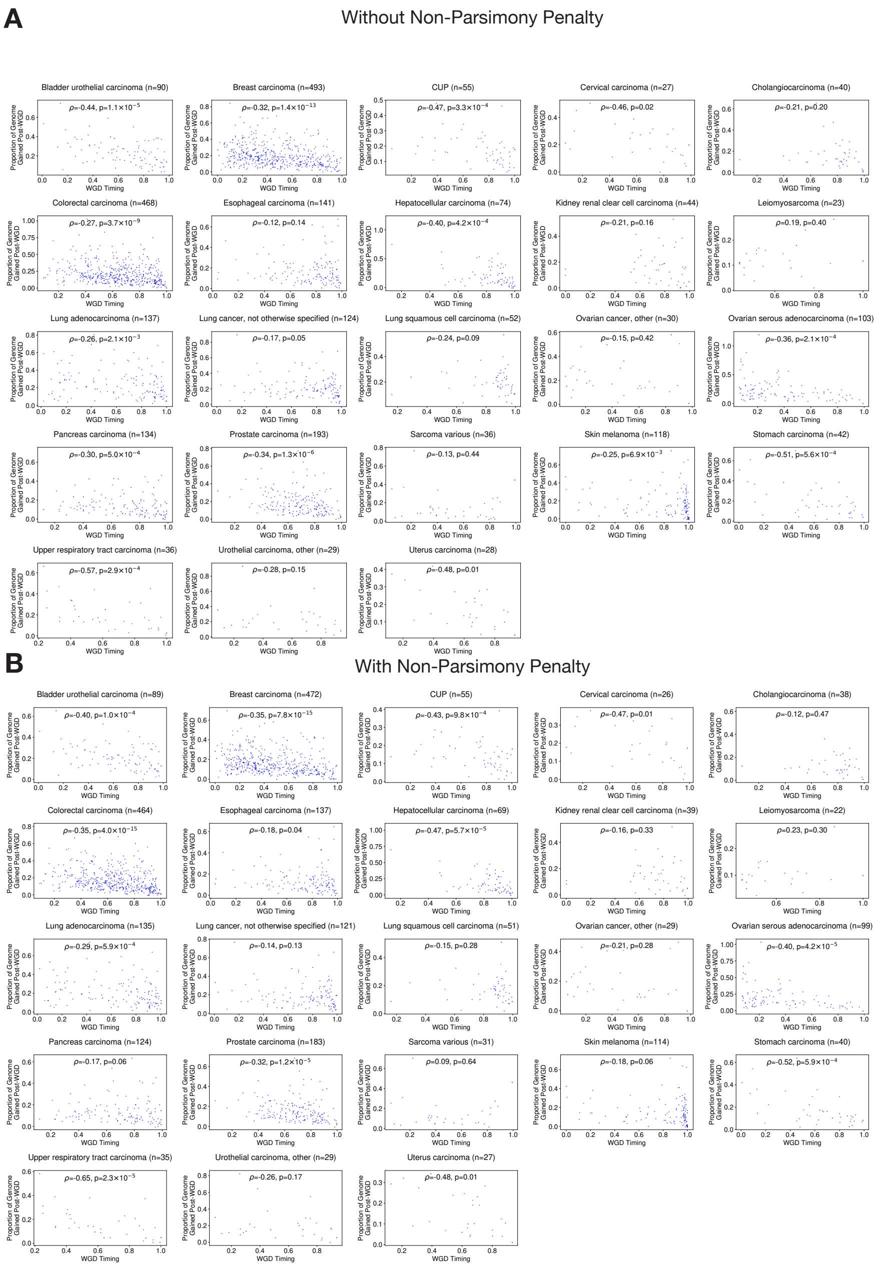

Figure S31: The relationship between genome gained post-WGD and WGD timing by cancer type. Proportion of genome gained after the WGD against WGD timing for WGD tumors in PCAWG and Hartwig. Measured without (**A**) and with (**B**) a penalty on non-parsimony during inference. Correlation coefficient measured by Spearman's.

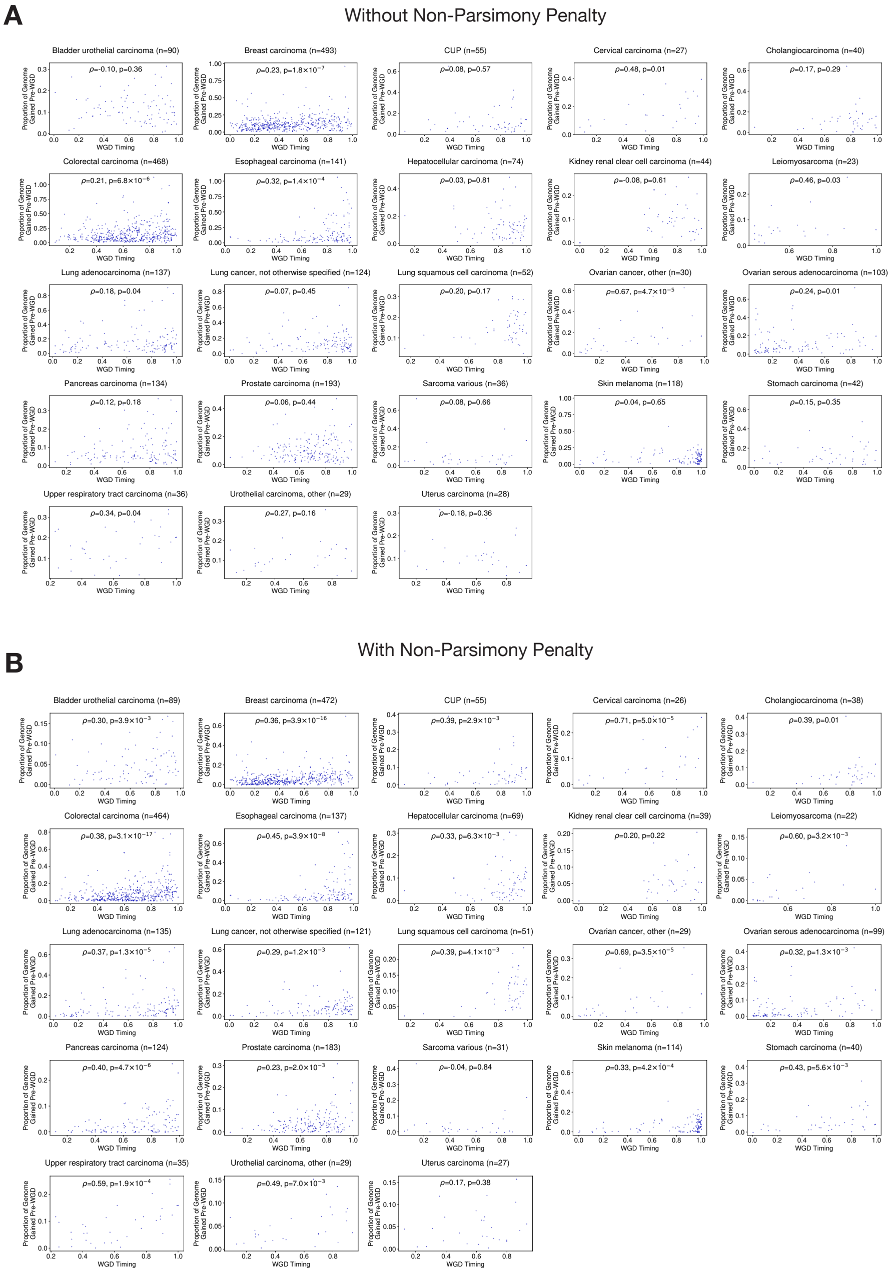

Figure S32: The relationship between genome gained pre-WGD and WGD timing by cancer type. Proportion of genome gained before the WGD against WGD timing for WGD tumors in PCAWG and Hartwig. Measured without (**A**) and with (**B**) a penalty on non-parsimony during inference. Correlation coefficient measured by Spearman's.

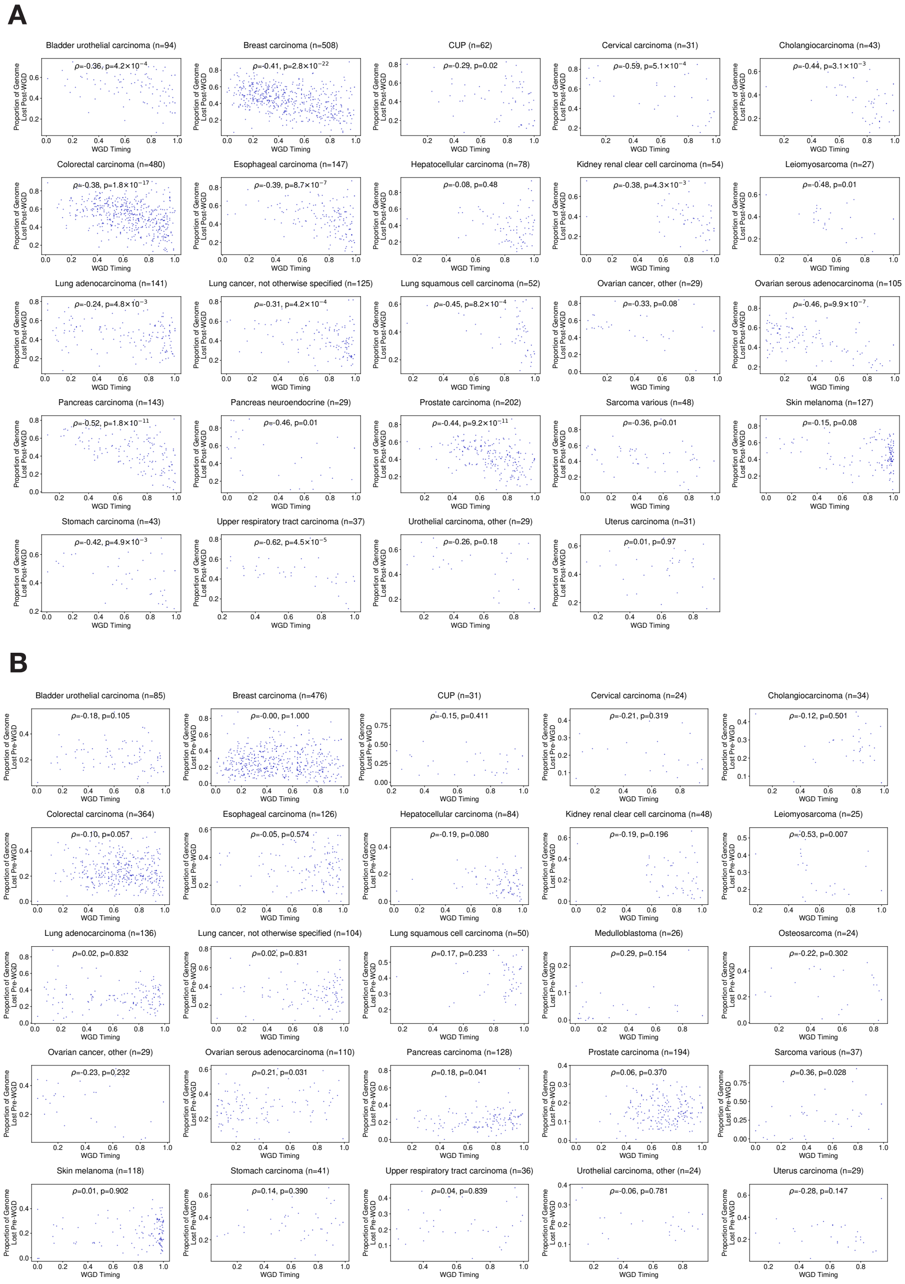

Figure S33: The relationship between fraction of genome lost pre and post-WGD and WGD timing by cancer type. The proportion of genome lost post-WGD (**A**) and pre-WGD (**B**) against WGD timing for WGD tumors in PCAWG and Hartwig. Correlation coefficient measured by Spearman's.

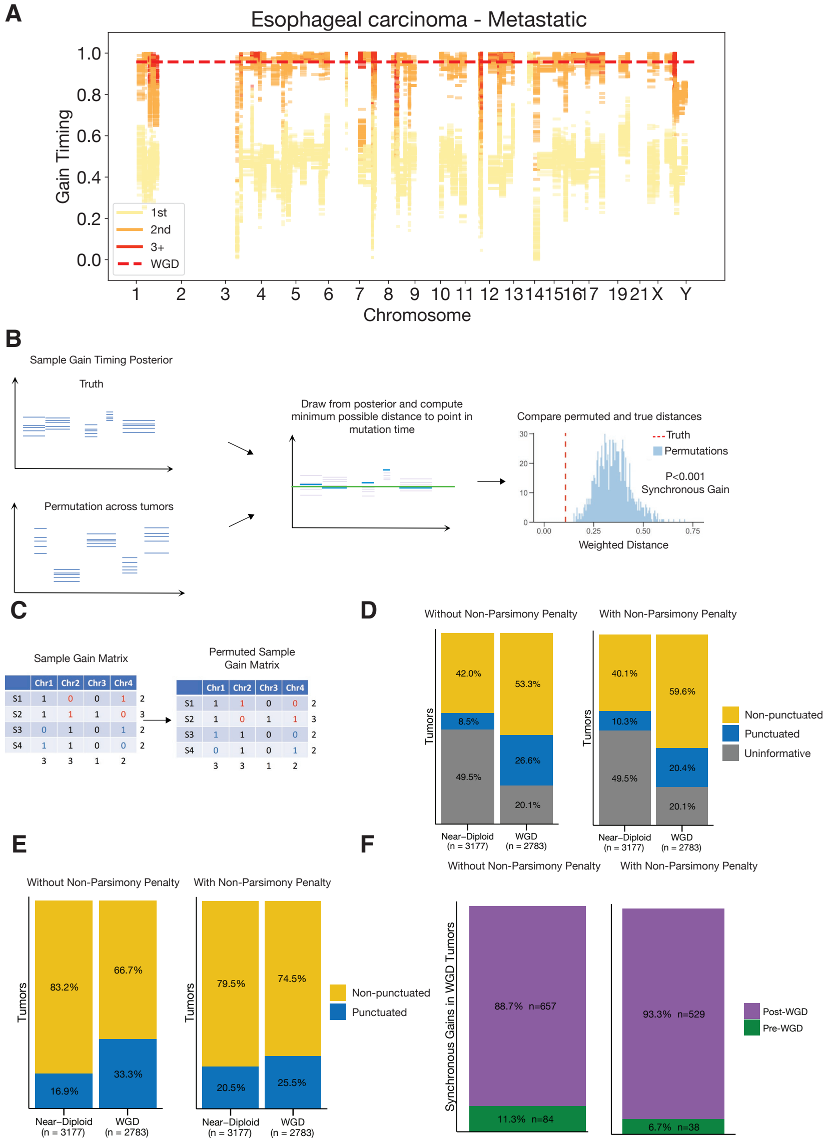

Figure S34: Punctuated gains in WGD tumors. **A**, Example posterior distribution of copy number gain timing in a genome duplicated sample with a punctuated burst of gains. **B**, Schematic of procedure to identify samples with copy number gains occurring over a significantly shorter time period than expected under a permutation model. **C**, Schematic of a permutation scheme that maintains the number of gain chromosomes per sample and the number of times a chromosome is gained across the cohort. **D**, The proportion of tumors that have clonal gains identified as occurring in a punctuated burst, split by WGD status, with and without a penalty on non-parsimony. Uninformative samples are defined as those where the number of gains was too low to classify. **E**, The proportion of tumors that have clonal gains identified as occurring in a punctuated burst as in **D**, with uninformative samples removed. **F**, The proportion of punctuated gains occurring in WGD samples classified by whether they occurred pre- or post-WGD, with and without a penalty on non-parsimony.

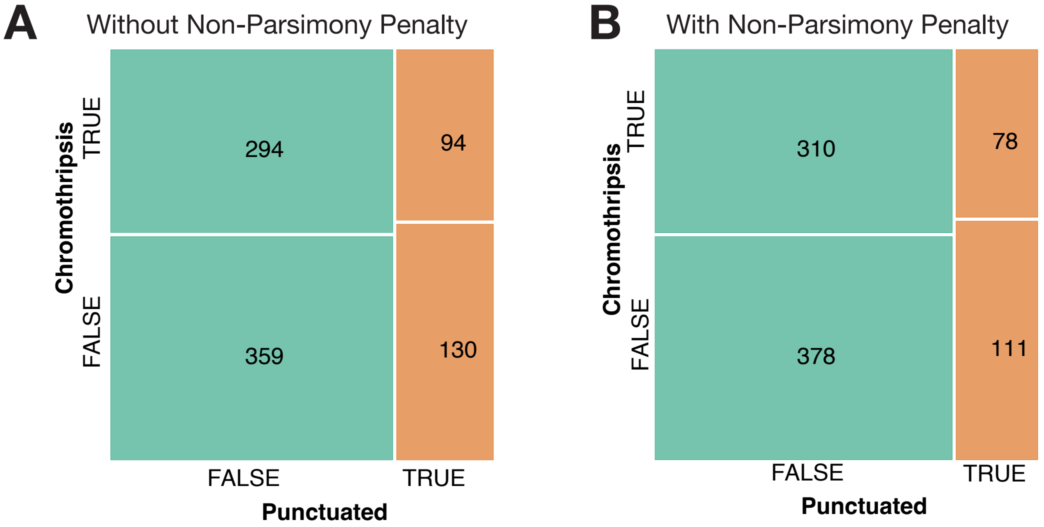

Figure S35: Association between chromothripsis and punctuated gains. The number of samples split by their chromothripsis and punctuated gain status in the PCAWG cohort. Chromothripsis calls provided by the PCAWG consortium. Measured without (**A**) and with (**B**) a penalty on non-parsimony. No association between chromothripsis and punctuated gain status was observed.

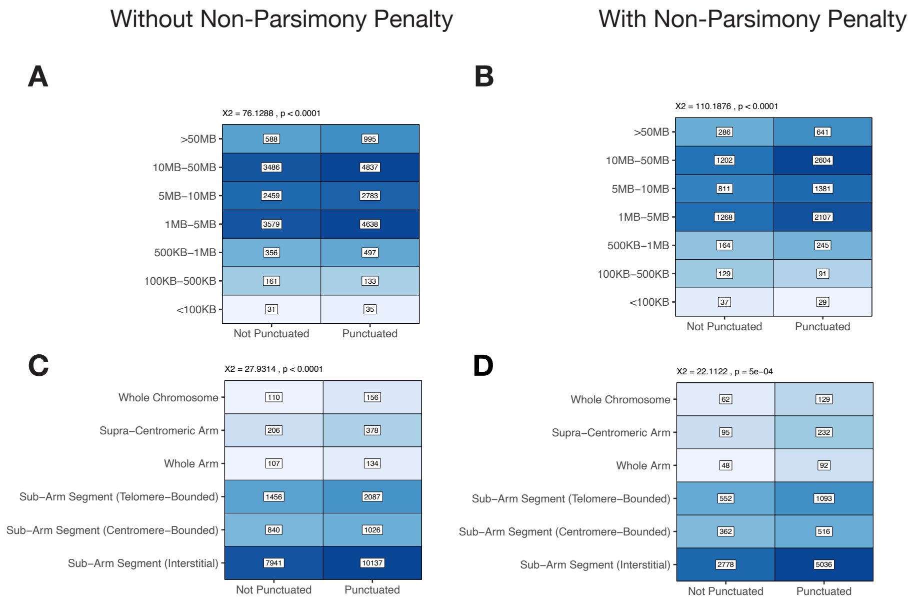

Figure S36: Genomic features of punctuated gains. **A** Size distributions of gains associated and not-associated with punctuated gains, without a penalty on non-parsimony applied during inference. **B** Size distributions of gains associated and not-associated with punctuated gains, with a penalty on non-parsimony applied during inference. **C**, Arm size classifications of gains associated and not-associated with punctuated gains, without a penalty on non-parsimony applied during inference. **D**, Arm size classifications of gains associated and not-associated with punctuated gains, with a penalty on non-parsimony applied during inference.

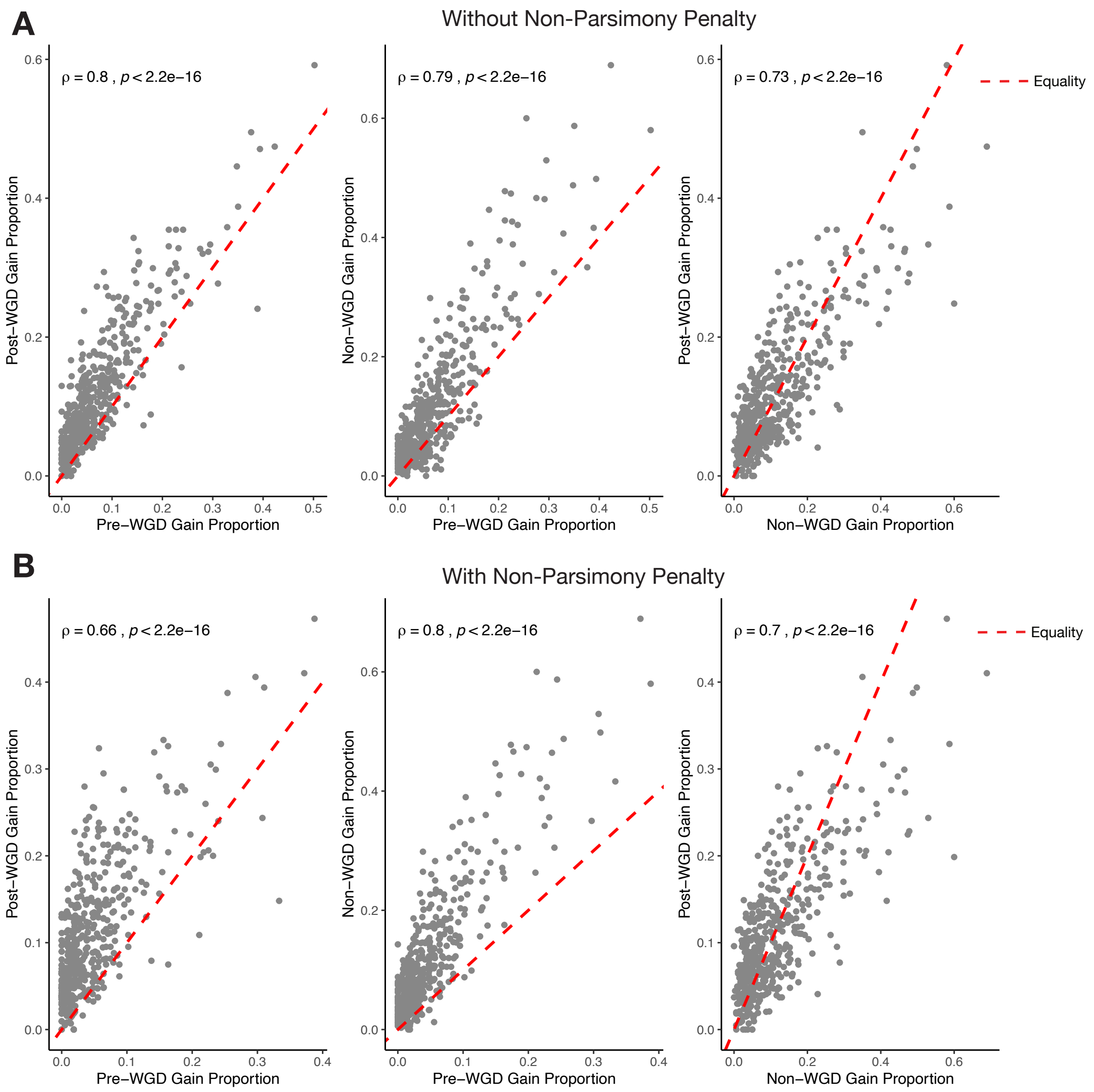

Figure S37: Frequency of arm gains pre and post-WGD and in non-WGD tumors. The proportion of samples in different cancer types that have gained different chromosome arms pre- and post-WGD and in non-WGD samples. Measured without (**A**) and with (**B**) a penalty on non-parsimony during inference. Correlation is measured using Spearman's correlation coefficient. Each point corresponds to the frequency of an arm gain in an individual cancer type.

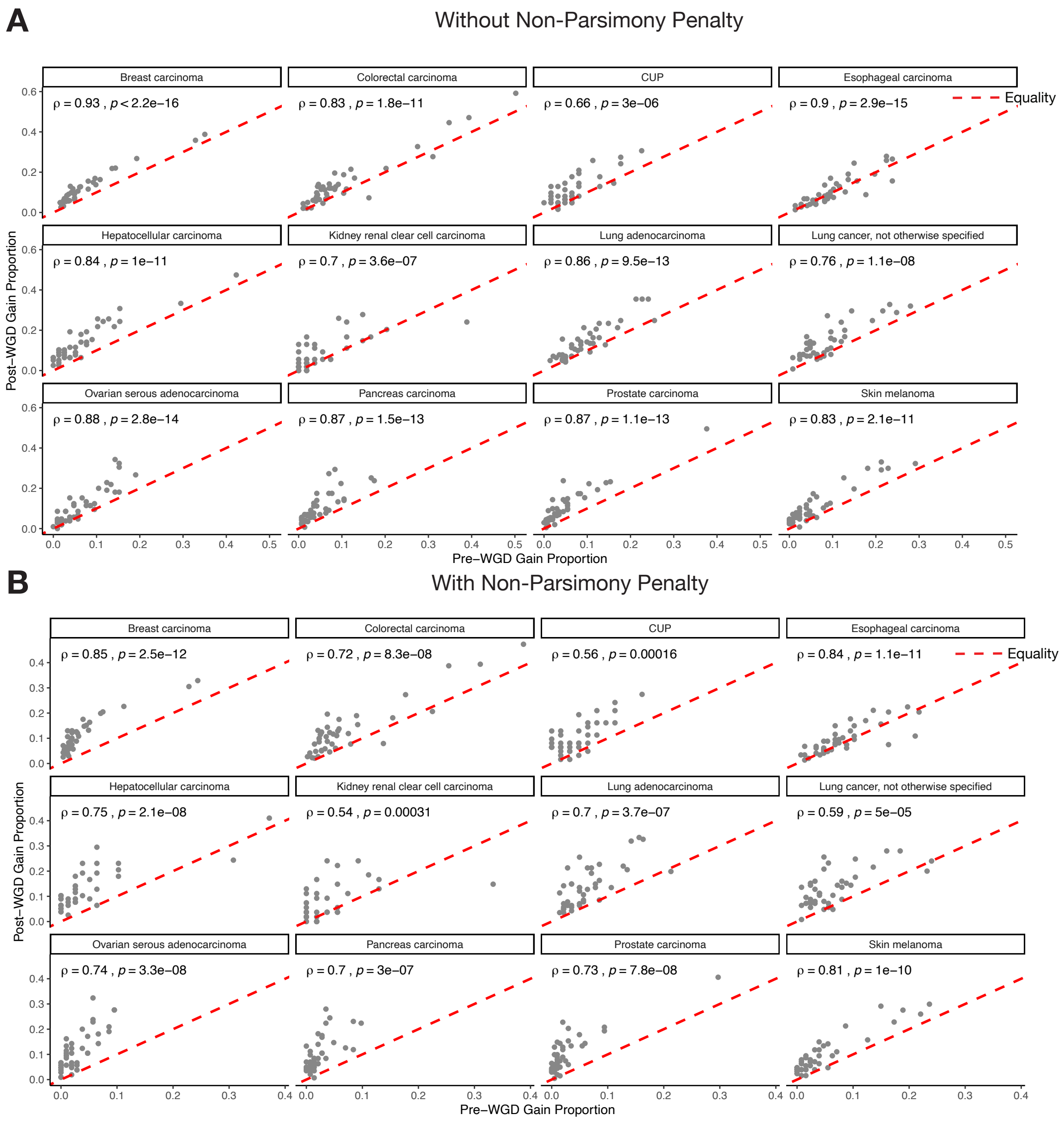

Figure S38: Frequency of arm gains pre and post-WGD and in non-WGD tumors by cancer type. The proportion of samples that have gained different chromosome arms pre- and post-WGD split by cancer type. Measured without (**A**) and with (**B**) a penalty on non-parsimony during inference. Correlation is measured using Spearman's correlation coefficient. Each point corresponds to the frequency of an arm gain relative to WGD in an individual cancer type.

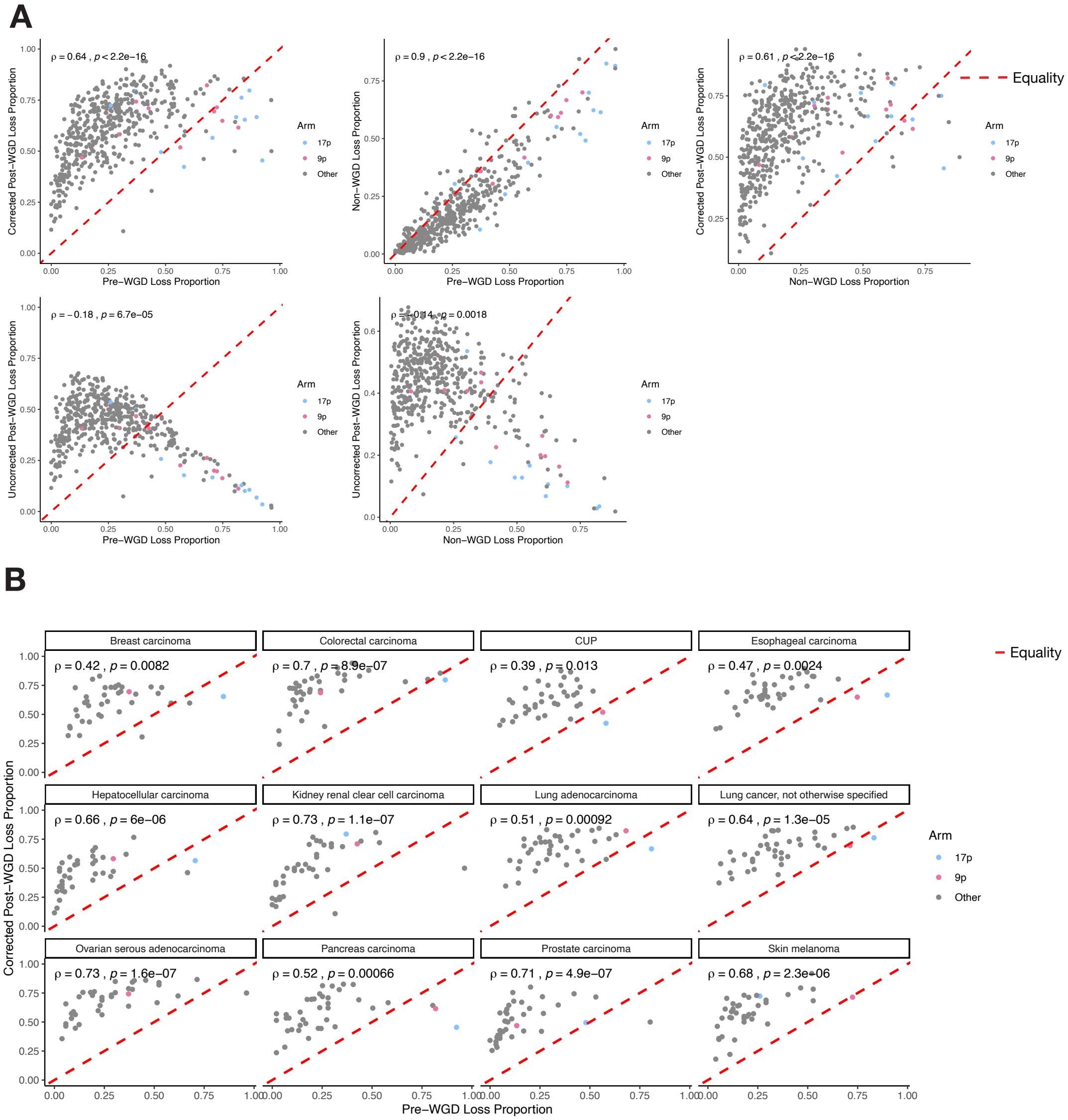

Figure S39: Frequency of arm losses pre and post-WGD and in non-WGD tumors by cancer type. **A**, The proportion of samples in different cancer types that have lost different chromosome arms pre- and post-WGD and in non-WGD samples. **B**, The proportion of samples that have lost different chromosome arms pre- and post-WGD split by cancer type. Correlation is measured using Spearman's correlation coefficient. Each point corresponds to the frequency of an arm loss relative to WGD in an individual cancer type.

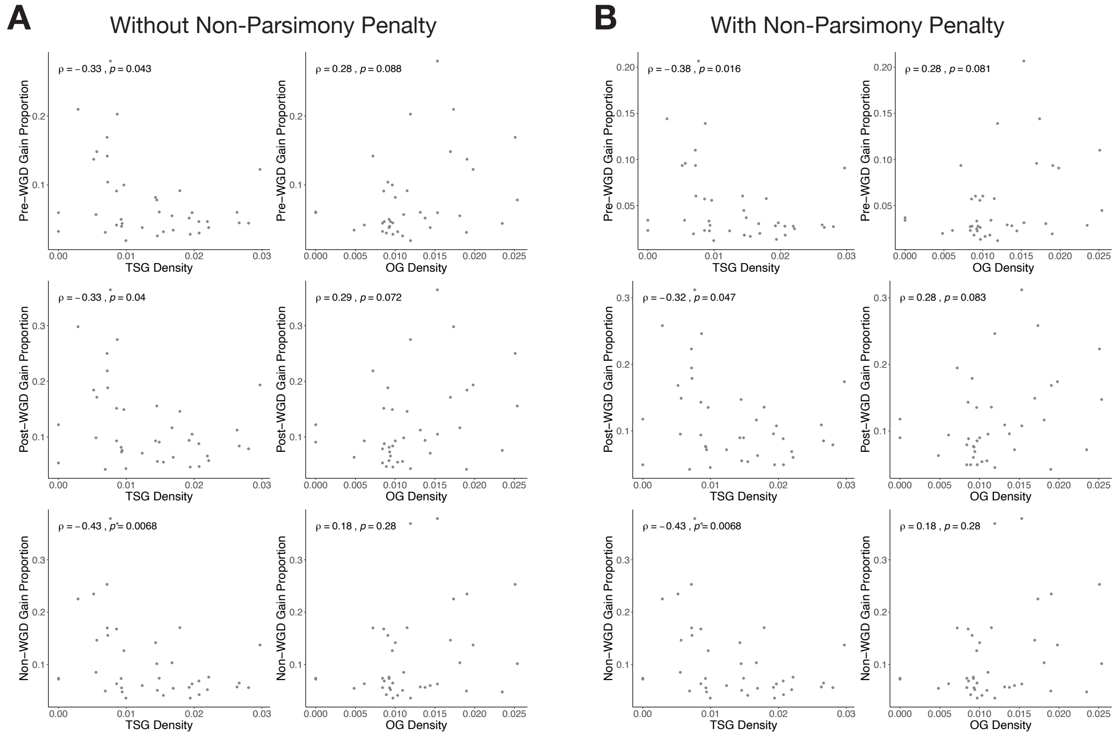

Figure S40: Effect of oncogene and tumor suppressor gene density on arm gain rates. The proportion of samples combined across cancer types that have gained different chromosome arms pre- and post-WGD and in non-WGD samples against arm oncogene (OG) and tumor suppressor gene (TSG) density. Measured without (**A**) and with (**B**) a penalty on non-parsimony during inference. Correlation is measured using Spearman's correlation coefficient. Each point corresponds to the frequency of an arm gain across the entire cohort.

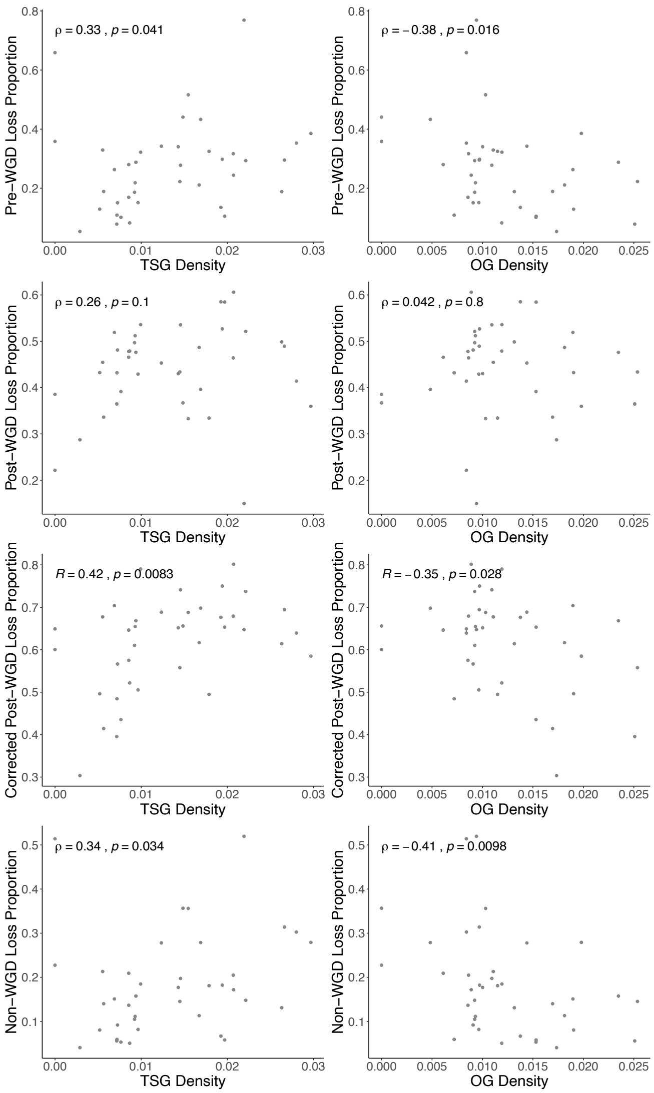

Figure S41: Effect of oncogene and tumor suppressor gene density on arm loss rates. The proportion of samples combined across cancer types that have lost different chromosome arms pre- and post-WGD and in non-WGD samples against arm oncogene (OG) and tumor suppressor gene (TSG) density. Correlation is measured using Spearman's correlation coefficient. Each point corresponds to the frequency of an arm loss across the entire cohort.

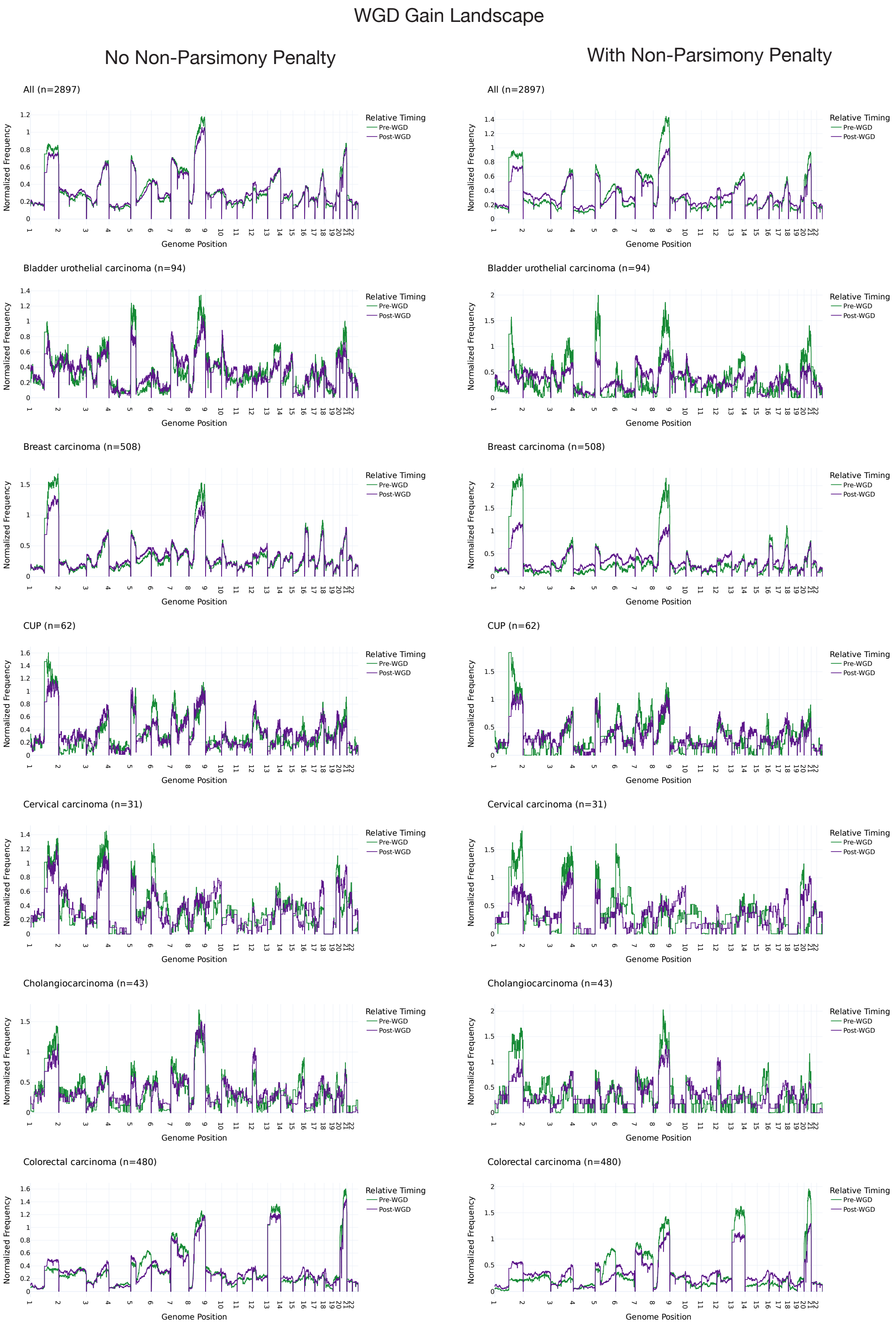

Figure S42: Pan-genome frequencies of pre and post-WGD gains by cancer type. Frequency of pre- and post-WGD gains for different cancer types. The frequencies are normalized so that the pre- and post-WGD frequency integrate to the same constant. Measured with and without a penalty on non-parsimonious routes during inference.

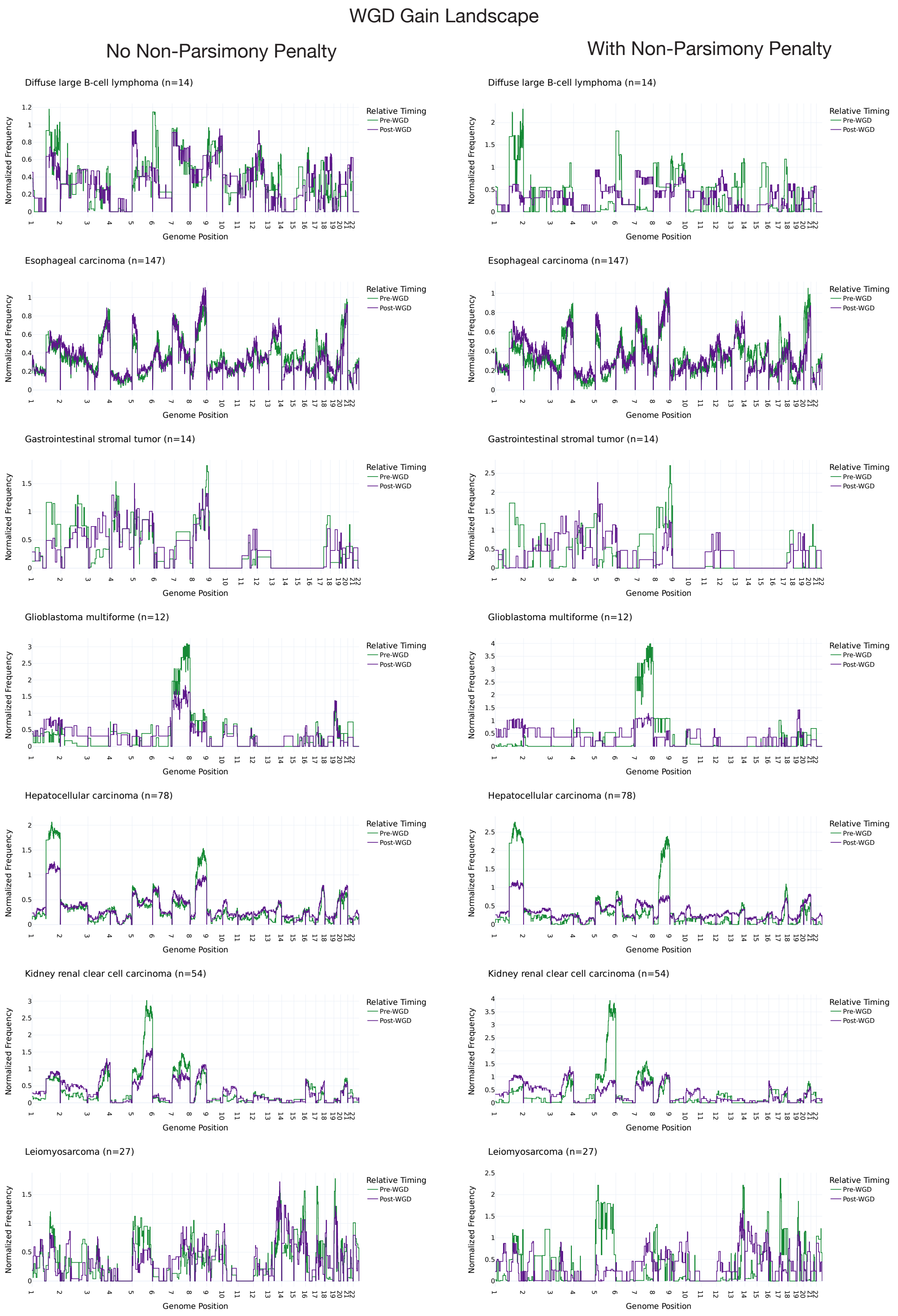

Figure S43: Pan-genome frequencies of pre and post-WGD gains by cancer type. Frequency of pre- and post-WGD gains for different cancer types. The frequencies are normalized so that the pre- and post-WGD frequency integrate to the same constant. Measured with (right column) and without (left column) a penalty on non-parsimonious routes during inference.

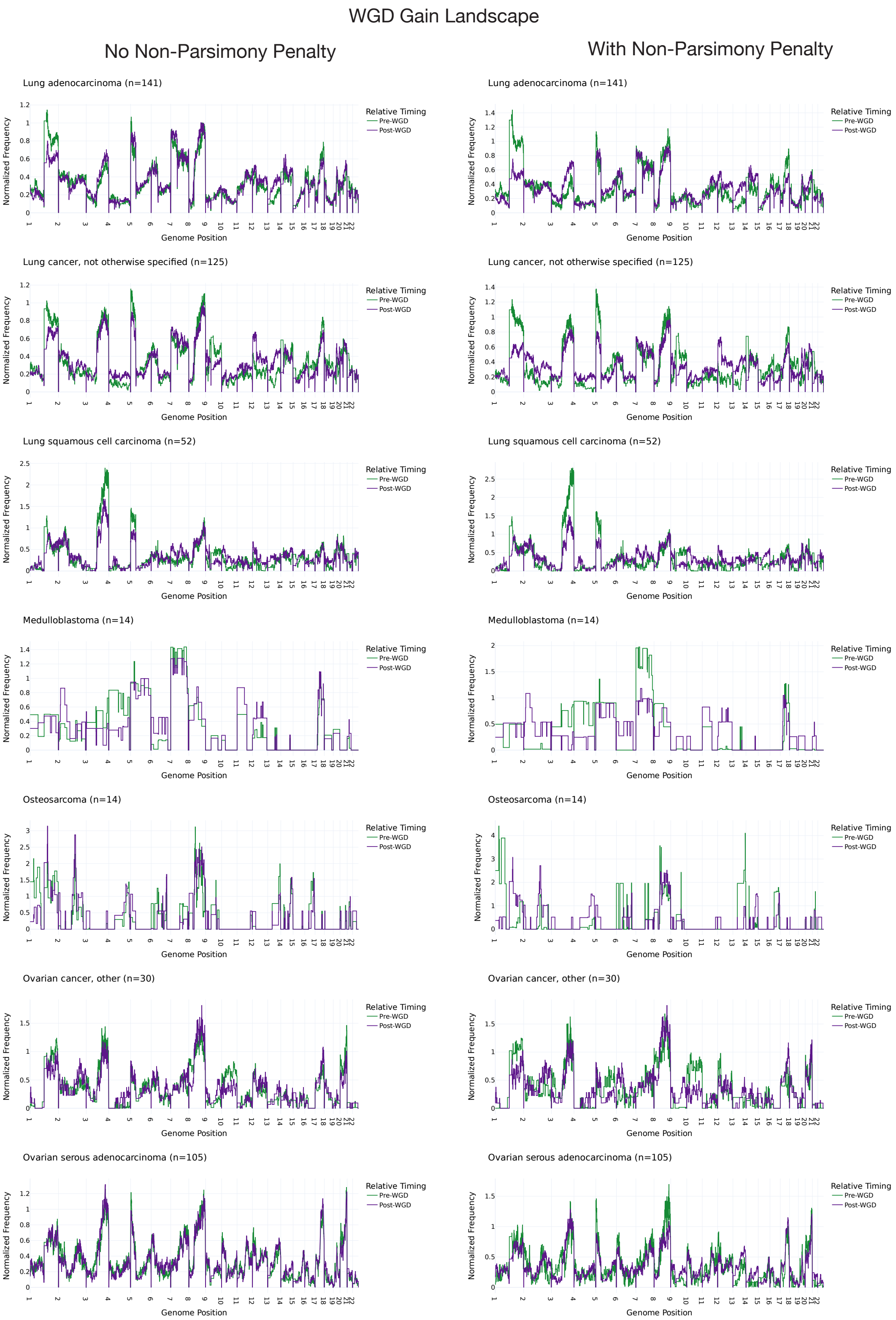

Figure S44: Pan-genome frequencies of pre and post-WGD gains by cancer type. Frequency of pre- and post-WGD gains for different cancer types. The frequencies are normalized so that the pre- and post-WGD frequency integrate to the same constant. Measured with (right column) and without (left column) a penalty on non-parsimonious routes during inference.

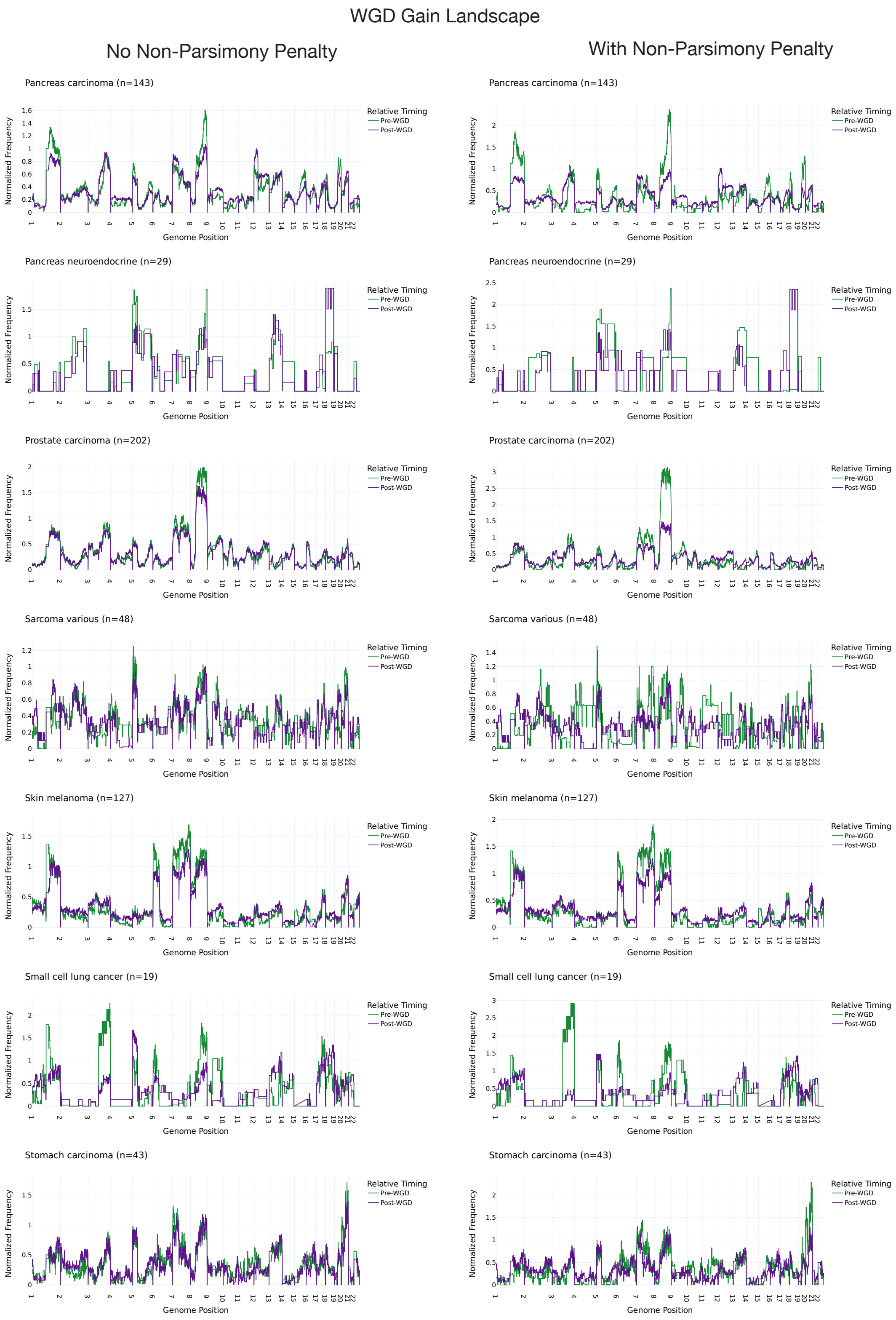

Figure S45: Pan-genome frequencies of pre and post-WGD gains by cancer type. Frequency of pre- and post-WGD gains for different cancer types. The frequencies are normalized so that the pre- and post-WGD frequency integrate to the same constant. Measured with (right column) and without (left column) a penalty on non-parsimonious routes during inference.

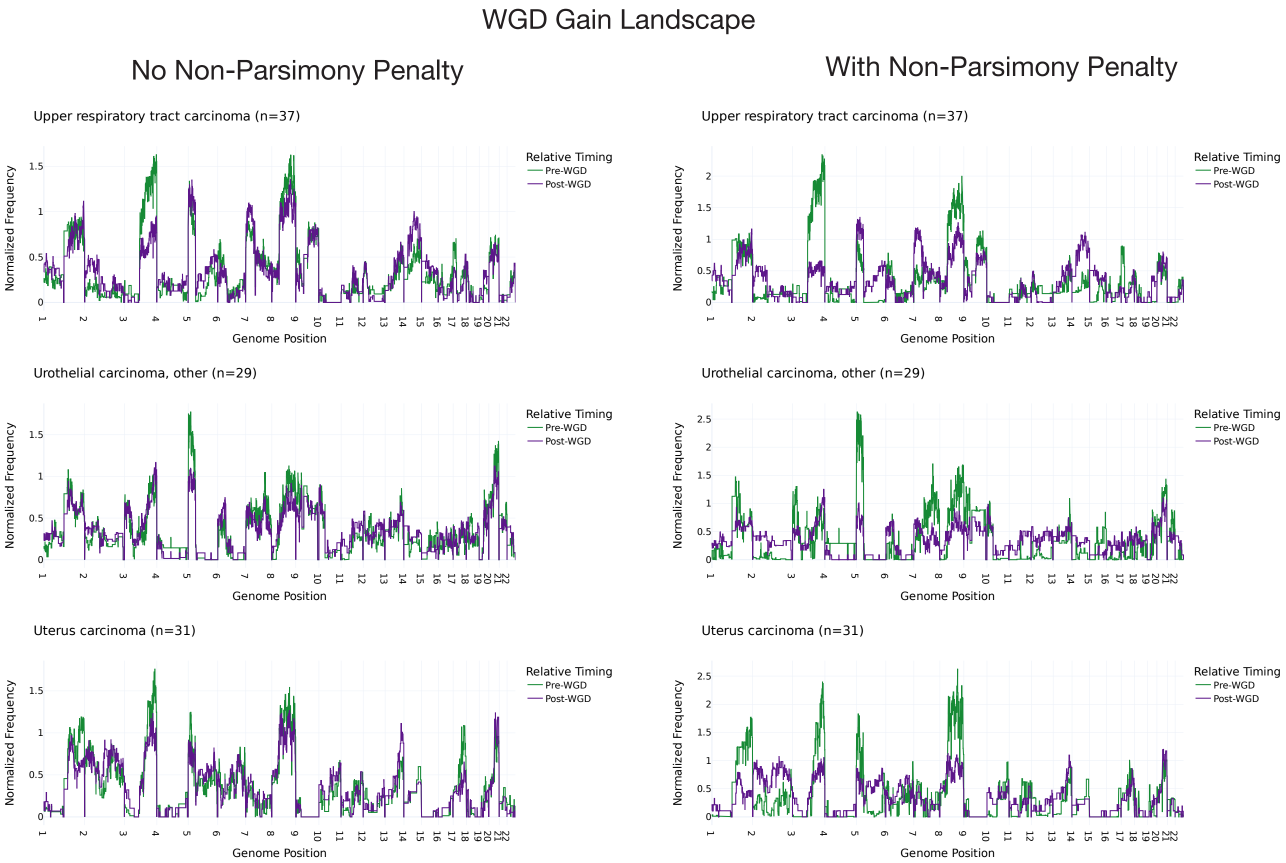

Figure S46: Pan-genome frequencies of pre and post-WGD gains by cancer type. Frequency of pre- and post-WGD gains for different cancer types. The frequencies are normalized so that the pre- and post-WGD frequency integrate to the same constant. Measured with (right column) and without (left column) a penalty on non-parsimonious routes during inference.

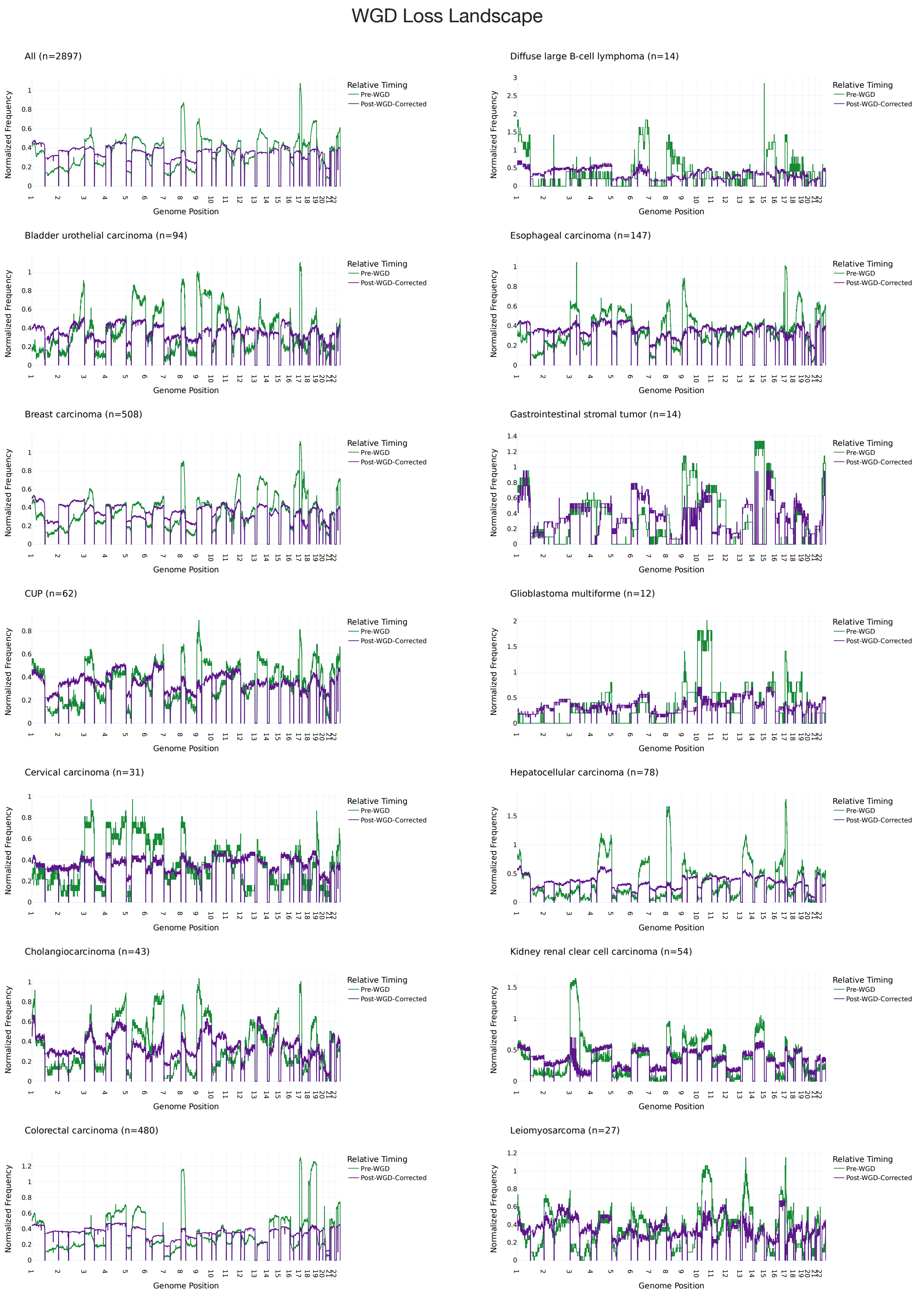

Figure S47: Pan-genome frequencies of pre and post-WGD losses by cancer type. Frequency of pre- and post-WGD losses for different cancer types. The frequencies are normalized so that the pre- and post-WGD frequency integrate to the same constant. The post-WGD loss frequency is corrected to account for mutual exclusivity when measuring pre- and post-WGD losses.

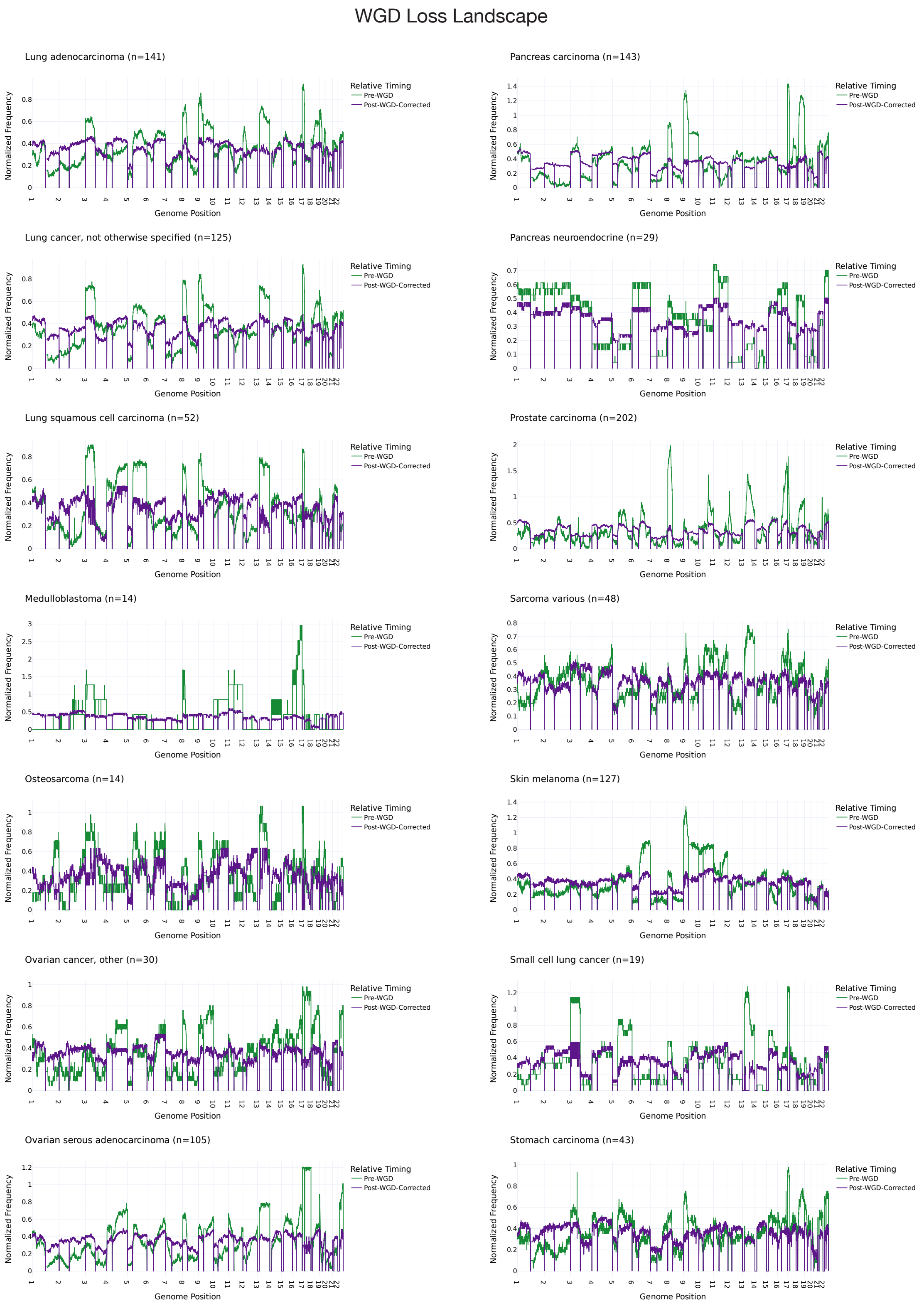

Figure S48: Pan-genome frequencies of pre and post-WGD losses by cancer type. Frequency of pre- and post-WGD losses for different cancer types. The frequencies are normalized so that the pre- and post-WGD frequency integrate to the same constant. The post-WGD loss frequency is corrected to account for mutual exclusivity when measuring pre- and post-WGD losses.

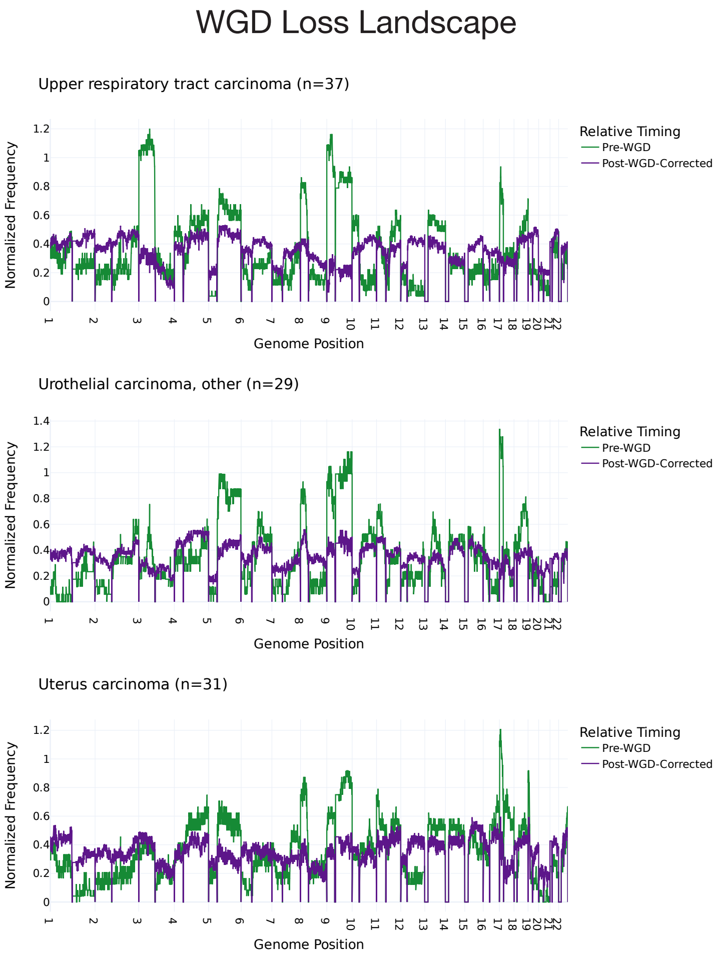

Figure S49: Pan-genome frequencies of pre and post-WGD losses by cancer type. Frequency of pre- and post-WGD losses for different cancer types. The frequencies are normalized so that the pre- and post-WGD frequency integrate to the same constant. The post-WGD loss frequency is corrected to account for mutual exclusivity when measuring pre- and post-WGD losses.

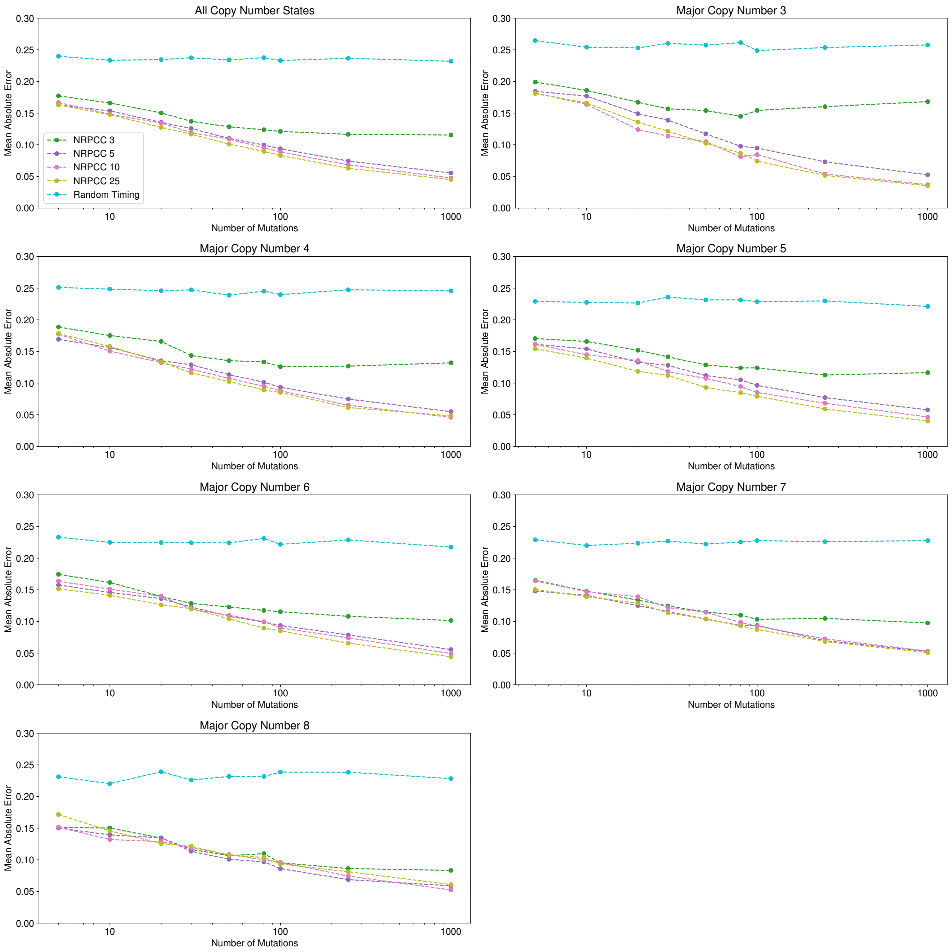

Figure S50: The effect of NRPCC and mutation count on gain timing inference. The mean absolute error in measuring the timing of independent copy number gains from simulated data for copy number segments with varying NRPCC and number of mutations per segment. The error is also calculated for a baseline random model where the true and measured timing values are permuted between segments of the same copy number.

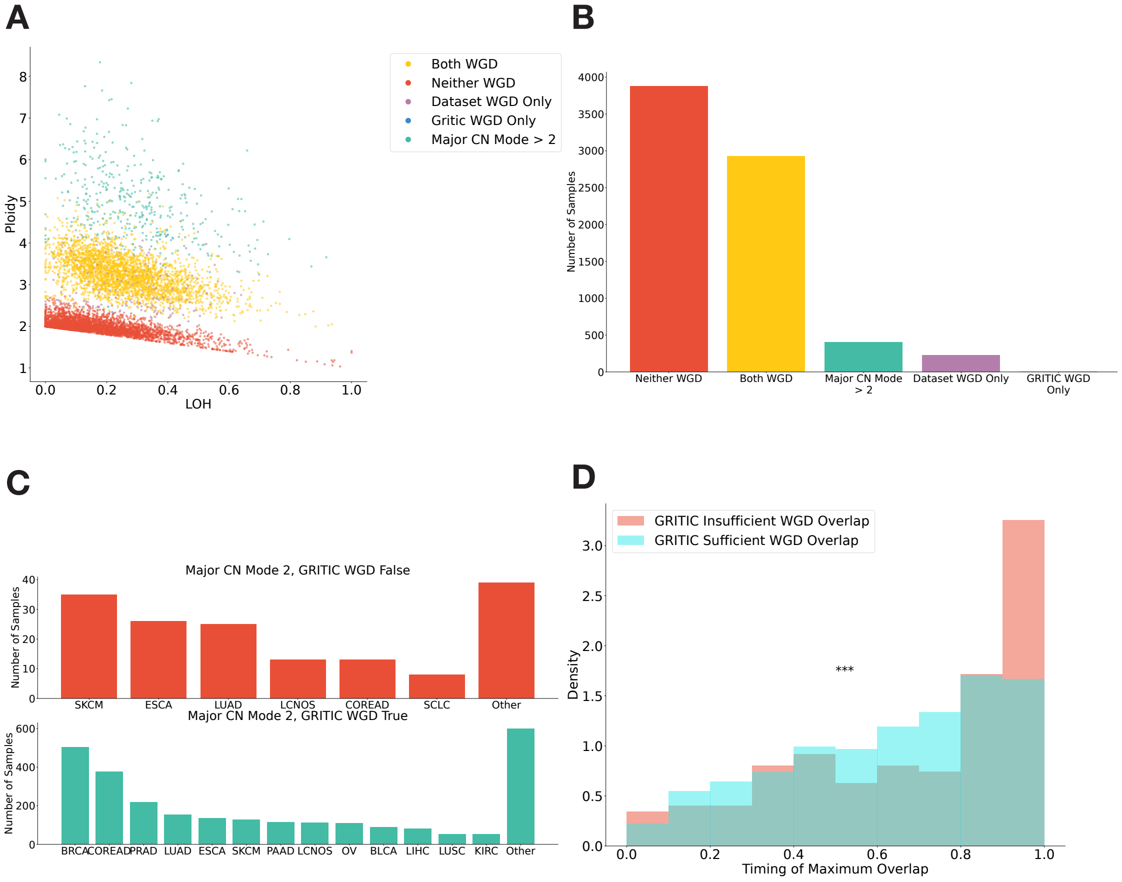

Figure S51: WGD status calling in GRITIC. A, Proportion of genome with loss of heterozygosity and tumor ploidy for samples in the PCAWG and Hartwig cohorts. Samples are colored by whether they were called as WGD by one or both of GRITIC and from their copy number profiles only. Samples with major copy number mode greater than of two are categorized separately as they are not supported by GRITIC. **B**, Counts of samples with different WGD calling statuses across Hartwig and PCAWG cohorts. **C**, Counts of tumor with major copy number two in PCAWG and Hartwig by cancer type. Cohort split by WGD calling status in GRITIC. **D**, The timing that maximally intersected the timing confidence intervals of major copy number two states. The distributions are split by whether such timing intersected with 60% of major copy number two segments by width. Only tumors with a major copy number mode of two are displayed. Statistical significance was calculated by Mann Whitney U Test. *** indicates a comparison where $p < 0.001$.

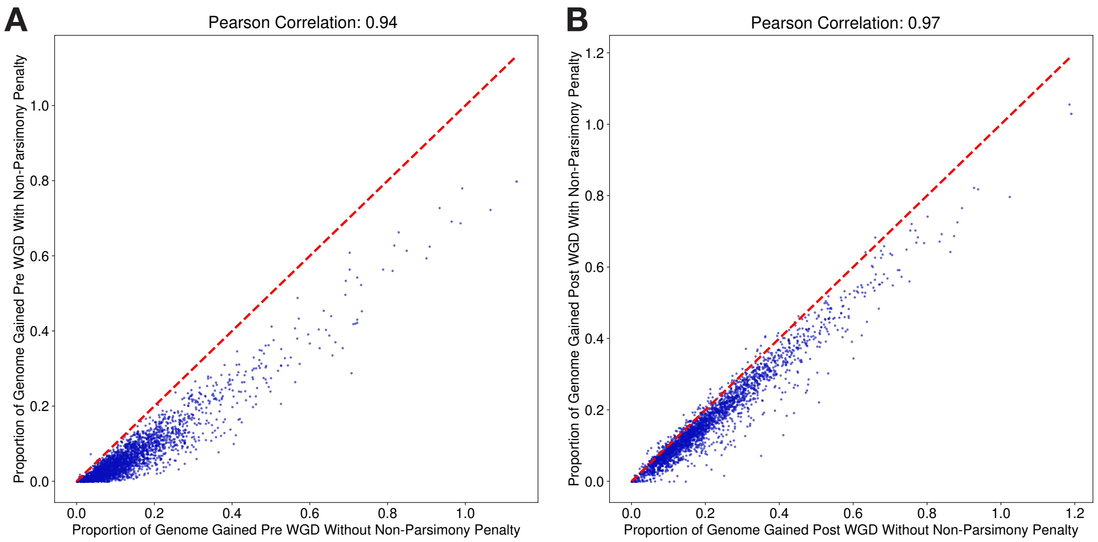

Figure S52: The effect of the non-parsimony penalty on event timing. The proportion of genome inferred as being gained pre- (**A**) and post-WGD (**B**) with and without a penalty on non-parsimony being applied.
